# London dispersion driven compaction of coordination cages in the gas-phase – a combined ion mobility and theoretical study†

**DOI:** 10.1039/d4sc04786a

**Published:** 2024-10-14

**Authors:** Christoph Drechsler, Ananya Baksi, André Platzek, Mert Acar, Julian J. Holstein, Christopher J. Stein, Guido H. Clever

**Affiliations:** a Department of Chemistry and Chemical Biology, TU Dortmund University Otto-Hahn-Str. 6 44227 Dortmund Germany guido.clever@tu-dortmund.de; b Department of Chemistry, Jadavpur University Kolkata-700032 West Bengal India; c Technical University of Munich, TUM School of Natural Sciences and Catalysis Research Center, Department of Chemistry Lichtenbergstr. 4 85748 Garching Germany

## Abstract

Large self-assembled systems (such as metallosupramolecular rings and cages) can be difficult to structurally characterize, in particular when they show a highly dynamic behavior. In the gas-phase, Ion Mobility Spectrometry (IMS), in tandem with Electrospray Ionization Mass Spectrometry (ESI MS), can yield valuable insights into the size, shape and dynamics of such supramolecular assemblies. However, the detailed relationship between experimental IMS data and the actual gas-phase structure is still poorly understood for soft and flexible self-assemblies. In this study, we combine high resolution Trapped Ion Mobility Spectrometry (TIMS), yielding collisional cross section data (CCS), with computational modeling and theoretical CCS calculations to obtain and interpret gas-phase structural data for a series of palladium-based coordination cages. We focus on derivatives of a homoleptic lantern-shaped [Pd_2_L_4_]^4+^ cage and its interpenetrated dimer ([3X@Pd_4_L_8_]^5+^, X = Cl, Br) to study the influence of flexible side chains of different lengths, counter anions and π-stacking tendencies between the ligands in the absence of solvent. The gained insights as well as the presented CCS calculation and evaluation workflow establish a basis for the systematic gas-phase characterization of a wider range of flexible, chain-decorated and guest-modulated assemblies.

## Abstract 2

Large self-assembled systems (such as metallosupramolecular rings and cages) can be difficult to structurally characterize, in particular when they show a highly dynamic behavior.

## Graphical abstract

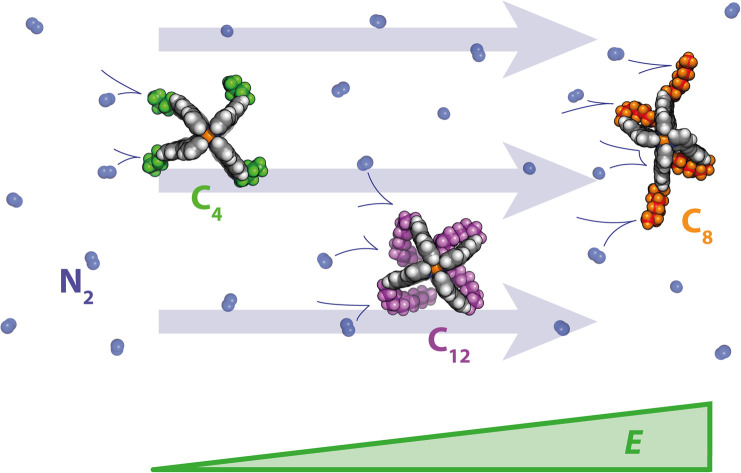

## Introduction

The targeted encapsulation of smaller molecules by larger, hollow structures has application potential in areas such as enzyme-like catalysis and selective compound separation.^1–6^ Of special interest are systems that can be obtained *via* self-assembly from simple molecular building blocks of preprogrammed shape and connectivity, thus avoiding long and inefficient synthetic routes. Over the past few years, a plethora of nanosized metallo-supramolecular cages based on multidentate ligands and metal nodes such as palladium(ii) cations have been developed and investigated.^7–19^ For several decades, the characterization of large supramolecular structures has relied on X-ray crystallography (solid state), NMR spectroscopy (solution) and mass spectrometry (gas-phase). In particular, the latter methodology has seen a steep development in recent years, with ionization techniques becoming milder (*e.g.* in Cryospray Ionization, CSI), detectors becoming more accurate and orthogonal detection methods being online-combined with classic *m*/*z* determination. Among the latter approaches, Ion Mobility Spectrometry (IMS) has turned out to be a powerful tool that can yield significant structural insight into the gas-phase structure and dynamics of larger self-assemblies.^20–25^ Recently, we demonstrated the power of Trapped Ion Mobility Spectrometry (TIMS)^26–28^ in tandem with ESI mass spectrometry for the analysis of complex mixtures of multiple different heteroleptic cages.^29^ On the one hand, IMS can deliver useful qualitative (larger/smaller) information, *e.g.* to compare isomeric assemblies or to locate a guest inside or outside a host structure. In this respect, its power to resolve minor size differences is much higher than that for solution-bound DOSY NMR. Measured gas-phase ion mobility values *K*, however, contain much more precious information, as they can be transformed into a Collisional Cross Section (^TIMS^CCS_N_2__, if N_2_ is used as carrier gas), which can, in simple terms, be understood as the averaged projected 2D area of the ion.^30,31^ Comparing this experimental value with corresponding theoretical CCS estimates (^theo^CCS_N_2__), calculated from *in silico* models of an assumed set of 3D structures, yields much more detailed insights into gas-phase structures and can not only help to differentiate but also assign isomers,^23^ unravel co-conformations of non-covalent adducts^32^ and elucidate guest-modulated structural changes of a host compound.^33^

A number of different ^theo^CCS calculation procedures have been implemented in various programs.^34,35^ The currently most accurate and therefore most often used method is the Trajectory Method (TM), which simulates collisions between an ion and a large number of carrier gas molecules. During this calculation, the ion is fixed and the impacting gas molecule experiences a potential consisting of a Lennard-Jones (LJ) term and a term for ion-induced dipole interactions. This approach, however, is error prone for flexible analytes because it neglects molecular motion; therefore it is often combined with Molecular Dynamics (MD) simulations.^36–40^ The latter are used to generate a conformational ensemble which better describes the gas-phase situation of a flexible analyte than what is achievable with a single optimized structure. The ^theo^CCS is then determined for each conformer and a Boltzmann-weighted average is calculated.

In the field of (metallo-)supramolecular chemistry, so far only a few IMS studies have combined conformational analysis with CCS simulation methods.^38–40^ Yet, countless self-assemblies that are intrinsically flexible or carry floppy solubilizing chains are produced in the research field on a regular basis, which creates demand for new analytical and computational workflows to properly characterize these compounds in the gas-phase. Also, to the best of our knowledge, no one has described this for metal-mediated self-assemblies beyond a molecular mechanics level of theory. With access to new fast and robust semiempirical methods such as GFN2-xTB, which has become popular for the optimization of larger structures and non-covalent complexes, we see large potential for the use of IMS together with a computational interpretation workflow to enrich the routine analytics of large and flexible assemblies.^41,42^

In this study, we examine the power of a combined experimental and computational IMS workflow by applying it to four different problems, all deriving from a common molecular building block, but highlighting a variety of effects. In particular, we study (1) a wide-meshed, flexible cage carrying four alkyl chains of different lengths, (2) its densely packed, catenated dimer with eight crowded chains, (3) guest-modulated minor size variations of these dimers and (4) systems with aryl instead of alkyl substituents.

**Fig. 1:**
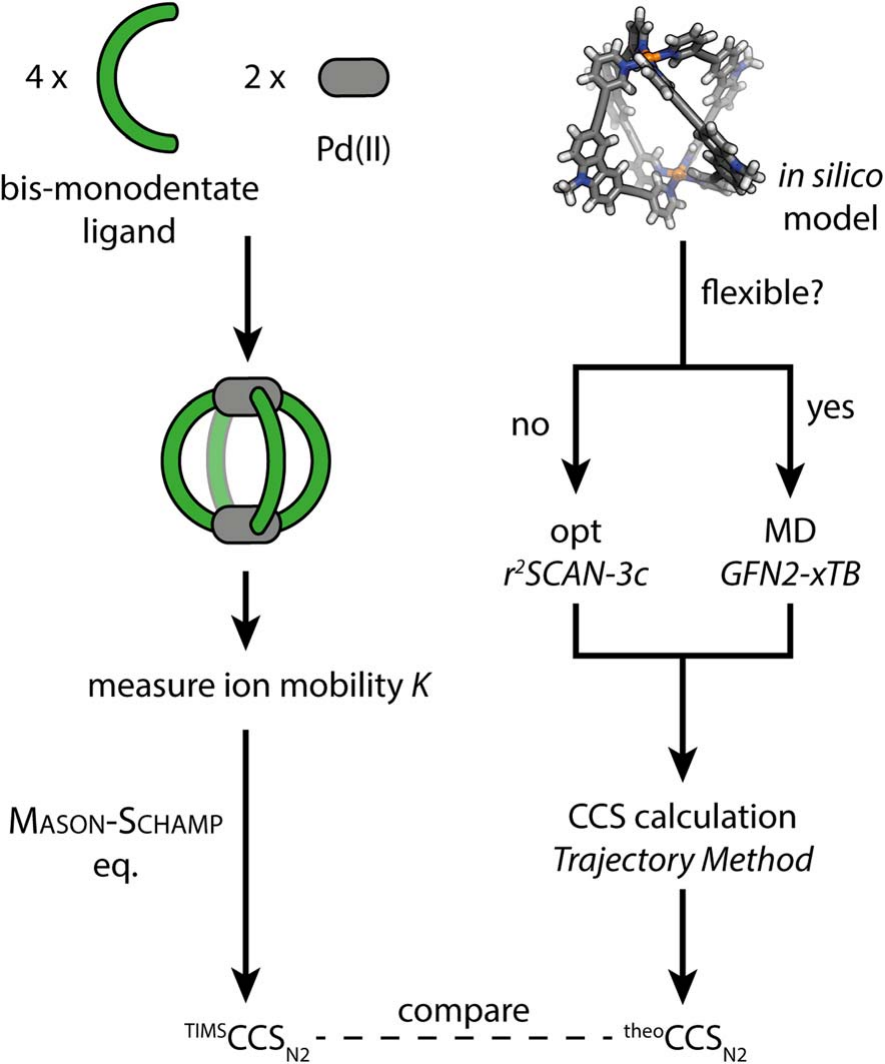
Schematic workflow for the gas-phase structure examination of coordination cages *via* ion mobility spectrometry and theoretical CCS calculation.

(1) As a first model system we chose a simple, homoleptic, lantern-shaped [Pd_2_L_4_]^4+^ cage, published by our group before.^43^L is a bent, bis-monodentate organic ligand consisting of a carbazole backbone, two pyridine donor groups and alkyne spacers. Common for supramolecular building blocks, the central nitrogen carries an alkyl chain to increase solubility. Here, we chose to install several alkyl chains of different lengths to study their influence on the gas-phase structure of the assembly, as expressed by the experimental and theoretical CCS values. While the conformational space of simple alkanes in the gas-phase is well understood,^44^ the behavior of flexible groups attached to larger and structurally more complex assemblies is rarely considered in detail, which causes difficulties in terms of interpretation of experimental and calculation of theoretical CCS values. We herein address this problem *via* a systematic approach, employing high resolution TIMS measurements and a computational workflow sketched in Fig. 1. Additionally, the trapped ion mobility results are compared to solvodynamic radii obtained from ^1^H DOSY NMR measurements.

(2) Upon addition of either Cl^−^ or Br^−^ anions, cages [Pd_2_L_4_]^4+^ dimerize to large, interpenetrated catenanes ([3X@Pd_4_L_8_]^5+^, X = Cl, Br) with three small pockets. Pleasingly, this gives us easy access to another systematic series of compounds to characterize in the gas-phase whose alkyl side chain behavior can be compared to that of the monomeric cages.

(3) Previously, we showed by ^1^H DOSY NMR that the combination of anions ([BF_4_^−^]_3_*vs.* [Cl^−^ + BF_4_^−^ + Cl^−^]), encapsulated in the three pockets, has an influence on the overall size of the interpenetrated structure.^45^ But DOSY NMR does not offer a high enough resolution to catch the difference caused by using either Cl^−^ or Br^−^ on the overall structure. We herein challenged whether the corresponding small size differences can be resolved with TIMS and reproduced with the combination of MD simulation and ^theo^CCS_N_2__ calculations.

(4) London-dispersive interactions between neighboring moieties in flexible assemblies can become so dominant in the gas-phase that certain conformers, *i.e.* folded states, may end up being energetically strongly favored as compared to less compacted structures found in solution. When dispersion-corrected DFT methods are used in gas-phase geometry optimizations of such systems, they often converge towards a folded conformation, for example, when intramolecular π-stacking governs the structure. However, it is not always clear whether gas-phase experimental and DFT data yield the same answer concerning the degree of folding in a flexible molecule. Here, we compare a series of [Pd_2_L_4_]^4+^ cages with alkyl and aryl substituents to investigate how the increase in π-surface area influences the degree of gas-phase compaction in the IMS experiment *vs.* the results of various computational approaches.

## Results and discussion

**Fig. 2:**
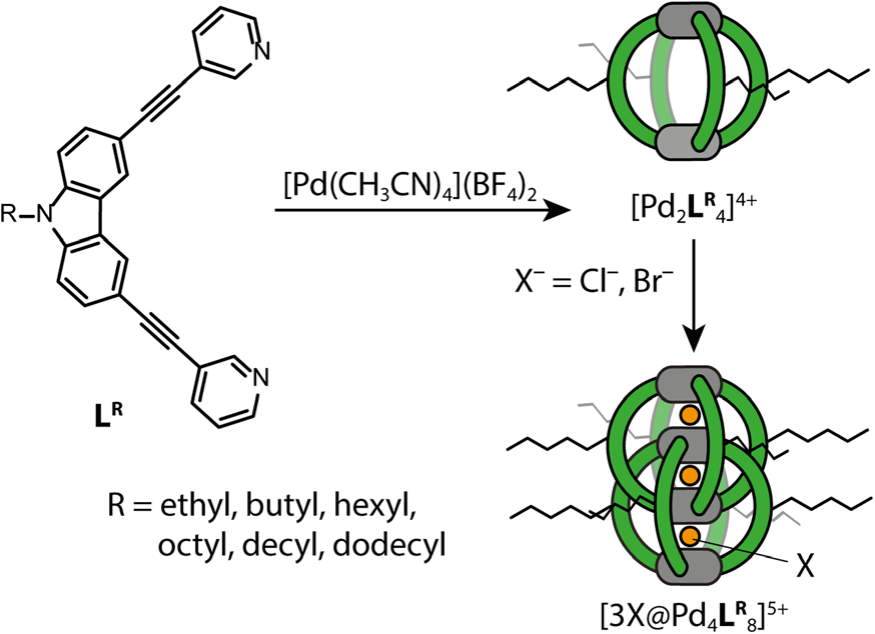
Synthesized and analyzed monomeric and dimeric cages.

To get an idea of how (alkyl) side chains of coordination cages behave in the gas-phase as compared to solution, both experimentally and in the computational models, we synthesized the banana-shaped bis-monodentate ligand L^R^ with different side chains ranging from ethyl to dodecyl in steps of two methylene groups. As shown before, L^R^ forms a [Pd_2_L^R^_4_]^4+^ cage in MeCN with BF_4_^−^ as counter anions upon addition of [Pd(MeCN)_4_](BF_4_)_2_.^43^ When 1.5 eq. Cl^−^ or Br^−^ is added, dimerization to an interpenetrated double cage ([3X@Pd_4_L^R^_8_]^5+^, X = Cl, Br) is observed, although not exclusively. The equilibrium between the monomeric cage [Pd_2_L^R^_4_]^4+^, the double cage [3X@Pd_4_L^R^_8_]^5+^ and insoluble rings and catenanes {*trans*-[(PdX_2_)_2_L^R^_2_]}_*n*_ (*n* = 1, 2, 3) can be controlled by the amount of halide added.^35^ An overview of the synthesized ligands and investigated systems is given in Fig. 2.

The samples assembled in acetonitrile solution were then subjected to ESI trapped ion mobility-TOF analysis on a Bruker timsTOF mass spectrometer using N_2_ as the carrier gas (for details see the ESI†).

### Gas-phase alkyl side chain behavior for (1) monomeric and (2) dimeric cages

**Fig. 3:**
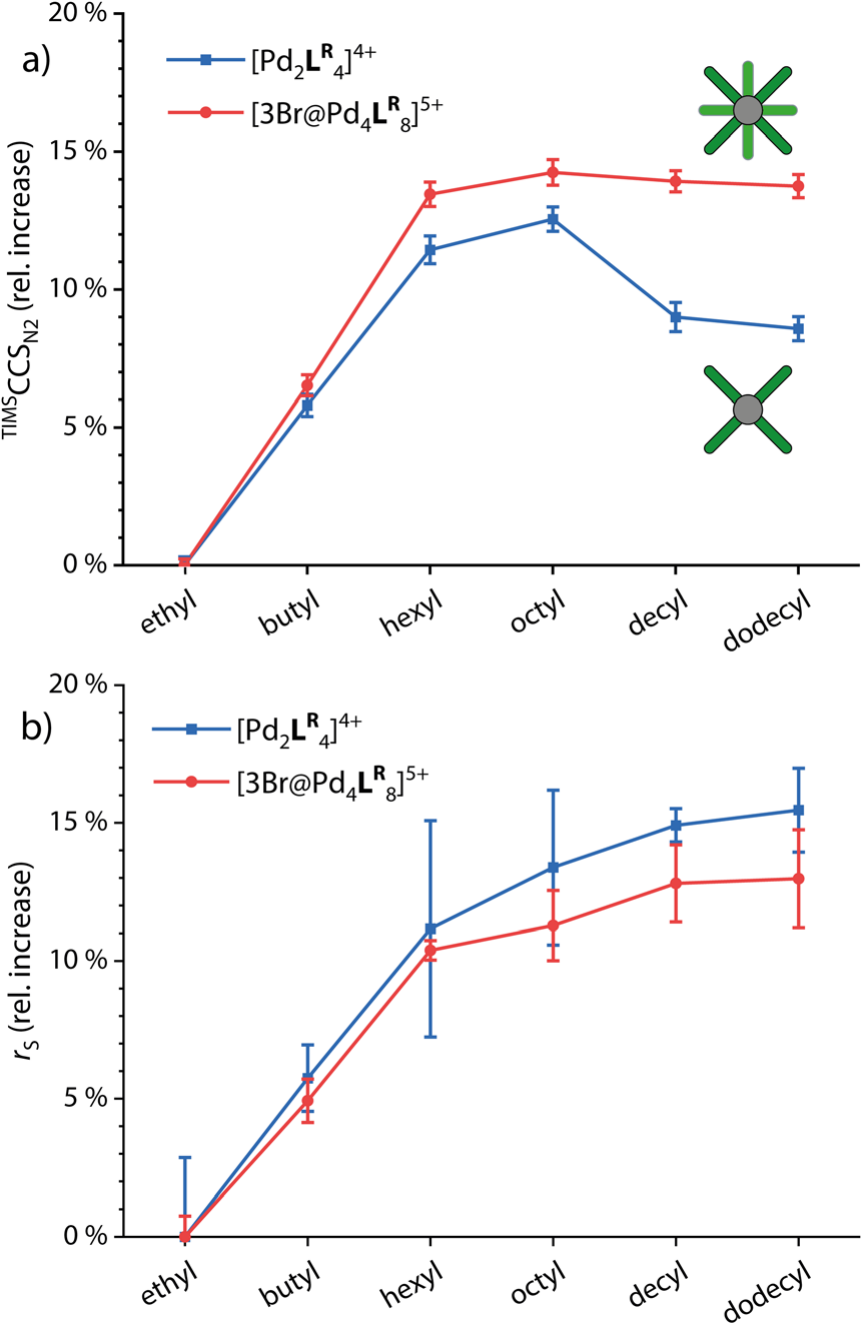
(a) Relative increase of experimental ^TIMS^CCS_N_2__ values and (b) relative increase of solvodynamic radii based on DOSY NMR for the monomeric and double cages (taking the systems with ethyl side chains as a reference).

For comparing the behavior of different side chain lengths decorating the monomeric and dimerized cages, we concentrated on the 4+ species for the monomeric cages and the 5+ species with three Br^−^ anions in the cavities for the double cages. The according ion mobilities were measured for each system and transformed into ^TIMS^CCS_N_2__ values using the Mason–Schamp equation (Fig. S4, S8 and Table S5†). Fig. 3a shows the relative increase of CCS values with growing side chains for the monomeric and double cages (taking the systems with ethyl side chains as a reference).

While CCS values increase linearly up to the derivatives with hexyl chains for both the monomeric and the double cages, the increase from hexyl to octyl for both is rather small indicating slight backfolding of the alkyl chains towards the cage, most likely due to London dispersion interactions. From octyl to decyl, the CCS clearly decreases for the monomeric cage while it remains more or less the same for the double cage. As an explanation for this observation, we suggest that the monomeric cages provide large gaps between neighboring ligands in which the backfolding chains can “hide”, resulting in a drop in CCS values with an increasing backfolding tendency (driven by an increase in dispersive attraction). The denser core structure of the double cage, however, provides only smaller crevices and therefore the stronger backfolding only merely compensates for the increase in chain length. Hexyl chains seem to be not folding back at all, in contrast to dodecyl chains which are apparently completely clinging to the cages. In addition, we measured ^1^H DOSY NMR spectra in CD_3_CN of all investigated systems. Here, we observe a steady increase in the solvodynamic radius with increasing chain length up to dodecyl and very similar behavior of monomeric and double cages. The curves seem to flatten for longer chains, explainable by the onset of random curling of the longer chains due to higher degrees of freedom. The interactions between the chains and the cage core, however, seem to be neglectable in solution due to competitive interactions with solvent molecules. Understanding the differential behaviour of chains of different lengths in the solution- *vs.* the gas-phase is of high importance for reliably interpreting DOSY *vs.* the ion mobility results of such species and drawing conclusions about their preferred conformations in both media. The ^1^H DOSY spectra are shown in Fig. S9 to S14† and the diffusion coefficients and solvodynamic radii are listed in Table S1.†

**Fig. 4:**
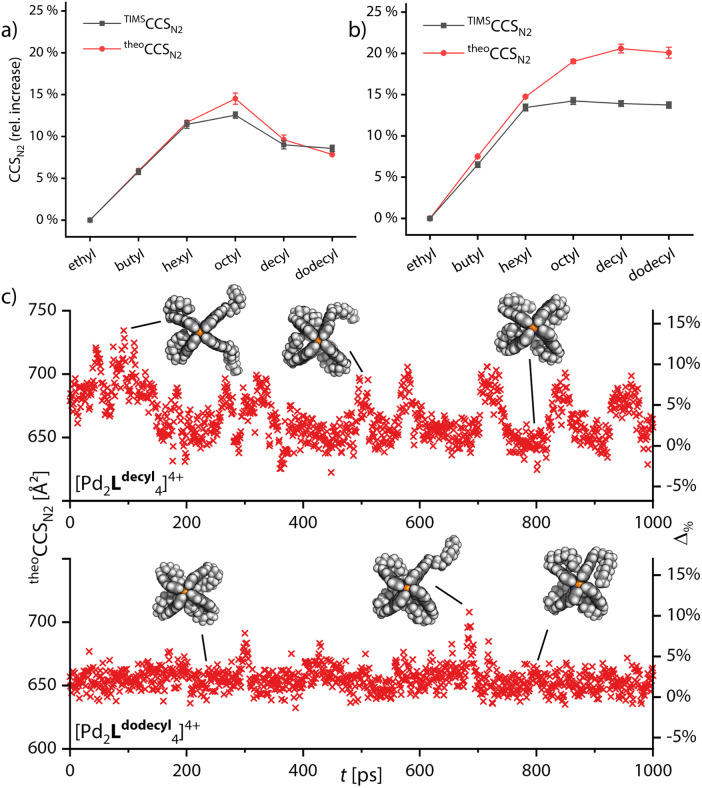
Relative increase of ^theo^CCS_N_2__ values calculated from the snapshots taken every picosecond from the MD simulations averaged over time (red) and compared with experimental values (black) for (a) monomeric cage [Pd_2_L^R^_4_]^4+^ and (b) double cage [3Br@Pd_4_L^R^_8_]^5+^. (c) ^theo^CCS_N_2__ values and respective relative deviations to the experiment plotted against time for two exemplary systems [Pd_2_L^decyl^_4_]^4+^ and [Pd_2_L^dodecyl^_4_]^4+^. Shown models are exemplary snapshots from the trajectory.

Next, we turned to simulating the behavior of the side chains by combining the GFN2-xTB computational approach with the trajectory method for CCS simulation. Semiempirical gas-phase MD simulations of all monomeric and double cages with different chain lengths were conducted for a time span of 1000 ps. Starting structures for the main MD run were obtained by preliminary MDs, commencing from a variety of manually set chain conformations, in which the systems were allowed to equilibrate. The ^theo^CCS_N_2__ were then calculated, using Collidoscope,^46^ from snapshots taken from the trajectory of the main MD run every picosecond. Obtained ^theo^CCS_N_2__ values and the relative differences plotted against the time are shown in Fig. S35–S37† and the resulting averaged theoretical values compared to the experimental ones are shown in Fig. 4a and b.

As Fig. 4a shows, we were able to adequately reproduce the trend for the monomeric cage. The averaged theoretical values match the experimental ones almost perfectly, when considering the relative CCS increase compared to [Pd_2_L^ethyl^_4_]^4+^. The highest deviation is found for [Pd_2_L^octyl^_4_]^4+^; here it seems that the balance between extended and backfolded chains could not be perfectly reproduced by GFN2-xTB. In Fig. 4c and d, we show two exemplary plots of MD-derived ^theo^CCS_N_2__ values over time and the resulting relative deviations from the respective ^TIMS^CCS_N_2__ result, for [Pd_2_L^decyl^_4_]^4+^ and [Pd_2_L^dodecyl^_4_]^4+^. The graphs show that the theoretical CCS values for the cage with decyl chains are heavily oscillating while they remain rather constant for dodecyl. This strengthens our hypothesis that the shorter decyl chains are still alternating between extended and compacted conformers while the longer dodecyl chains are more tightly bound towards the apertures of the cage. For the double cages (Fig. 4b) we obtained well matching values up to [3Br@Pd_4_L^hexyl^_8_]^5+^. While the theoretically derived curve then approaches a plateau, as observed for the experimental curve, the averaged ^theo^CCS_N_2__ values for the double cages with longer chains get too large as compared to the experimental values. Here it seems that GFN2-xTB could not properly reproduce the balance between extended and backfolded conformations. The accuracy of the simulations might also suffer from a rather short simulation time. As opposed to the wide-meshed monomeric cages, the optimal positions for the longer alkyl chains in the backfolded state may be harder to realize in the intercalated cages with a more complex surface structure. However, the systems' large sizes forced us to limit MD simulation times and prohibited application of deeper conformational sampling methods, for example using CREST.^47^

### Guest-controlled extension of double cages (3)

**Fig. 5:**
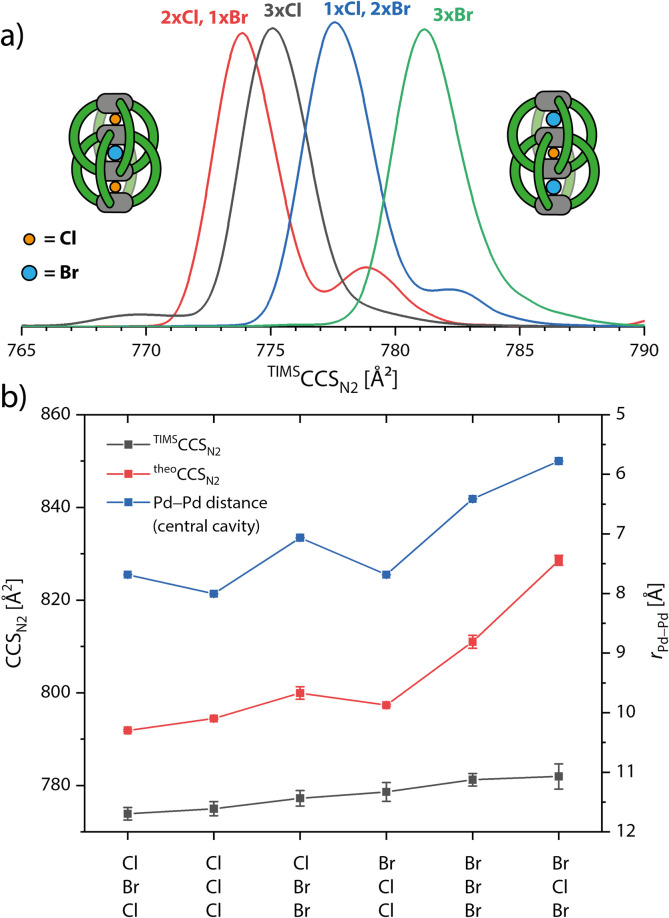
(a) Ion mobility spectra, converted to ^TIMS^CCS_N_2__, of the double cage with ethyl side chains and different combinations of halides in the three pockets ([3X@Pd_4_L^ethyl^_8_]^5+^, X = Cl, Br). (b) Plot of ^TIMS^CCS_N_2__, averaged ^theo^CCS_N_2__ values and averaged Pd–Pd distances (central cavity) as extracted from the MD runs.

When measuring *K* and *m*/*z* of the [3Br@Pd_4_L^R^_8_]^5+^ species, we could also detect the chloride-contaminated species [1Cl + 2Br@Pd_4_L^R^_8_]^5+^ and [2Cl + 1Br@Pd_4_L^R^_8_]^5+^ (Fig. S6†). This raised our interest and we complemented the series of halide-containing host–guest complexes (only for L^ethyl^) by templating the double cage with Cl^−^, exclusively, to obtain species [3Cl@Pd_4_L^ethyl^_8_]^5+^. The according ion mobility spectra are overlaid in Fig. 5. In previous studies we had shown that the occupation of the three cavities of interpenetrated double cages by different guests has an influence on the cage's dimension along the Pd_4_ axis.^45,48–50^ From our previous NMR and ESI MS measurements we know that the carbazole-based double cage can either bind three Cl^−^, three Br^−^ or two Cl^−^ and one Br^−^, where the single Br^−^ occupies the central cavity as evident from the signal pattern in the corresponding ^1^H NMR spectrum.^43^ This time, we were able to detect a fourth species with one Cl^−^ and two Br^−^ by ESI MS. While the IMS-derived size increase from species to species was found to be very small, high resolution TIMS measurements of the four described species could clearly differentiate between them. Interestingly, for the two mixed species containing both Cl^−^ and Br^−^, two signals appeared in their ion mobility traces, one major and one minor, indicating that both possible isomers (Br–Cl–Br *vs.* Br–Br–Cl, analogous for the 2Cl + Br species) with slightly different CCS values can be detected. We assume that for the species containing two Cl^−^ and one Br^−^, the species with Br^−^ in the middle pocket has a smaller CCS, because a size increase of the middle pocket by the slightly larger Br^−^ and a decrease of the outer pockets by smaller Cl^−^ would lead to a compression of the mechanically bonded double cage. As for the system with one Cl^−^ and two Br^−^, the same argument would let us assign the signal with the larger CCS to the species with Cl^−^ in the middle and the two Br^−^ in the outer pocket, as this would maximize the extension of the overall structure along its long axis.

Again, using MD simulations and ^theo^CCS_N_2__ calculation, we could further confirm the assignment for the different isomers to the ion mobility signals. While the computations qualitatively match the experimentally observed size increase in the series, we were not able to completely reproduce the quantitative differences between the six species, as shown in Fig. 5b. The first outlier is [Cl,Br,Br@Pd_4_L^ethyl^_8_]^5+^ of which the average ^theo^CCS_N_2__ value is slightly too large. As the error bar indicates, and as can be more clearly seen in Fig. S35 and S36,† the double cage alternates between a more compacted and a more extended conformation. For [3Br@Pd_4_L^ethyl^_8_]^5+^ and especially for [Br,Cl,Br@Pd_4_L^ethyl^_8_]^5+^ the calculated values become too large. Furthermore, we see a strong correlation of the ^theo^CCS_N_2__ values with the Pd–Pd distance in the central cavity. Going towards the larger species we observe in the MD trajectories that the double cage not only becomes more extended but also more untwisted, thereby reducing ligand–ligand contact. Apparently, GFN2-xTB cannot perfectly reproduce the delicate balance between electrostatic interactions of the Pd cations with the halides and the London dispersion forces between the ligands. Judging from the experimental CCS values, the two larger double cages [3Br@Pd_4_L^ethyl^_8_]^5+^ and [Br,Cl,Br@Pd_4_L^ethyl^_8_]^5+^ seem to adopt more twisted and compacted structures than those observed in the MDs.

### Cage compaction through ligand–ligand π-stacking (4)

While GFN2-xTB proved to rather precisely reproduce the organic parts of the structures, we noticed that constraints at the palladium-pyridine_4_ complexes are necessary for MD simulations as the coordination sites otherwise would be too flexible and easily distort to structures far from the square-planar geometry (see the ESI† for further discussion). In particular, when performing MD simulations with GFN2-xTB on the carbazole-based monomeric cage without any constraints, we observed a π-stacking driven folding of at least two ligands each.

**Fig. 6:**
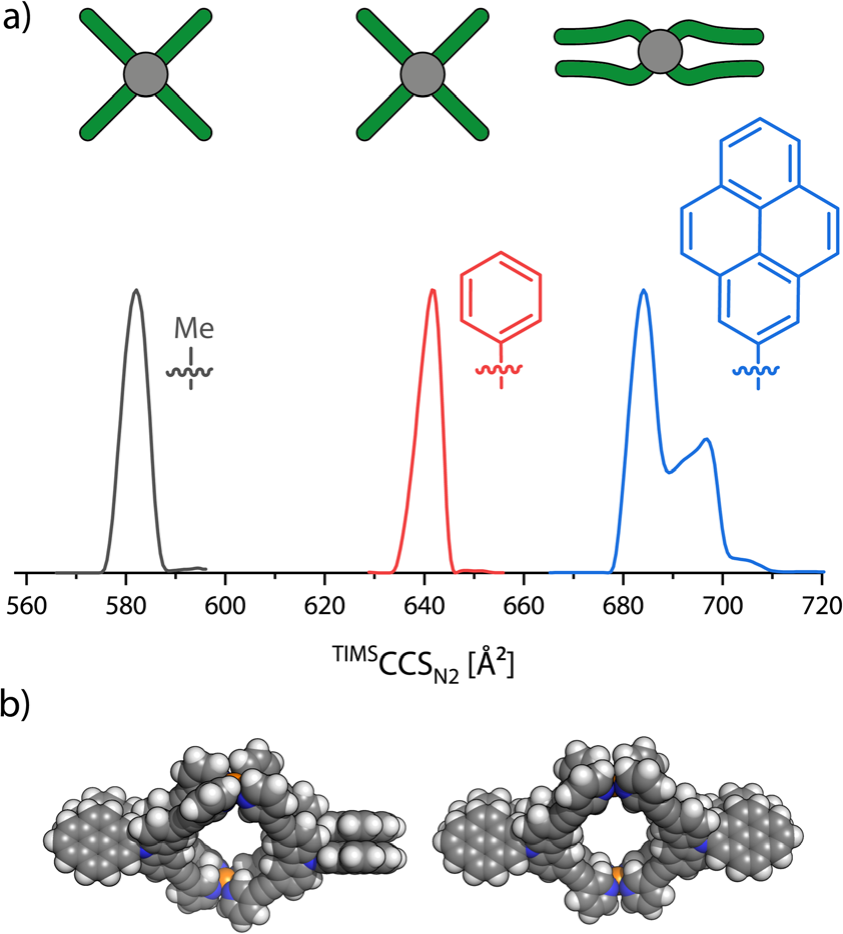
(a) Ion mobility spectra, converted to ^TIMS^CCS_N_2__ values, of [Pd_2_L^methyl^_4_]^4+^(gray), [Pd_2_L^phenyl^_4_]^4+^ (red) and [Pd_2_L^pyrenyl^_4_]^4+^ (blue) as well as a schematic top view of the three cages in the gas phase. (b) Space filling models of two proposed conformations of [Pd_2_L^pyrenyl^_4_]^4+^ in the gas-phase. Left: *D*_2_ conformer and right: *C*_S_ conformer.

In order to evaluate the match between the computed stacking behavior and experimental findings, we increased the π-surface area of neighboring ligands in order to determine how large it must become, to be experimentally detected. We thus synthesized three more carbazole based ligands: L^methyl^ carrying a methyl group attached to the central nitrogen, L^phenyl^ with a phenyl substituent and L^pyenyl^, carrying a large 2-pyrenyl group. All three ligands form a [Pd_2_L_4_]^4+^ monomeric cage as expected. These were then analyzed with TIMS-MS. Again, we only focused on the [Pd_2_L_4_]^4+^ species, as the BF_4_^−^ counter anions caused some trouble in the ^theo^CCS_N_2__ calculation (see the ESI† for further discussion). The mobilograms are shown in Fig. 6.

The increase of experimental CCS when replacing each methyl group at the outside of the cage by a phenyl group amounts to 59.5 Å^2^ (+10.2%). But when moving from phenyl to pyrenyl groups, which should lead to a larger step in size increase based on their atom count, we observed two signals at 684.1 Å^2^ and 696.9 Å^2^, amounting to an increase by only 42.6 Å^2^ (+6.6%) and 55.3 Å^2^ (+8.6%). This strongly indicates a significant compaction of the [Pd_2_L^pyrenyl^_4_]^4+^ cage as compared to the derivatives with smaller π-surface areas. DFT optimized models (using the r^2^SCAN-3c^51^ functional in ORCA 5.0.2 (ref. 52)) of the doubly folded case show that the two carbazole backbones of each pair of ligands stack parallel to each other and the two pyrenyl groups are also parallel to each other but tilted by an angle of about 50° to the carbazole units. As the doubly folded case features two pairs of ligands stacked together, the two pairs of pyrenes can either be positioned orthogonally, resulting in an overall *D*_2_ symmetry, or parallelly, resulting in a *C*_S_-symmetric structure.

For the ^theo^CCS_N_2__ calculations, as [Pd_2_L^methyl^_4_]^4+^, [Pd_2_L^phenyl^_4_]^4+^ and [Pd_2_L^pyrenyl^_4_]^4+^ (all lacking any flexible chain attachments) are rather rigid structures, we only rely on geometry optimized models of open and folded conformers, which turned out to be at least local energy minima for every cage, in this case, and did not perform further MD simulations. Additionally, we performed higher level single point calculations at the ωB97M-V^53^/def2-TZVP level as well as numerical frequency calculations with r^2^SCAN-3c. From the folded models, we also cut out fragments consisting of only one pair of ligands that are stacked together and calculated the inter-fragment dispersion using the *ab initio* method HFLD, which is achieved by a local energy decomposition at the DLPNO-CCSD level (cc-pVTZ basis, TightPNO settings).^54^ The results of these calculations are summarized in Table 1.

**Table 1:** Experimental and theoretical CCS_N_2__ values as well as relative deviations, single point energy differences Δ*E*_el_ (=*E*^folded^_el_ − *E*^open^_el_) at the ωB97M-D4/def2-TZVP level, Δ*G*_RRHO_ correction differences at the r^2^SCAN-3c level, free energy differences (Δ*G* = Δ*E*_el_ + Δ*G*_RRHO_) and inter-fragment dispersion between the ligands in the folded conformation (HFLD/cc-pVTZ/TightPNO). CCS_N_2__ values in Å^2^, energies in kJ mol^−1^

System conformation	[Pd_2_L^methyl^_4_]^4+^		[Pd_2_L^phenyl^_4_]^4+^			[Pd_2_L^pyrenyl^_4_]^4+^		
	Open	Folded	Open	Folded, *D*_2_	Folded, *C*_S_	Open	Folded, *D*_2_	Folded, *C*_S_
^TIMS^CCS_N_2__ [Å^2^]	582 ± 3		642 ± 3			684 ± 3, 697 ± 3		
^theo^CCS_N_2__ [Å^2^]	596.8	532.6	667.0	592.7	592.4	807.3	679.6	686.0
Δ_%_	+2.5%	−8.5%	+4.0%	−7.6%	−7.7%	+16.9%	−0.7%	−1.6%
Δ*E*_el_	—	+8.1	—	−7.2	−3.8	—	−88.4	−84.9
Δ*G*_RRHO_	—	+13.3	—	+16.3	+15.6	—	+26.7	+28.5
Δ*G*	—	+21.4	—	+8.9	+13.7	—	−61.7	−56.3
Inter-frag. disp.	—	−149.7	—	−168.2	—	—	−258.2	—

The ^theo^CCS_N_2__ for the doubly folded conformer of [Pd_2_L^methyl^_4_]^4+^ (obtained by geometry optimization) shows a quite large negative deviation of −8.5% to the experiment; the open form matches much better with +2.5%. A similar situation was found for [Pd_2_L^phenyl^_4_]^4+^: +4.0% for the open and −7.6% for the folded conformer with *D*_2_ symmetry and −7.7% for the folded conformer with *C*_S_ symmetry. For [Pd_2_L^pyrenyl^_4_]^4+^, however, we observe a relative deviation of +16.9% for the open model but very well matching values for the two folded conformers with deviations of only −0.7% and −1.6%. Hence, the comparison of experimental with calculated CCS values suggests that [Pd_2_L^methyl^_4_]^4+^ and [Pd_2_L^phenyl^_4_]^4+^ stay open in the gas-phase while [Pd_2_L^pyrenyl^_4_]^4+^ folds. Looking at the DFT results, we notice that the open conformer is clearly energetically favored for [Pd_2_L^methyl^_4_]^4+^. This is both driven by the electronic energy (Δ*E*_ωB97M_ = +135.1 kJ mol^−1^), due to the avoidance of strain that would not be compensated for by π-stacking gains, and the Δ*G*_RRHO_ term (+13.3 kJ mol^−1^), due to the loss of vibrational degrees of freedom upon folding. The attractive D4 dispersion correction^42^ cannot compensate for the energy increase caused by the distortion (Δ*G*_D4_ = −127.0 kJ mol^−1^). For [Pd_2_L^phenyl^_4_]^4+^, Δ*G*_RRHO_ is not much larger and Δ*E*_el_ is not significantly lower, leading to a total Δ*G* of +8.9 kJ mol^−1^ for the *D*_2_ symmetric conformer and +13.7 kJ mol^−1^ for the *C*_S_ symmetric one, respectively. When going from the phenyl to the pyrenyl substituted cage, Δ*E*_el_ decreases drastically, dominated by very strong π–π interactions between the ligands. For the two stacked conformers, Δ*G*_RRHO_ increases to around +26.7 kJ mol^−1^ and +28.5 kJ mol^−1^ but cannot nearly compensate for the dispersive forces causing free energies of −61.7 kJ mol^−1^ and −56.3 kJ mol^−1^, respectively. The computed inter-fragment dispersion between the pair of folded ligands increases from methyl to phenyl by +12.4% and from phenyl to pyrenyl by +53.5%, also showing the significantly stronger interaction in the latter cage. A more elaborate overview of the calculated energies is found in the ESI, Section 2.4.†

The combined results from the ^theo^CCS_N_2__ calculations and the quantum mechanical calculations give strong indications that [Pd_2_L^methyl^_4_]^4+^ and [Pd_2_L^phenyl^_4_]^4+^ stay open and [Pd_2_L^pyrenyl^_4_]^4+^ folds in the gas-phase. Alternatively, if the VV10 dispersion correction method^55^ is used (“ωB97M-V”), the case for [Pd_2_L^phenyl^_4_]^4+^ is less clear. Here, Δ*G* values of −3.5 kJ mol^−1^ and +2.0 kJ mol^−1^ for the *D*_2_ and *C*_S_ conformers are obtained, suggesting that both the open and folded conformations could be populated. The ^theo^CCS_N_2__ for a tentative singly folded conformer, in which only one pair of ligands folds together, was calculated to be 637.8 Å^2^ with a relative difference of only −0.6% from the experimental value. However, the inaccuracies of the trajectory method, the dispersion correction and the Δ*G*_RRHO_ calculation do not allow more than speculations in this regard (corresponding ^theo^CCS_N_2__ values and DFT energies are found in the ESI†).

A crystal structure of the pyrenyl carbazole cage was obtained by slow diffusion of isopropyl ether into a 0.2 mM solution of [Pd_2_L^pyrenyl^_4_](BF_4_)_4_ in MeNO_2_ (see the ESI† for details). The asymmetric unit contains two cages, surprisingly – at least on first sight – both in an open conformation. The cages adopt slightly twisted helical conformations (one left- and one right-handed) in accordance with the DFT-optimized models. In this regard, the here found structure differs from the rather orthogonally positioned ligands in the crystal structure of [Pd_2_L^hexyl^_4_]^4+^, as reported by us before.^43^ The apparent disagreement of the folded state, as found in the gas-phase (and backed up by the computations), and the open conformer in the crystal can be resolved when examining the packing of the cages in the solid state, revealing extensive inter-cage π-stacking, probably rendering intra-cage folding redundant.

## Conclusions

Trapped ion mobility spectrometry in combination with collisional cross section calculations is a powerful approach for gaining detailed insights into the gas-phase structures of large assemblies such as Pd-based coordination cages. On the basis of a common carbazole-based cage motif, we here realized four substantially different test settings to compare experimental ion mobilities with theoretically modeled values. In particular, we show that alkyl chains start folding back from hexyl onwards and while they retrench deep into the crevices of a wide-meshed [Pd_2_L_4_]^4+^cage (1), they cover the more closed surface of corresponding dimeric [3Br@Pd_4_L^R^_8_]^5+^ assemblies (2). We further show that even small size changes between mechanically bonded dimers, containing different combinations of halide guests, can be resolved by TIMS and – at least qualitatively – reproduced using CCS calculations (3). Finally, we demonstrate how differences in inter-ligand π-stacking, leading to a switch between open and folded conformations, can be revealed by IMS and interpreted by theoretical modeling.

With ion mobility mass spectrometry beginning to find more widespread application in supramolecular chemistry, proper interpretation of obtained mobility data in tandem with the preparation of reasonable molecular models will gain crucial importance. Owing to the high precision and resolution of modern IMS instruments, this method offers much more than only delivering rough smaller/larger answers to chemical problems such as guest localization in non-covalent assemblies or the structuring of soft chain decorations around more rigid core structures. Way more detailed structural information can be extracted from IMS data when combined with modern modeling techniques. Fortunately, computational workflows combining conformational sampling – usually MD-based – with refining geometry optimizations, followed by Boltzmann-weighted identification of plausible gas-phase structures and their conversion into averaged CCS values have become more accessible, even to labs with a focus on synthesis and analytics. In this respect, our study not only showcases the power of this technique to analyze common structural features such as chain attachments, rigid *vs.* soft behavior of assemblies and guest-modulated size changes of a host structure. It further contributes a valuable methodology to the toolbox of the growing number of supramolecular chemists with access to ion mobility data for gas-phase compound characterization. While experimentally determining and theoretically modeling the gas-phase dimensions of flexible and chain-decorated assemblies has significance on its own from a fundamental point of view, the herein presented workflow is also of high value for cage-based systems and applications that mainly operate in solution. In particular, mass spectrometric methods belong to the standard repertoire of techniques for the characterization of soluble host–guest complexes and the addition of ion mobility data can deliver decisive information about guest localization (engulfed, protruding or outside) and guest-modulated changes of the host structure (expanded or contracted) that may be difficult to elucidate by solution methods, such as NMR, alone. Ion mobility mass spectrometry can also complement solution-based spectroscopic methods in the mechanistic evaluation of catalytic reactions, *e.g.* by detecting non-covalent complexes of reactive intermediates. In recent years, self-assemblies have become structurally more and more complex (*e.g.* heteroleptic cages formed by non-statistical multicomponent assembly^9^). Demands for powerful analytical techniques are multiplied when coexisting species have to be distinguished in stimuli-responsive systems.^7^ The extra dimension of high-resolution ion mobility can here solve problems of structural assignment that are out of reach for solution techniques such as DOSY NMR. Furthermore, the action of different solvents on the assembly outcome (*e.g.* selection between isomeric structures) can be studied by ion mobility mass spectrometry, where gas-phase results on the assemblies' size and shape allow conclusions to be drawn about the solution behaviour and can be directly compared to characterization methods in solution.

## Author contributions

C. D. and G. H. C. conceived the idea. C. D. conducted most of the synthesis, characterization, analysis and calculations and wrote the manuscript. A. B. acquired and analysed the IM-MS data. A. P. acquired and analysed the DOSY data. M. A. supported C. D. with the ligand synthesis. J. J. H. solved and refined the X-ray structures. C. J. S. gave advice for the calculations. G. H. C. managed the project, aided in experimental design, and revised the manuscript. All authors provided comments and approved the final version of the manuscript.

## Conflicts of interest

The authors declare no competing financial interest.

## Supplementary Material

SC-015-D4SC04786A-s001

SC-015-D4SC04786A-s002

## Data Availability

Data for this article, including NMR spectra, mass and ion mobility spectra, ORCA output files, model structures, crystallographic data, and calculated CCS values are available at the RESOLVdata repository (https://data.tu-dortmund.de/dataverse/resolv) at https://doi.org/10.17877/RESOLV-2024-lyfszm2x.
