# Supplementary material for: London dispersion driven compaction of coordination cages in the gas-phase – a combined ion mobility and theoretical study
Source: Chem Sci. 2024 Oct 14;15(46):19264–72. doi: 10.1039/d4sc04786a (PMC11520353; doi:10.1039/d4sc04786a)
Supplement: SC-015-D4SC04786A-s001 [file SC-015-D4SC04786A-s001.pdf]

Supplementary Information (SI) for Chemical Science.
This journal is © The Royal Society of Chemistry 2024

# London Dispersion driven Compaction of Coordination Cages in the Gas Phase – A combined Ion Mobility and Theoretical Study

## Supporting Information

Christoph Drechsler, Ananya Baksi, André Platzek, Mert Acar, Julian J. Holstein, Christopher J. Stein, Guido H. Clever

**1. Experimental section** 2

*1.1 Materials and Methods* 2

*1.2 Synthesis and characterization of $Pd_2L^R{}_4$ and $3X@Pd_4L^R{}_8$* 3
1.2.1 Synthesis of $L^R$ 3
1.2.2 Formation of cages $[Pd_2L^R{}_4]^{4+}$ 6
1.2.3 ESI MS and TIMS spectra of $[Pd_2L^R{}_4]^{4+}$ 8
1.2.4 Cage formation of $[3X@Pd_4L^R{}_8]^{5+}$ 9
1.2.5 ESI MS and TIMS spectra of $[3X@Pd_4L^R{}_8]^{5+}$ 11
1.2.6 DOSY NMR spectra of $Pd_2L^R{}_4$ and $3Br@Pd_4L^R{}_8$ 12

*1.3 Synthesis and characterization of $[Pd_2L^{methyl}{}_4]^{4+}$* 15
1.3.1 Synthesis of $L^{methyl}$ 15
1.3.2 Cage formation of $[Pd_2L^{methyl}{}_4]^{4+}$ 16
1.3.3 ESI MS and TIMS spectra of $[Pd_2L^{methyl}{}_4]^{4+}$ 17

*1.4 Synthesis and characterization of $[Pd_2L^{phenyl}{}_4]^{4+}$* 18
1.4.1 Synthesis of $L^{phenyl}$ 18
1.4.2 Cage formation of $[Pd_2L^{phenyl}{}_4]^{4+}$ 19
1.4.3 ESI MS and TIMS spectra of $[Pd_2L^{phenyl}{}_4]^{4+}$ 20

*1.5 Synthesis and characterization of $[Pd_2L^{pyrenyl}{}_4]^{4+}$* 21
1.5.1 Synthesis of $L^{pyrenyl}$ 21
1.5.2 Cage formation of $[Pd_2L^{pyrenyl}{}_4]^{4+}$ 23
1.5.3 ESI MS and TIMS spectra of $[Pd_2L^{pyrenyl}{}_4]^{4+}$ 25

*1.6 Single crystal X-ray diffraction analysis* 26

**2. Computational section** 30

*2.1 Molecular Dynamics Simulations with GFN2-xTB* 30

*2.2 $^{theo}CCS_{N2}$ calculation* 30
2.2.1 Collidoscope, general settings 30
2.2.2 HPCCS, general settings 30
2.2.3 IMoS, general settings 31
2.2.4 Optimization of Fluorine Lennard-Jones parameters for Collidoscope 31
2.2.5 $^{theo}CCS_{N2}$ calculation using different Trajectory Method software 33
2.2.6 $^{theo}CCS_{N2}$ calculation of snapshots from the trajectories 33
2.2.7 $^{theo}CCS_{N2}$ of geometry optimized cage systems and according experimental values 40

*2.3 Geometry optimizations, numerical frequencies and single point energies* 40
2.3.1 Headers 40
2.3.2 Optimized models 42

*2.4 Additional QM calculations* 43
2.4.1 Energy calculations comparing D4 and VV10 and considering the single-folded conformer 43
2.4.2 Geometry optimizations using different DFT methods, angle scans and CSD-searches 44

# 1. Experimental section

## 1.1 Materials and Methods

Chemicals and standard solvents were purchased from Sigma Aldrich, Acros Organics, Carl Roth, TCI Europe, VWR, abcr or other suppliers and used as received, if not mentioned otherwise. Deuterated solvents were purchased from Eurisotop. For synthesis, HPLC grade solvents and for purification technical solvents were used if not mentioned otherwise. Automated flash chromatography was conducted using a Biotage® Isolera One™ and Biotage® Sfär Silica D Duo 60 µm cartridges. Recycling gel permeation chromatography was performed on Japan Analytical Industry NEXT and LaboACE instruments using JAIGEL 1-HH and 2-HH columns, 20 mm x 600 mm, with HPLC grade chloroform and an average flowrate of 7 mL/min.

NMR spectroscopic data was measured on the spectrometers Bruker AV 400 Avance III HD NanoBay (400 MHz), Bruker AV 500 Avance NEO (500 MHz), Agilent Technologies DD2 (500 MHz), Bruker AV 500 Avance III HD (500 MHz), AV 500 Avance III HD (500 MHz), Bruker AV 600 Avance III HD (600 MHz), Bruker Avance NEO 600 (600 MHz) and Bruker AV 700 Avance III HD (700 MHz). For 1H NMR and 13C NMR spectra signals were referenced to the residual solvent peak ($^1$H: acetonitrile: 1.94 ppm, DMSO: 2.54 ppm, chloroform: 7.26 ppm; $^{13}$C: acetonitrile: 118.26 ppm, DMSO: 39.52 ppm, chloroform: 77.16 ppm). $^1$H DOSY NMR spectra were recorded with a *dstebpgp3s* pulse sequence with diffusion delays D20 of 0.08 s and gradient powers P30 of 2500 to 3000 µs.[1,2] T1 analyses of the corresponding signals in the 1D spectra were performed to obtain the diffusion coefficients $D$ using the STEJSKAL-TANNER-equation.[3,4] Hydrodynamic radii $r_H$ were calculated using the STOKES-EINSTEIN-equation.[5]

For details on the single crystal X-ray diffraction analysis see Section 1.6.

Mass spectra and trapped ion mobility spectra were recorded with Bruker ESI timsTOF spectrometer (positive mode). For the calibration, Agilent™ ESI Low Concentration Tuning Mix was used. About 25 µL of each sample in $CD_3CN$ was diluted 1:10 and electrosprayed at 3 µL/min flow rate. Minimum electrical potentials were optimized for each sample to avoid fragmentation. Typically, the following instrumental parameters were used: Capillary voltage: 2500 V, End plate offset: 200 V, Dry temperature: 75 °C, Funnel 1 RF: 200V, Funnel 2 RF: 200 V, Multiple RF: 200 V, Deflection delta: 60 V, Quadrupole ion energy: 3 V, Collision energy: 3 V, Transfer time: 70 µs, Prepulse storage: 15 µs. The measured inverse mobility was then converted to the experimental collision cross section ($^{TIMS}CCS_{N2}$) using the Mason-Schamp equation.[6,7] Calibration was achieved using the Agilent tune mix with $^{DT}CCS_{N2}$-values published by Stow *et. al.*[8] The Agilent Tune Mix contains 13 different analytes of fluorinated hydrogen chains with known CCS values. In the first step, the masses and mobilities of the calibrant were measured and the resulting file was calibrated internally. The samples were then measured and an external calibration with the previous recorded calibration data set was carried out to determine the CCS values of the samples. It is important to mention that the calibration file was newly recorded before every measurement series to avoid measurement fluctuations caused by the device. The measurement conditions for the calibrant and the sample were also kept constant.Each sample was measured in low (survey) and high (custom) resolution mode. A pressure difference of 1.7 mbar between the entrance and the exit of the TIMS tunnel was used. For high resolution measurements a 0.06 Vs/cm2 difference and 450 ms ramp time was used and the achieved resolution was >200.

For all computational studies the high-performance cluster of TU Dortmund University called LiDO3 was used.

## Data availability

Data for this article, including NMR spectra, mass and ion mobility spectra, ORCA output files, model structures, crystallographic data, calculated CCS values are available at the RESOLVdata repository (https://data.tu-dortmund.de/dataverse/resolv)
at https://doi.org/10.17877/RESOLV-2024-lyfszm2x

## 1.2 Synthesis and characterization of $Pd_2L^R{}_4$ and 3X@$Pd_4L^R{}_8$

### 1.2.1 Synthesis of $L^R$

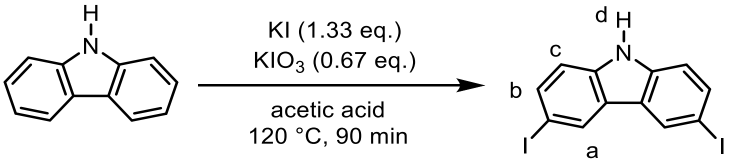


**Scheme S1.** Synthesis of 3,6-Diiodocarbazole.

To a suspension of carbazole (1 eq., 5 mmol, 836 mg) and KI (1.33 eq., 6.65 mmol, 1104 mg) in 50 mL acetic acid was given $KIO_3$ (0.67 eq., 3.35 mmol, 717 mg) and the mixture was stirred at 120 °C for 90 min. The reaction mixture was then first allowed to cool down and subsequently taken up in a water/ethyl acetate mixture. The organic phase was washed with water three times and then the solvent was removed *in vacuo*. The remaining solid was then purified using automated flash chromatography (*n*-pentane/EtOAc, 0 % to 25 %). 3,6-Diiodocarbazole was obtained as white solid (61 %, 3.03 mmol 1268 mg).

**$^1$H NMR (600 MHz, 298 K, $CDCl_3$) of 3,6-diiodocarbazole:** $\delta$ [ppm] = 8.33 (s, 2H, a), 8.08 (br, 1H, d), 7.68 (d, $J$ = 8.4 Hz, 2H, b), 7.21 (d, $J$ = 8.5 Hz, 2H, c).

**$^{13}$C{$^1$H} NMR (151 MHz, 298 K, $CDCl_3$) of 3,6-diiodocarbazole:** $\delta$ [ppm] = 138.68, 134.97, 129.59, 124.74, 112.88, 82.60.

**ESI MS of 3,6-diiodocarbazole (neg. mode):** measured: 417.8568, calculated for $[C_{12}H_7NI_2–H]^-$: 417.8595.

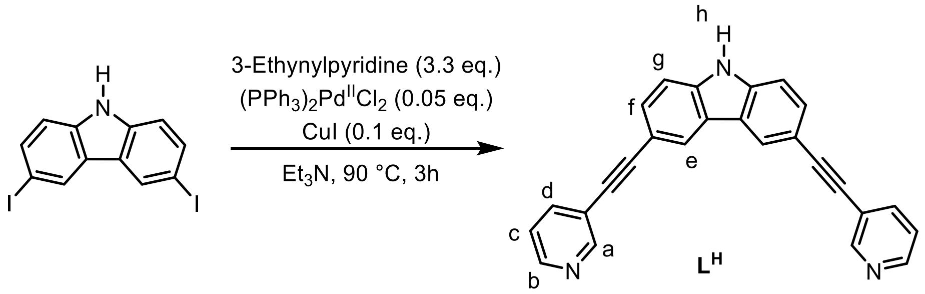


**Scheme S2.** Synthesis of $L^H$.

For the synthesis of $L^H$, 3,6-diiodocarbazole (1.0 eq., 1.19 mmol, 500 mg) and 3-ethynylpyridine (3.3 eq., 3.93 mmol, 405 mg) were dissolved in 12 mL $NEt_3$ and degassed with three *freeze-pump-thaw*-cycles. After the addition of $(PPh_3)_2Pd(II)Cl_2$ (0.05 eq., 0.119 mmol, 83.5 mg) and CuI (0.1 eq., 0.238 mmol, 45.3 mg) three more *freeze-pump-thaw*-cycles were conducted. The reaction mixture was then heated and stirred at 90 °C for 3 h. As $L^H$ is insoluble in MeCN, the mixture was filtered and the remaining solid was washed with MeCN yielding $L^H$ as yellow powder (85 %, 1.01 mmol, 374 mg).

**$^1$H NMR (500 MHz, 298 K, DMSO-d$_6$) of L$^H$:** $\delta$ [ppm] = 11.90 (s, 1H, h), 8.90 (br, a), 8.71 (br, b), 8.55 (s, 2H, e), 8.04 (d, $J$ = 7.9 Hz, 2H, d), 7.69 (d, $J$ = 8.5 Hz, 2H, f), 7.61 (d, $J$ = 8.4 Hz, 2H, g), 7.54 (br, c).

**$^{13}$C{$^1$H} NMR (126 MHz, 298 K, DMSO-d$_6$) of L$^H$:** $\delta$ [ppm] = 140.21, 138.25, 129,67, 124.43, 122.13, 112.05, 111.82, 94.09. Of 13 expected signals only 8 are observed, probably due to low signal intensity because of poor solubility of **L$^H$**.

**ESI MS of L$^H$:** measured: 370.1348, calculated for $[C_{26}H_{15}N_3+H]^+$: 370.1339.

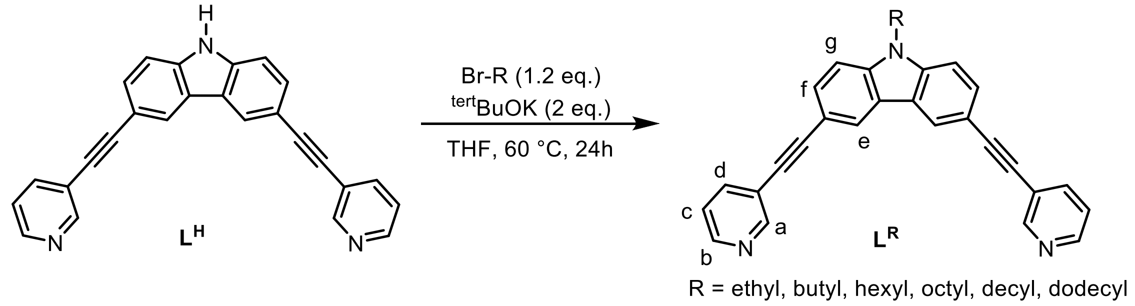


**Scheme S3.** Synthesis of **L$^R$**.

For each of the final ligands **L$^R$** (1.0 eq., 81.2 µmol, 30 mg), $^{tert}$BuOK (2.0 eq., 162 µmol, 18.2 mg) and 1-bromoalkane (1.2 eq., 97.44 µmol) was dissolved in 3 mL THF and heated and stirred at 60 °C for 24 h. Afterwards, the reaction mixture was transferred into a round bottom flask, silica was added and the solvent was removed *in vacuo*. The resulting ligands were obtained after automated flash chromatography (*n*-pentane/ethyl acetate in varying ratios) as yellow-white solids (41–99 %).

**$^1$H NMR (400 MHz, 298 K, CD$_3$CN) of L$^{ethyl}$:** $\delta$ [ppm] = 8.78 (s, 2H, a), 8.55 (d, $J$ = 4.9 Hz, 2H, b), 8.39 (s, 2H, e), 7.91 (d, $J$ = 7.9 Hz, 2H, d), 7.72 (d, $J$ = 8.5 Hz, 2H, f), 7.61 (d, $J$ = 8.5 Hz, 2H, g), 7.39 (dd, $J_1$ = 7.9 Hz, $J_2$ = 4.8 Hz, 2H, c), 4.46 (q, $J$ = 7.2 Hz, 2H, N-CH$_2$-), 1.41 (t, $J$ = 7.2 Hz, 3H, -CH$_3$).

**$^{13}$C{$^1$H} NMR (126 MHz, 298 K, DMSO-d$_6$) of L$^{ethyl}$:** $\delta$ [ppm] = 151.62, 148.66, 140.12, 138.38, 129.85, 124.62, 123.75, 122.01, 120.09, 112.43, 110.16, 93.88, 84.65, 17.28, 14.10.

**ESI MS of L$^{ethyl}$:** measured: 398.1658, calculated for $[C_{28}H_{19}N_3+H]^+$: 397.1573.

**$^1$H NMR (400 MHz, 298 K, CD$_3$CN) of L$^{butyl}$:** $\delta$ [ppm] = 8.77 (s, 2H, a), 8.55 (d, $J$ = 4.9 Hz, 2H, b), 8.39 (s, 2H, e), 7.91 (d, $J$ = 7.9 Hz, 2H, d), 7.70 (d, $J$ = 8.5 Hz, 2H, f), 7.60 (d, $J$ = 8.5, 2H, g), 7.39 (dd, $J_1$ = 7.9 Hz, $J_2$ = 4.8 Hz, 2H, c), 4.41 (q, $J$ = 7.2 Hz, 2H, N-CH$_2$-), 1.85 (m, 2H, N-CH$_2$-C*H*$_2$-), 1.38 (m, 2H, -CH$_2$-), 0.94 (t, $J$ = 7.2 Hz, 3H, -CH$_3$).

**$^{13}$C{$^1$H} NMR (126 MHz, 298 K, DMSO-d$_6$) of L$^{butyl}$:** $\delta$ [ppm] = 151.5, 148.7, 140.6, 138.4, 129.9, 124.6, 123.8, 121.9, 120.0, 112.4, 110.4, 94.1, 84.7, 42.5, 30.7, 19.7, 13.8.

**ESI MS of L$^{butyl}$:** measured: 425.1925, calculated for $[C_{30}H_{22}N_3+H]^+$: 425.1886.

**$^1$H NMR (500 MHz, 298 K, CD$_3$CN) of L$^{hexyl}$:** $\delta$ [ppm] = 8.77 (s, 2H, a), 8.55 (d, $J$ = 4.9 Hz, 2H, b), 8.39 (s, 2H, e), 7.91 (d, $J$ = 7.9 Hz, 2H, d), 7.70 (d, $J$ = 8.5, 2H, f), 7.60 (d, $J$ = 8.5, 2H, g), 7.39 (dd, $J_1$ = 7.9 Hz, $J_2$ = 4.8 Hz, 2H, c), 4.40 (q, $J$ = 7.2 Hz, 2H, N-CH$_2$-), 1.87 (m, 2H, N-CH$_2$-C*H*$_2$-), 1.21-1.40 (m, 6H, -CH$_2$-), 0.85 (t, $J$ = 7.2 Hz, 3H, -CH$_3$).

**$^{13}$C{$^1$H} NMR (126 MHz, 298 K, DMSO-d$_6$) of L$^{hexyl}$:** $\delta$ [ppm] = 151.4, 148.7, 140.6, 138.3, 129.8, 124.5, 123.7, 121.8, 120.0, 112.3, 110.3, 93.9, 84.7, 42.9, 30.9, 28.5, 26.1, 22.0, 13.8.

**ESI MS of L$^{hexyl}$:** measured: 454.2348, calculated for $[C_{32}H_{27}N_3+H]^+$: 454.2278.

**$^{1}H$ NMR (600 MHz, 298 K, $CD_3CN$) of $L^{octyl}$:** $\delta$ [ppm] = 8.77 (s, 2H, a), 8.55 (d, $J$ = 4.9 Hz, 2H, b), 8.39 (s, 2H, e), 7.91 (d, $J$ = 7.9 Hz, 2H, d), 7.70 (d, $J$ = 8.5 Hz, 2H, f), 7.60 (d, $J$ = 8.5 Hz, 2H, g), 7.39 (dd, $J_1$ = 7.9 Hz, $J_2$ = 4.8 Hz, 2H, c), 4.40 (q, $J$ = 7.2 Hz, 2H, N-$CH_2$-), 1.86 (m, 2H, N-$CH_2$-C$H_2$-), 1.16-1.40 (m, 10H, -$CH_2$-), 0.85 (t, $J$ = 7.2 Hz, 3H, -$CH_3$).

**$^{13}C\{^{1}H\}$ NMR (126 MHz, 298 K, DMSO-$d_6$) of $L^{octyl}$:** $\delta$ [ppm] = 151.4, 148.7, 140.5, 138.3, 129.8, 124.5, 123.7, 121.8, 120.0, 112.3, 110.3, 93.9, 84.7, 42.6, 31.1, 28.7, 28.6, 28.5, 26.4, 22.0, 13.9.

**ESI MS of $L^{octyl}$:** measured: 482.2650, calculated for $[C_{34}H_{31}N_3+H]^+$: 482.2591.

**$^{1}H$ NMR (400 MHz, 298 K, $CD_3CN$) of $L^{decyl}$:** $\delta$ [ppm] = 8.77 (s, 2H, a), 8.55 (d, $J$ = 4.9 Hz, 2H, b), 8.39 (s, 2H, e), 7.91 (d, $J$ = 7.9 Hz, 2H, d), 7.70 (d, $J$ = 8.5 Hz, 2H, f), 7.60 (d, $J$ = 8.5 Hz, 2H, g), 7.39 (dd, $J_1$ = 7.9 Hz, $J_2$ = 4.8 Hz, 2H, c), 4.40 (q, $J$ = 7.2 Hz, 2H, N-$CH_2$-), 1.86 (m, 2H, N-$CH_2$-C$H_2$-), 1.16-1.40 (m, 14H, -$CH_2$-), 0.87 (t, J = 7.2, 3H, -$CH_3$).

**$^{13}C\{^{1}H\}$ NMR (126 MHz, 298 K, DMSO-$d_6$) of $L^{decyl}$:** $\delta$ [ppm] = 151.43, 148.64, 140.47, 138.31, 129.77, 124.51, 123.73, 121.82, 112.28, 110.31, 93.90, 84.62, 31.24, 28.90, 28.81, 28.67, 28.62, 28.46, 26.34, 22.06, 13.94.

**ESI MS of $L^{decyl}$:** measured: 510.2966, calculated for $[C_{36}H_{35}N_3+H]^+$: 510.2904.

**$^{1}H$ NMR (500 MHz, 298 K, $CD_3CN$) of $L^{dodecyl}$:** $\delta$ [ppm] = 8.77 (s, 2H, a), 8.55 (d, $J$ = 4.9 Hz, 2H, b), 8.39 (s, 2H, e), 7.91 (d, $J$ = 7.9 Hz, 2H, d), 7.70 (d, $J$ = 8.5, 2H, f), 7.60 (d, $J$ = 8.5 Hz, 2H, g), 7.39 (dd, $J_1$ = 7.9 Hz, $J_2$ = 4.8 Hz, 2H, c), 4.40 (q, $J$ = 7.2 Hz, 2H, h), 1.86 (m, 2H, i), 1.16-1.38 (m, 18H, j), 0.87 (t, $J$ = 7.2 Hz, 3H, k).

**$^{13}C\{^{1}H\}$ NMR (126 MHz, 298 K, $CDCl_3$) of $L^{dodecyl}$:** $\delta$ [ppm] = 152.26, 148.20, 140.94, 138.43, 129.97, 124.52, 123.23, 122.62, 121.15, 113.37, 109.31, 94.02, 84.69, 43.64, 32.06, 29.72, 29.67, 29.62, 29.48, 29.47, 29.09, 27.39, 22.83, 14.29. Of 25 expected signals only 24 could be detected; probably due to signal overlap in the region around 29.5 ppm.

**ESI MS of $L^{dodecyl}$:** measured: 538.3271, calculated for $[C_{38}H_{39}N_3+H]^+$: 538.3217.

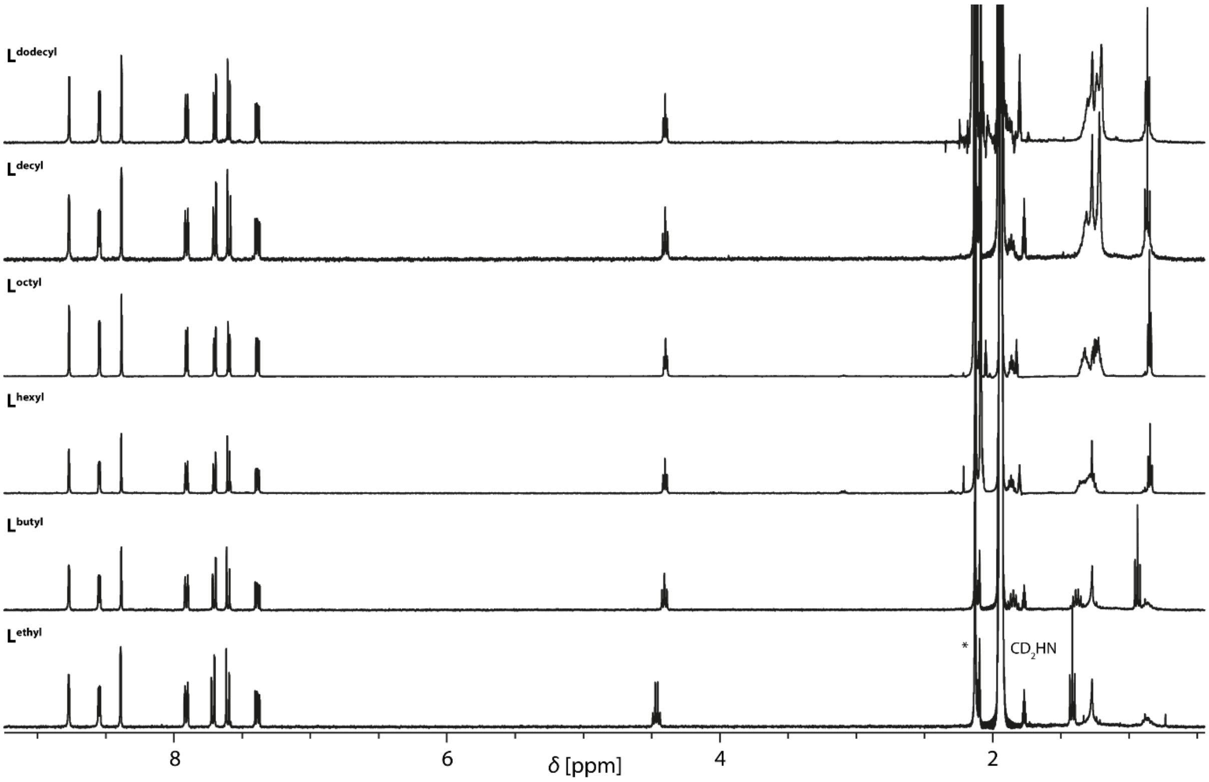


**Figure S1.** $^1$H NMR spectra (298 K, $CD_3CN$) of **L$^R$**.

## 1.2.2 Formation of cages $[Pd_2L^R_4]^{4+}$

294 µL of a 3 mM suspension of **L$^R$** in $CD_3CN$ (1.0 eq.) were combined with 29.4 µL of a 15 mM stock solution of $[Pd(CH_3CN)_4](BF_4)_2$ in $CD_3CN$ (0.5 eq.) and 227 µL $CD_3CN$ yielding 550 µL of a 0.4 mM solution of $[Pd_2L^R_4](BF_4)_4$.

**$^1$H NMR (400 MHz, 298 K, $CD_3CN$) of** $[Pd_2L^{ethyl}_4]^{4+}$**:** $\delta$ [ppm] = 9.00 (s, 8H, a), 8.77 (d, $J$ = 5.9 Hz, 8H, b), 8.49 (s, 8H, e), 8.17 (d, $J$ = 8.2 Hz, 8H, d), 7.73 (d, $J$ = 8.5 Hz, 8H, f), 7.62 (dd, $J_1$ = 8.2 Hz, $J_2$ = 5.9 Hz, 8H, c), 7.59 (d, $J$ = 8.6 Hz, 8H, g), 4.40 (q, $J$ = 7.1 Hz, 8H, N-$CH_2$-), 1.34 (t, $J$ = 7.2 Hz, 12H, -$CH_3$).

**$^1$H NMR (400 MHz, 298 K, $CD_3CN$) of** $[Pd_2L^{butyl}_4]^{4+}$**:** $\delta$ [ppm] = 9.00 (s, 8H, a), 8.77 (d, $J$ = 5.9 Hz, 8H, b), 8.48 (s, 8H, e), 8.17 (d, $J$ = 8.2 Hz, 8H, d), 7.71 (d, $J$ = 8.5 Hz, 8H, f), 7.62 (dd, $J_1$ = 8.2 Hz, $J_2$ = 5.9 Hz, 8H, c), 7.58 (d, $J$ = 8.6 Hz, 8H, g), 4.34 (t, $J$ = 7.1 Hz, 8H, N-$CH_2$-), 1.77 (m, 8H, N-$CH_2$-C$H_2$-), 1.30 (m, 8H, -$CH_2$-), 0.86 (t, $J$ = 7.5 Hz, 12H, -$CH_3$).

**$^1$H NMR (500 MHz, 298 K, $CD_3CN$) of** $[Pd_2L^{hexyl}_4]^{4+}$**:** $\delta$ [ppm] = 8.99 (s, 8H, a), 8.77 (d, $J$ = 5.8 Hz, 8H, b), 8.45 (s, 8H, e), 8.17 (d, $J$ = 8.1 Hz, 8H, d), 7.71 (d, $J$ = 8.7 Hz, 8H, f), 7.62 (dd, $J_1$ = 8.2 Hz, $J_2$ = 5.9 Hz, 8H, c), 7.58 (d, $J$ = 8.3 Hz, 8H, g), 4.34 (t, $J$ = 7.1 Hz, 8H, N-$CH_2$-), 1.78 (m, 8H, N-$CH_2$-C$H_2$-), 1.13-1.33 (m, 24H, -$CH_2$-), 0.76 (t, $J$ = 7.5 Hz, 12H, -$CH_3$).

**$^1$H NMR (400 MHz, 298 K, $CD_3CN$) of** $[Pd_2L^{octyl}_4]^{4+}$**:** $\delta$ [ppm] = 9.00 (s, 8H, a), 8.77 (d, $J$ = 5.8 Hz, 8H, b), 8.45 (s, 8H, e), 8.17 (d, $J$ = 8.1 Hz, 8H, d), 7.71 (d, $J$ = 8.7 Hz, 8H, f), 7.62 (dd, $J_1$ = 8.2 Hz, $J_2$ = 5.9 Hz, 8H, c), 7.57 (d, $J$ = 8.3 Hz, 8H, g), 4.34 (t, $J$ = 7.1 Hz, 8H, N-$CH_2$-), 1.78 (m, 8H, N-$CH_2$-C$H_2$-), 1.06-1.34 (m, 40H, -$CH_2$-), 0.77 (t, $J$ = 7.5 Hz, 12H, -$CH_3$).

**$^1$H NMR (400 MHz, 298 K, $CD_3CN$) of** $[Pd_2L^{decyl}_4]^{4+}$**:** $\delta$ [ppm] = 8.99 (s, 8H, a), 8.77 (d, $J$ = 5.8 Hz, 8H, b), 8.47 (s, 8H, e), 8.17 (d, $J$ = 8.1 Hz, 8H, d), 7.71 (d, $J$ = 8.7 Hz, 8H, f), 7.62 (dd, $J_1$ = 8.2 Hz, $J_2$ = 5.9 Hz, 8H, c), 7.57 (d, $J$ = 8.3 Hz, 8H, g), 4.34 (t, $J$ = 7.1 Hz, 8H, N-$CH_2$-), 1.78 (m, 8H, N-$CH_2$-C$H_2$-), 1.06-1.34 (m, 56H, -$CH_2$-), 0.79 (t, $J$ = 7.5 Hz, 12H, -$CH_3$).

**$^1$H NMR (400 MHz, 298 K, $CD_3CN$) of** [$Pd_2$**L**$^{dodecyl}$$_4$]$^{4+}$**:** *δ* [ppm] = 9.00 (s, 8H, a), 8.78 (d, *J* = 5.8 Hz, 8H, b), 8.48 (s, 8H, e), 8.17 (d, *J* = 8.1 Hz, 8H, d), 7.71 (d, *J* = 8.7 Hz, 8H, f), 7.62 (dd, $J_1$ = 8.2 Hz, $J_2$ = 5.9 Hz, 8H, c), 7.57 (d, *J* = 8.3 Hz, 8H, g), 4.34 (t, *J* = 7.1 Hz, 8H, N-$CH_2$-), 1.78 (m, 8H, N-$CH_2$-C$H_2$-), 1.00-1.34 (m, 72H, -$CH_2$-), 0.80 (t, *J* = 7.5 Hz, 12H, -$CH_3$).

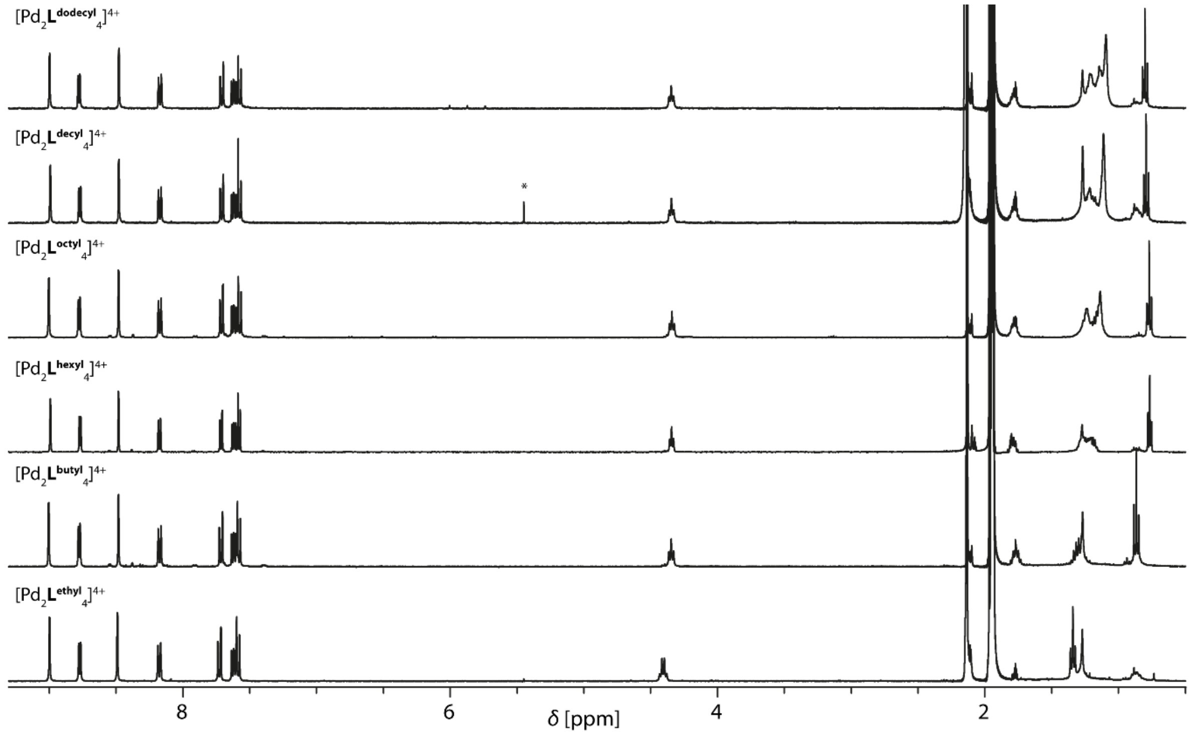


**Figure S2.** $^1$H NMR spectra (298 K, $CD_3CN$) of [$Pd_2$**L**$^{R}$$_4$]$^{4+}$.

### 1.2.3 ESI MS and TIMS spectra of $[Pd_2L^R_4]^{4+}$

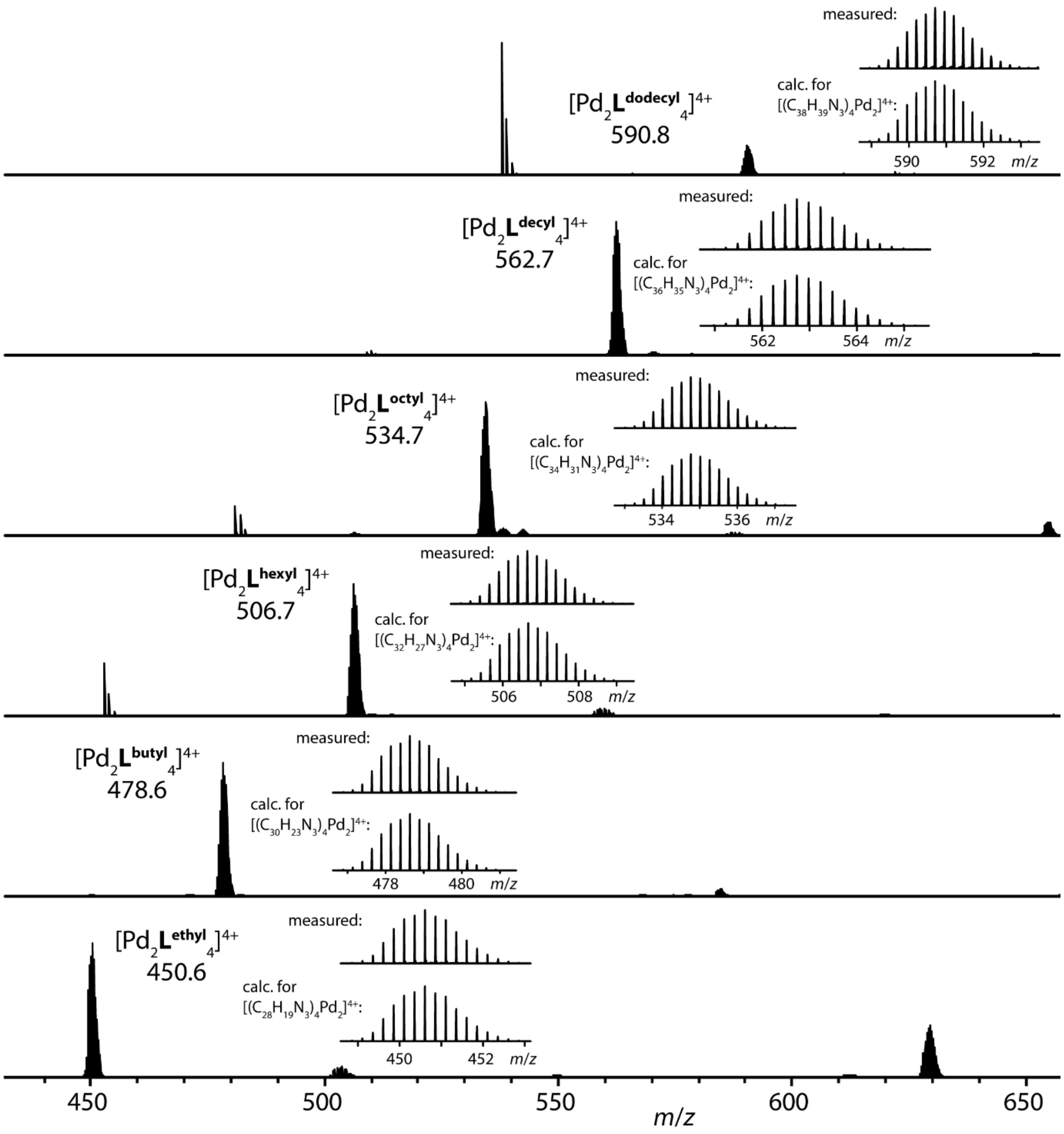


**Figure S3.** ESI MS spectra of $[Pd_2\mathbf{L}^R_4]^{4+}$.

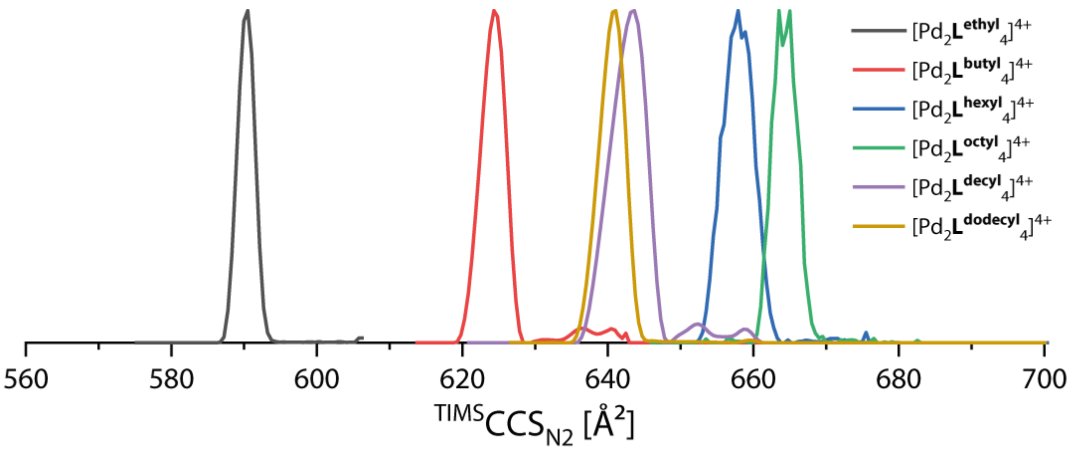


**Figure S4.** Overlaid TIMS mobilograms of $[Pd_2\mathbf{L}^{\mathbf{R}}_4]^{4+}$. Inverse mobilities were normalized and converted into CCS values.

### 1.2.4 Cage formation of $[3X@Pd_4\mathbf{L}^{\mathbf{R}}_8]^{5+}$

To the 0.4 mM monomeric cage solution, 22 µL of a 10 mM solution of $(Bu_4N)Br$ in $CD_3CN$ (1.0 cage eq.) was added. The resulting solution contained both monomeric cage $[Pd_2\mathbf{L}^{\mathbf{R}}_4]^{4+}$ and interpenetrated double cage $[3Br@Pd_4\mathbf{L}^{\mathbf{R}}_8]^{5+}$ in the equilibrated mixture.[9] For $\mathbf{L}^{\mathbf{ethyl}}$, alternatively, also a mixture with $(Bu_4N)Cl$ was prepared to form $[3Cl@Pd_4\mathbf{L}^{\mathbf{ethyl}}_8]^{5+}$.

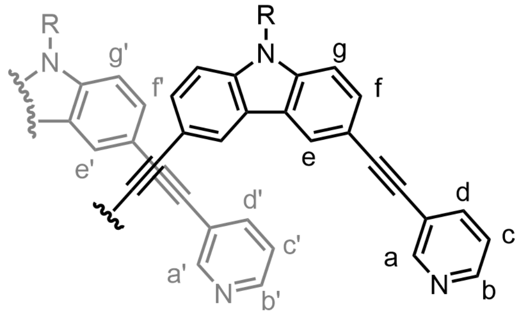


**Scheme S4.** Proton labeling for $[3Br@Pd_4\mathbf{L}^{\mathbf{R}}_8]^{5+}$. $a^L$: Proton a of free ligand, $a^M$: Proton a of monomeric cage, $a^D$, $a^{D'}$: Protons a and a' of double cage.

**$^1$H NMR (500 MHz, 298 K, $CD_3CN$) of** $[3Br@Pd_4\mathbf{L}^{\mathbf{ethyl}}_8]^{5+}$**:** $\delta$ [ppm] = 11.20 (s, 8H, $a^{D'}$), 10.57 (d, $J$ = 5.5 Hz, 8H, $b^{D'}$), 10.31 (s, 8H, $a^D$), 9.02 (s, $a^M$), 8.78 (m, $b^M$, $a^L$), 8.72 (d, $J$ = 5.6 Hz, 8H, $b^D$), 8.57 (s, $b^L$), 8.49 (s, $e^M$), 8.41 (s, $e^L$), 8.17 (d, $d^M$), 8.13 (d, $J$ = 8.0 Hz, 8H, $d^D$), 8.04 (d, $d^L$), 7.53-7.76 (m, $f^L$, $c^M$, $f^M$, $c^D$, $c^{D'}$, $g^D$, $f^D$, $d^{D'}$), 7.51 (dd, $c^L$), 6.88 (d, $J$ = 8.7 Hz, 8H, $g^{D'}$), 6.77 (s, 8H, $e^{D'}$), 6.73 (s, 8H, $e^D$), 6.06 (d, $J$ = 8.5 Hz, 8H, $f^{D'}$), 4.47 (m, $h^L$), 4.40 (q, $h^M$), 4.34 (q, $J$ = 7.1 Hz, 8H, $h^D$).

**$^1$H NMR (500 MHz, 298 K, $CD_3CN$) of** $[3Br@Pd_4\mathbf{L}^{\mathbf{butyl}}_8]^{5+}$**:** $\delta$ [ppm] = 11.20 (s, 8H, $a^{D'}$), 10.57 (d, $J$ = 5.5 Hz, 8H, $b^{D'}$), 10.31 (s, 8H, $a^D$), 9.02 (s, $a^M$), 8.78 (m, $b^M$, $a^L$), 8.72 (d, $J$ = 5.6 Hz, 8H, $b^D$), 8.55 (s, $b^L$), 8.48 (s, $e^M$), 8.39 (s, $e^L$), 8.18 (d, $d^M$), 8.14 (d, $J$ = 8.0 Hz, 8H, $d^D$), 7.95 (d, $d^L$), 7.51-7.76 (m, $f^L$, $c^M$, $f^M$, $c^D$, $c^{D'}$, $g^D$, $f^D$, $d^{D'}$), 7.42 (dd, $c^L$), 6.84 (d, $J$ = 8.7 Hz, 8H, $g^{D'}$), 6.76 (s, 8H, $e^{D'}$), 6.75 (s, 8H, $e^D$), 6.09 (d, $J$ = 8.5, 8H, $f^{D'}$), 4.40 (m, $h^L$), 4.34 (q, $h^M$), 4.27 (q, $J$ = 7.1 Hz, 8H, $h^D$).

**$^1$H NMR (500 MHz, 298 K, $CD_3CN$) of** $[3Br@Pd_4\mathbf{L}^{hexyl}{}_8]^{5+}$**:** $\delta$ [ppm] = 11.20 (s, 8H, $a^{D'}$), 10.57 (d, $J$ = 5.5 Hz, 8H, $b^{D'}$), 10.30 (s, 8H, $a^D$), 9.02 (s, $a^M$), 8.78 (m, $b^M$, $a^L$), 8.71 (d, $J$ = 5.6 Hz, 8H, $b^D$), 8.55 (s, $b^L$), 8.48 (s, $e^M$), 8.39 (s, $e^L$), 8.17 (d, $d^M$), 8.13 (d, $J$ = 8.0 Hz, 8H, $d^D$), 7.91 (d, $d^L$), 7.49-7.74 (m, $f^L$, $c^M$, $f^M$, $c^D$, $c^{D'}$, $g^D$, $f^D$, $d^{D'}$), 7.39 (dd, $c^L$), 6.82 (d, $J$ = 8.7 Hz, 8H, $g^{D'}$), 6.75 (s, 8H, $e^{D'}$), 6.74 (s, 8H, $e^D$), 6.06 (d, $J$ = 8.5 Hz, 8H, $f^{D'}$), 4.40 (m, $h^L$), 4.34 (q, $h^M$), 4.26 (q, $J$ = 7.1 Hz, 8H, $h^D$).

**$^1$H NMR (400 MHz, 298 K, $CD_3CN$) of** $[3Br@Pd_4\mathbf{L}^{octyl}{}_8]^{5+}$**:** $\delta$ [ppm] = 11.21 (s, 8H, $a^{D'}$), 10.57 (d, $J$ = 5.5 Hz, 8H, $b^{D'}$), 10.31 (s, 8H, $a^D$), 9.01 (s, $a^M$), 8.78 (m, $b^M$, $a^L$), 8.72 (d, $J$ = 5.6 Hz, 8H, $b^D$), 8.55 (s, $b^L$), 8.48 (s, $e^M$), 8.38 (s, $e^L$), 8.17 (d, $d^M$), 8.13 (d, $J$ = 8.0 Hz, 8H, $d^D$), 7.90 (d, $d^L$), 7.49-7.74 (m, $f^L$, $c^M$, $f^M$, $c^D$, $c^{D'}$, $g^D$, $f^D$, $d^{D'}$), 7.39 (dd, $c^L$), 6.82 (d, $J$ = 8.7 Hz, 8H, $g^{D'}$), 6.76 (s, 8H, $e^{D'}$), 6.74 (s, 8H, $e^D$), 6.07 (d, $J$ = 8.5 Hz, 8H, $f^{D'}$), 4.38 (m, $h^L$), 4.34 (q, $h^M$), 4.26 (q, $J$ = 7.1 Hz, 8H, $h^D$).

**$^1$H NMR (500 MHz, 298 K, $CD_3CN$) of** $[3Br@Pd_4\mathbf{L}^{decyl}{}_8]^{5+}$**:** $\delta$ [ppm] = 11.21 (s, 8H, $a^{D'}$), 10.58 (d, $J$ = 5.5 Hz, 8H, $b^{D'}$), 10.31 (s, 8H, $a^D$), 9.00 (s, $a^M$), 8.78 (m, $b^M$, $a^L$), 8.71 (d, $J$ = 5.6 Hz, 8H, $b^D$), 8.57 (s, $b^L$), 8.48 (s, $e^M$), 8.41 (s, $e^L$), 8.17 (d, $d^M$), 8.13 (d, $J$ = 8.0 Hz, 8H, $d^D$), 7.49-7.76 (m, $f^L$, $c^M$, $f^M$, $c^D$, $c^{D'}$, $g^D$, $f^D$, $d^{D'}$), 6.81 (d, $J$ = 8.7 Hz, 8H, $g^{D'}$), 6.76 (s, 8H, $e^{D'}$), 6.74 (s, 8H, $e^D$), 6.06 (d, $J$ = 8.5 Hz, 8H, $f^{D'}$), 4.40 (m, $h^L$), 4.34 (q, $h^M$), 4.26 (q, $J$ = 7.1 Hz, 8H, $h^D$).

**$^1$H NMR (500 MHz, 298 K, $CD_3CN$) of** $[3Br@Pd_4\mathbf{L}^{dodecyl}{}_8]^{5+}$**:** $\delta$ [ppm] = 11.21 (s, 8H, $a^{D'}$), 10.59 (d, $J$ = 5.5 Hz, 8H, $b^{D'}$), 10.32 (s, 8H, $a^D$), 9.01 (s, $a^M$), 8.78 (m, $b^M$, $a^L$), 8.72 (d, $J$ = 5.6 Hz, 8H, $b^D$), 8.55 (s, $b^L$), 8.48 (s, $e^M$), 8.39 (s, $e^L$), 8.17 (d, $d^M$), 8.13 (d, $J$ = 8.0 Hz, 8H, $d^D$), 7.90 (d, $d^L$), 7.48-7.74 (m, $f^L$, $c^M$, $f^M$, $c^D$, $c^{D'}$, $g^D$, $f^D$, $d^{D'}$), 7.39 (dd, $c^L$), 6.81 (d, $J$ = 8.7 Hz, 8H, $g^{D'}$), 6.76 (s, 8H, $e^{D'}$), 6.74 (s, 8H, $e^D$), 6.06 (d, $J$ = 8.5 Hz, 8H, $f^{D'}$), 4.34 (q, $h^M$), 4.26 (q, $J$ = 7.1 Hz, 8H, $h^D$).

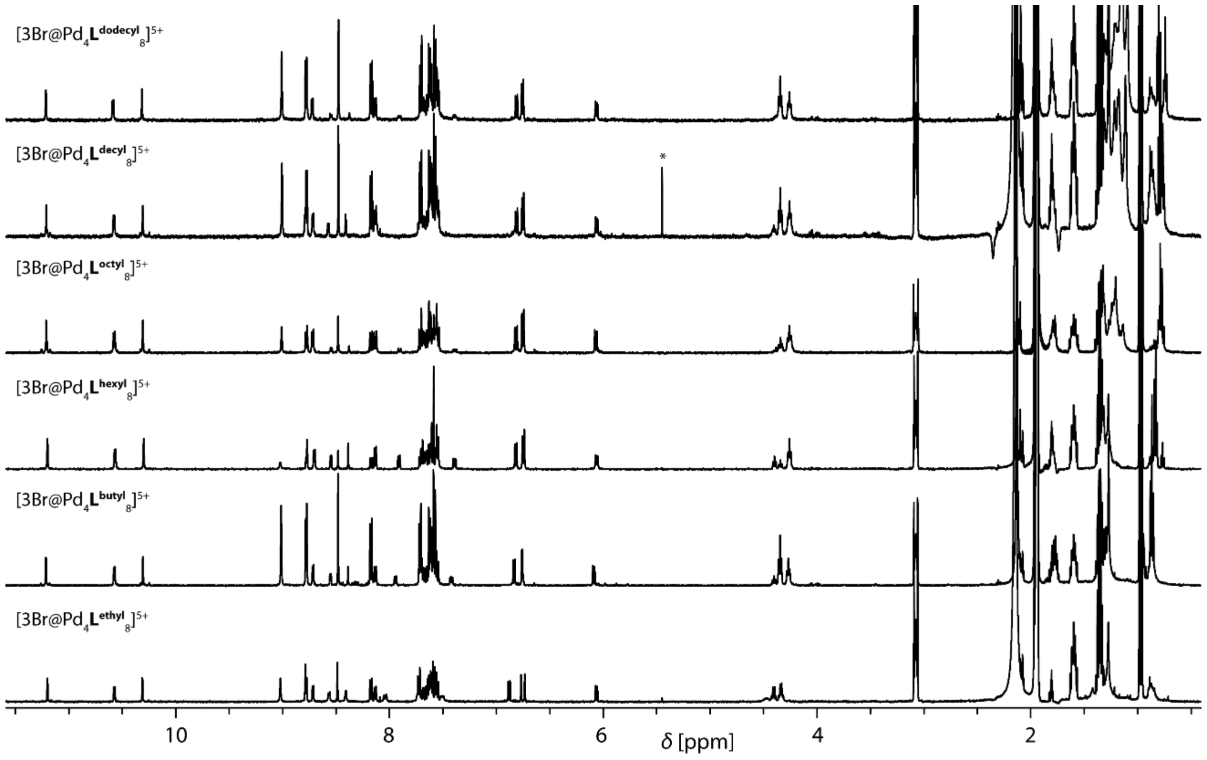


**Figure S5.** $^1$H NMR (298 K, $CD_3CN$) of $[3Br@Pd_4\mathbf{L}^{R}{}_8]^{5+}$.

### 1.2.5 ESI MS and TIMS spectra of $[3X@Pd_4L^R{}_8]^{5+}$

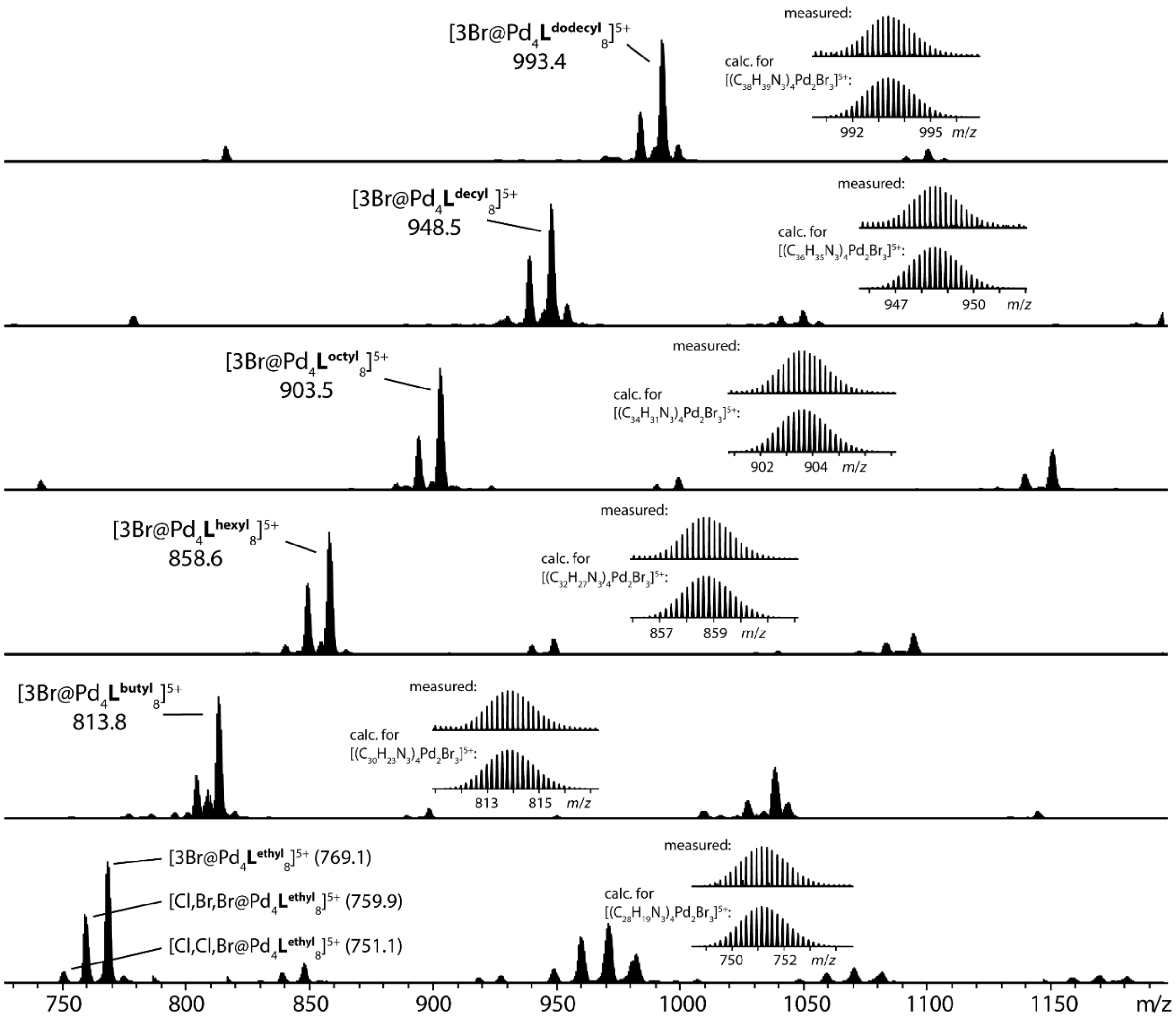


**Figure S6.** ESI MS spectra of $[3Br@Pd_4\mathbf{L}^R{}_8]^{5+}$.

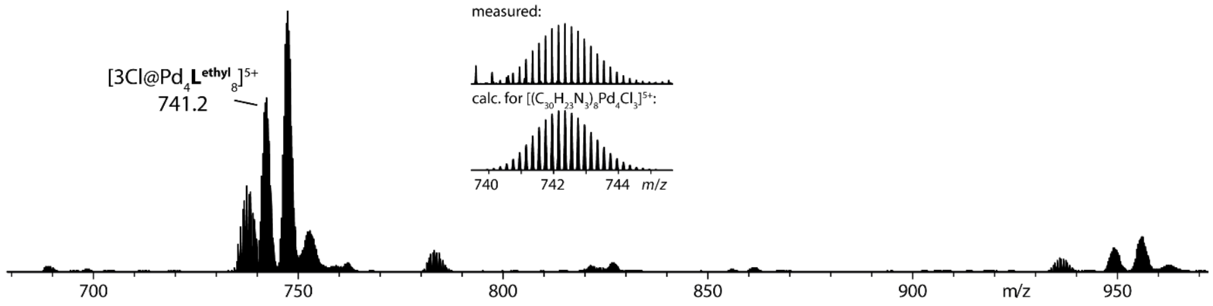


**Figure S7.** ESI MS spectrum of $[3Cl@Pd_4\mathbf{L}^{ethyl}{}_8]^{5+}$.

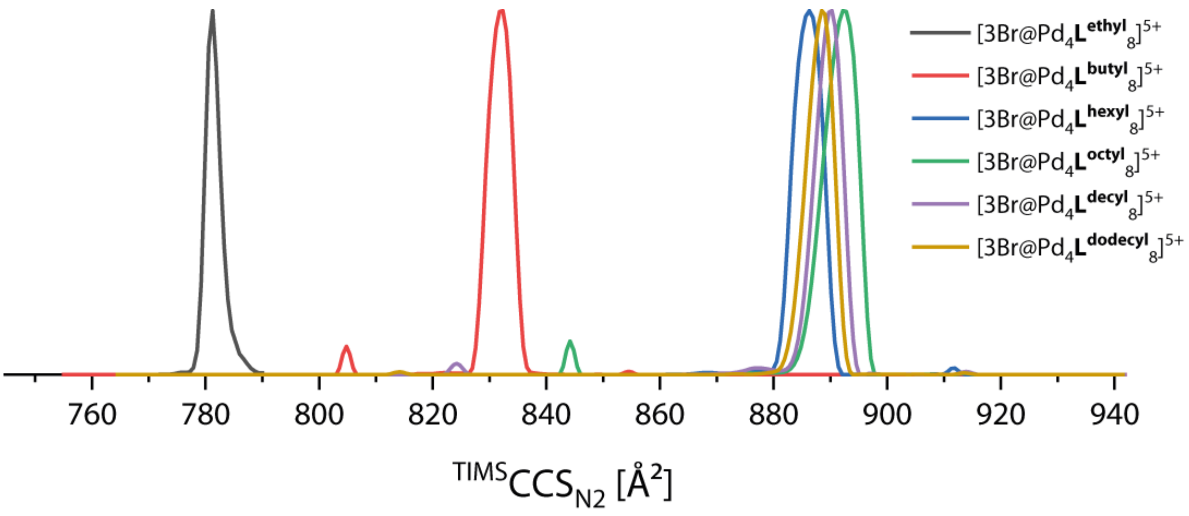


**Figure S8.** Overlaid TIMS mobilograms of $[3Br@Pd_4\mathbf{L}^{\mathbf{R}}_8]^{5+}$. Inverse mobilities were normalized and converted into CCS values.

### 1.2.6 DOSY NMR spectra of $Pd_2L^R_4$ and $3Br@Pd_4L^R_8$

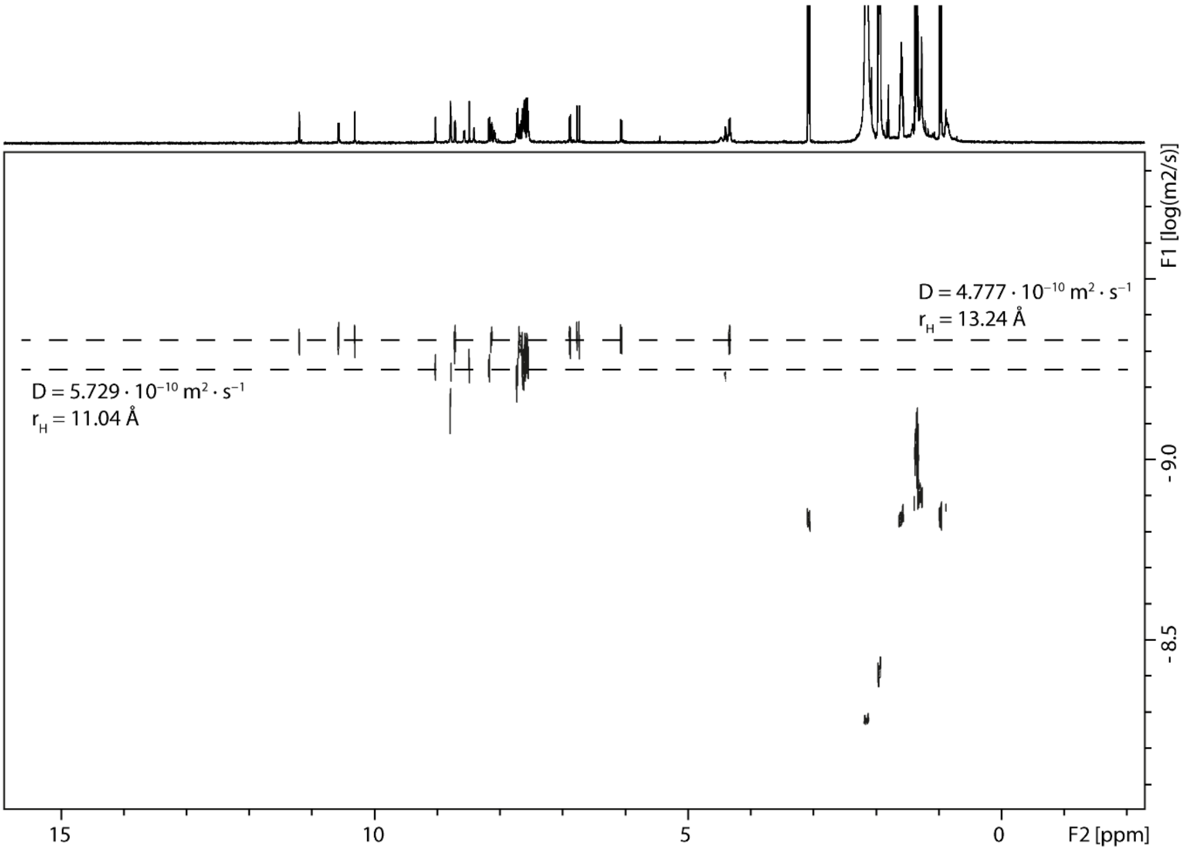


**Figure S9.** $^1H$ DOSY NMR (500 MHz, 298 K, $CD_3CN$) of a mixture of $Pd_2\mathbf{L}^{\mathbf{ethyl}}_4$ and $3Br@Pd_4\mathbf{L}^{\mathbf{ethyl}}_8$.

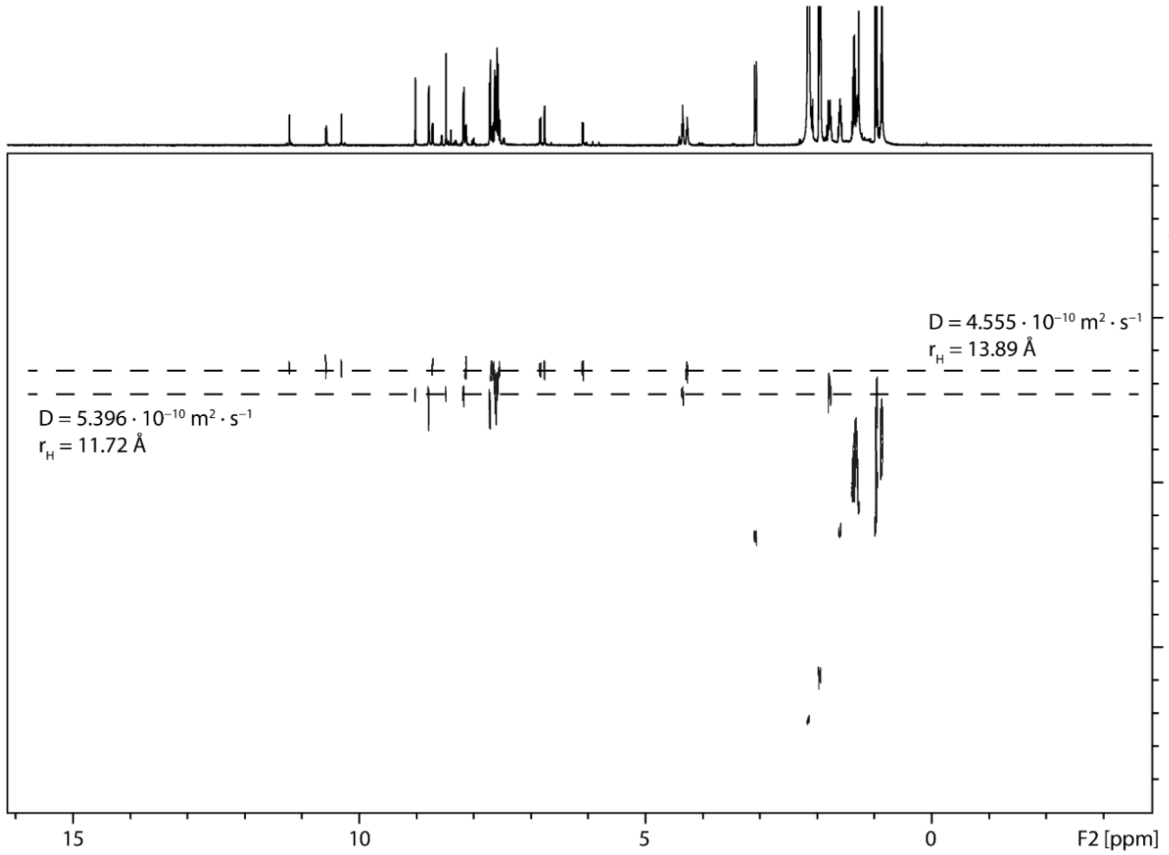


**Figure S10.** $^{1}$H DOSY NMR (500 MHz, 298 K, $CD_3CN$) of a mixture of $Pd_2$**L**$^{butyl}{}_4$ and 3Br@$Pd_4$**L**$^{butyl}{}_8$.

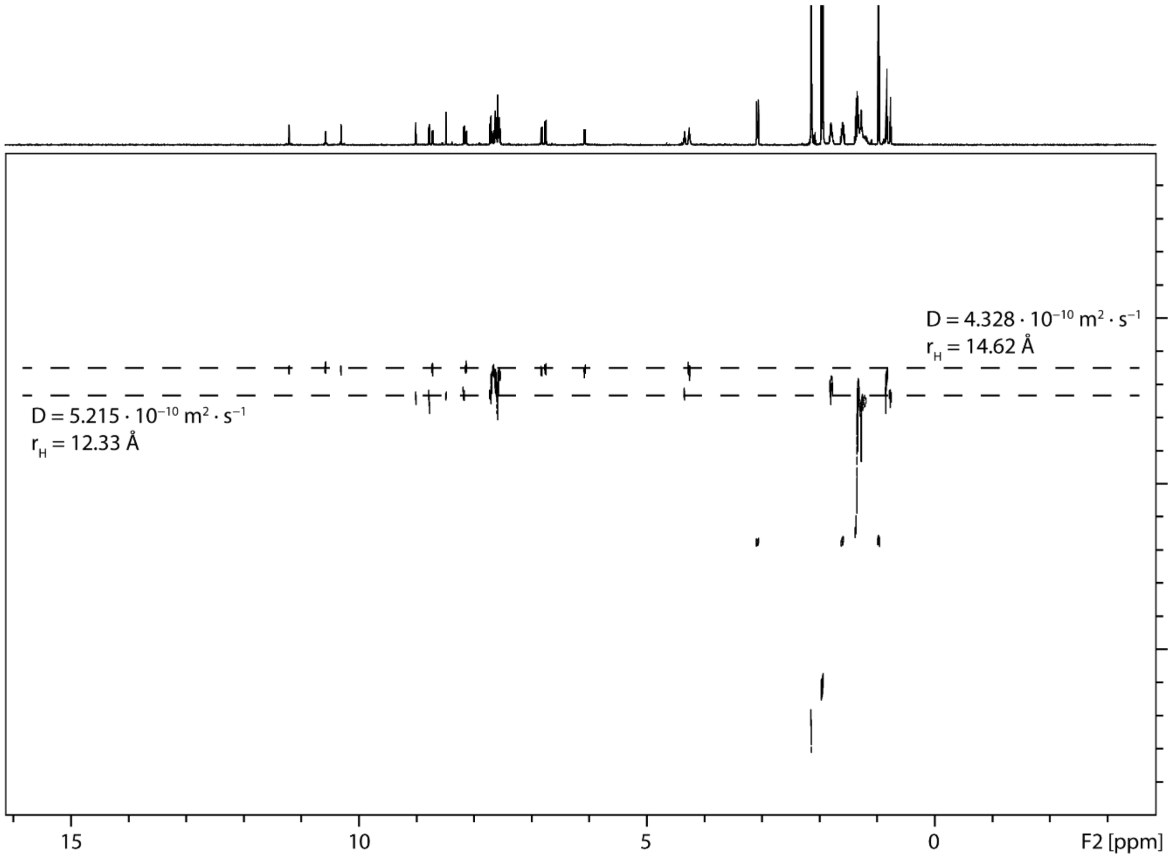


**Figure S11.** $^{1}$H DOSY NMR (500 MHz, 298 K, $CD_3CN$) of a mixture of $Pd_2$**L**$^{hexyl}{}_4$ and 3Br@$Pd_4$**L**$^{hexyl}{}_8$.

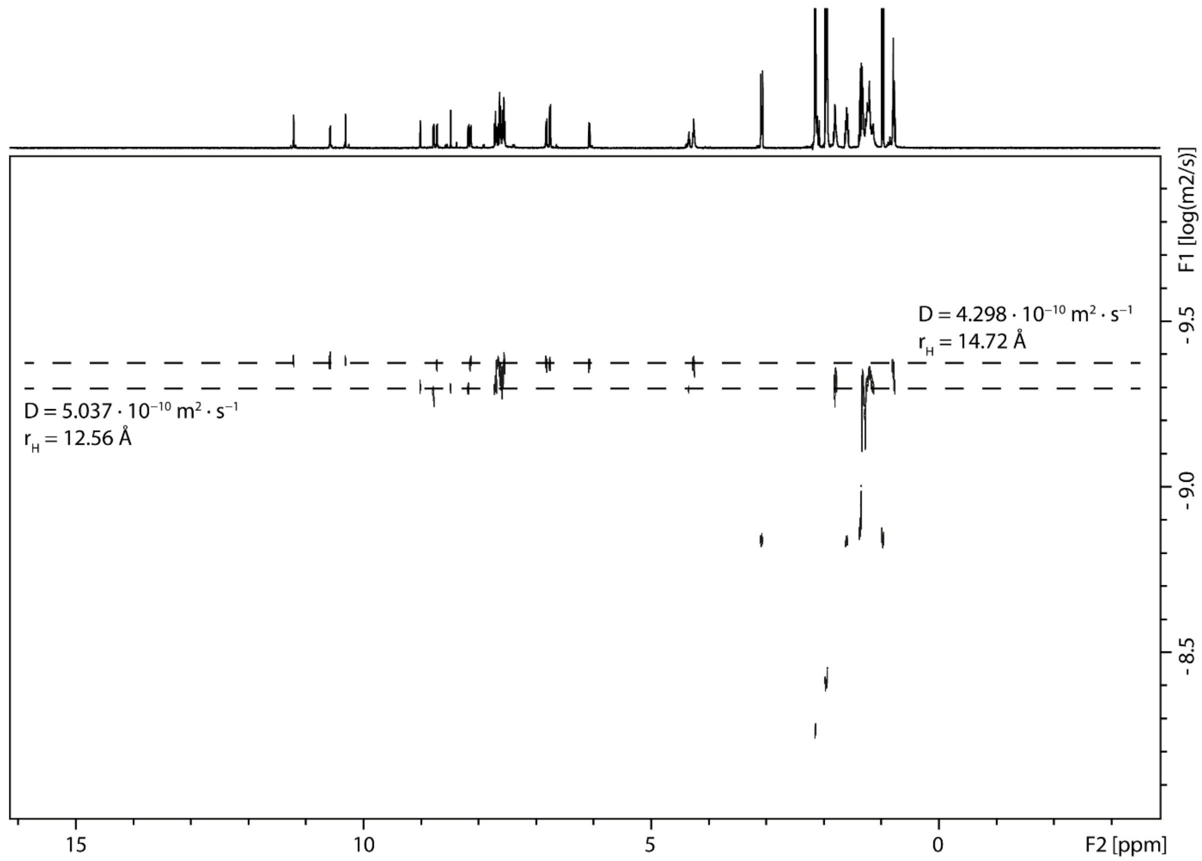


**Figure S12.** $^1$H DOSY NMR (500 MHz, 298 K, $CD_3CN$) of a mixture of $Pd_2\mathbf{L}^{\mathbf{octyl}}_4$ and $3Br@Pd_4\mathbf{L}^{\mathbf{octyl}}_8$.

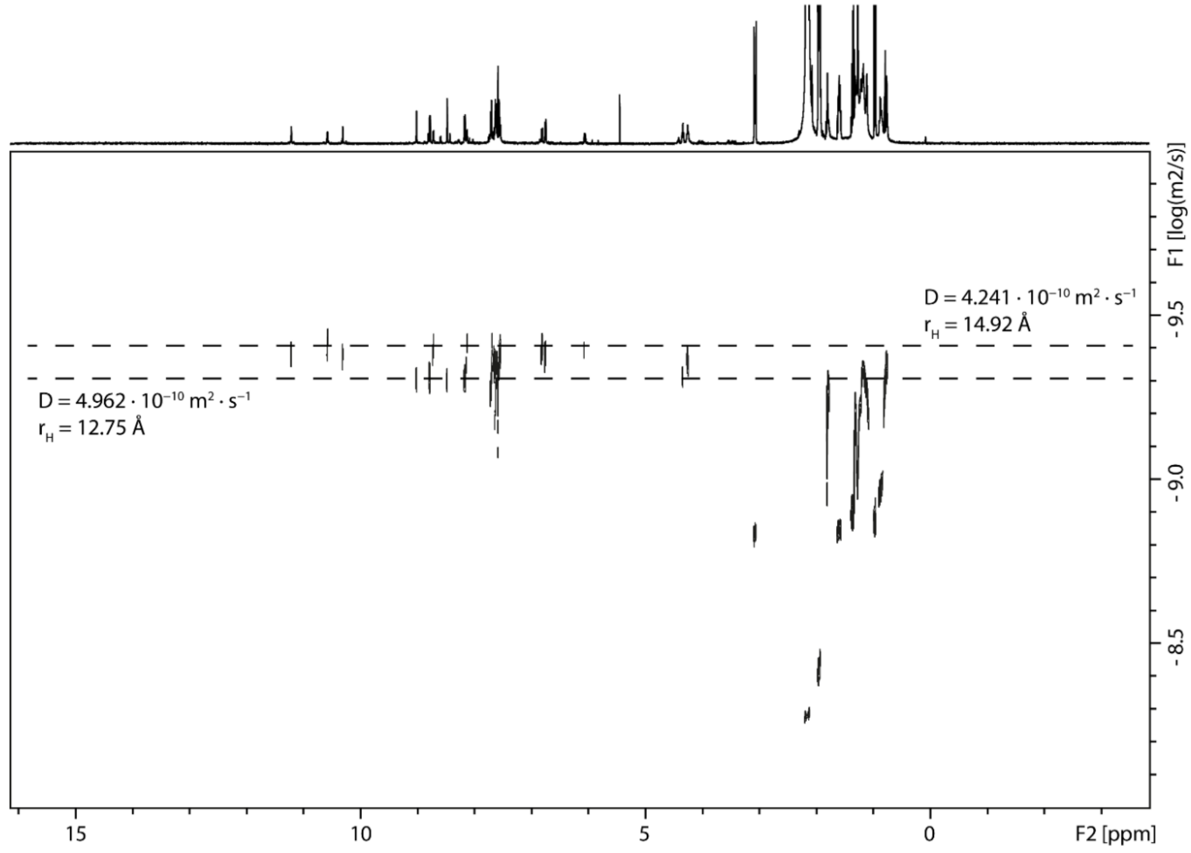


**Figure S13.** $^1$H DOSY NMR (500 MHz, 298 K, $CD_3CN$) of a mixture of $Pd_2\mathbf{L}^{\mathbf{decyl}}_4$ and $3Br@Pd_4\mathbf{L}^{\mathbf{decyl}}_8$.

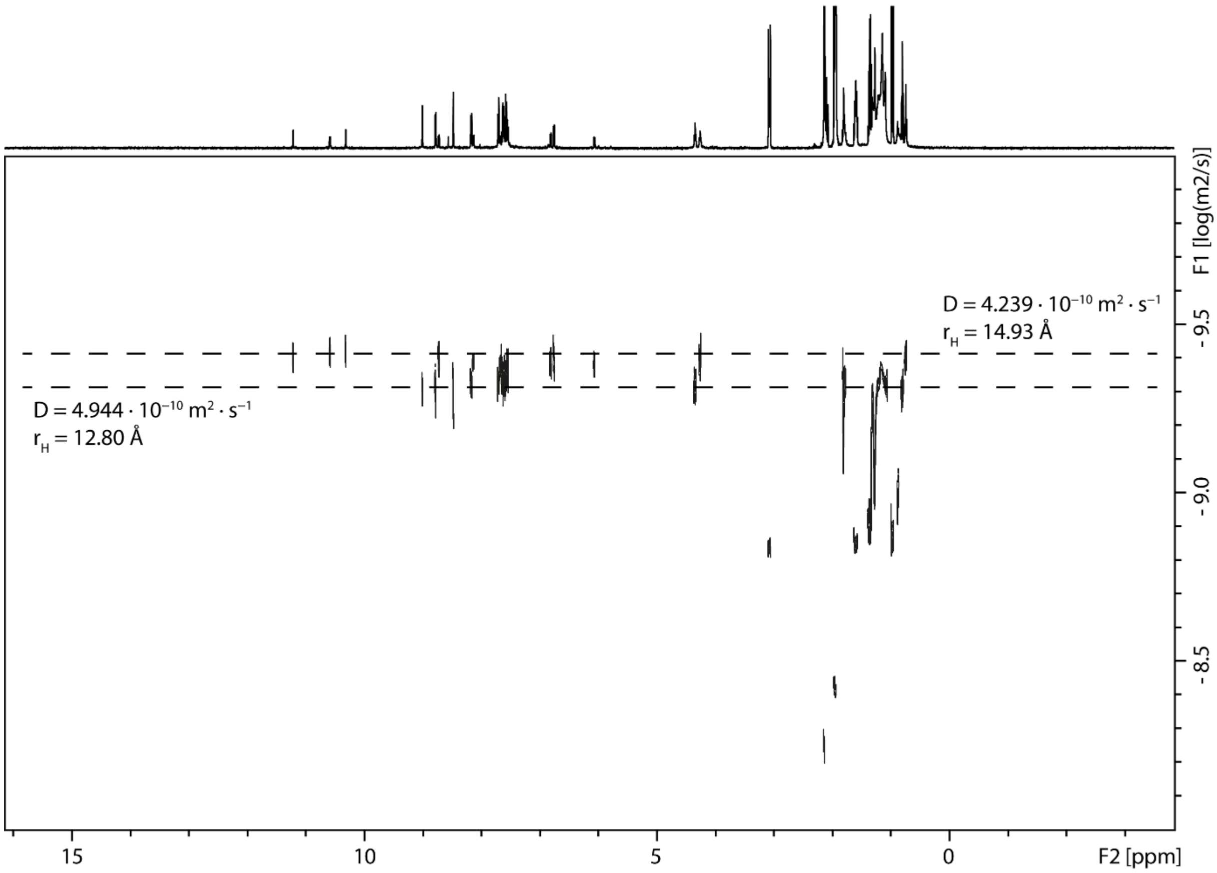


**Figure S14.** $^1$H DOSY NMR (500 MHz, 298 K, $CD_3CN$) of a mixture of $Pd_2$**L**$^{dodecyl}{}_4$ and 3Br@$Pd_4$**L**$^{dodecyl}{}_8$.

**Table S1.** Diffusion coefficients *D*, solvodynamic radii $r_S$ and relative increase of the latter with increasing chain length for all monomeric and double cages.

| Species | *D* [$10^{-10}m^2s^{-1}$] | $r_S$ [Å] | rel. increase |
|---|---|---|---|
| $Pd_2$**L**$^{ethyl}{}_4$ | 5.7 ± 0.4 | 11.1 ± 0.8 | - |
| $Pd_2$**L**$^{butyl}{}_4$ | 5.4 ± 0.2 | 11.7 ± 0.4 | 6 ± 3 % |
| $Pd_2$**L**$^{hexyl}{}_4$ | 5.47 ± 0.07 | 11.6 ± 0.1 | 4 ± 1 % |
| $Pd_2$**L**$^{octyl}{}_4$ | 5.0 ± 0.2 | 12.6 ± 0.5 | 13 ± 5 % |
| $Pd_2$**L**$^{decyl}{}_4$ | 5.0 ± 0.6 | 12.8 ± 0.2 | 15 ± 1 % |
| $Pd_2$**L**$^{dodecyl}{}_4$ | 4.9 ± 0.2 | 12.8 ± 0.4 | 15 ± 4 % |
| 3Br@$Pd_4$**L**$^{ethyl}{}_8$ | 4.8 ± 0.1 | 13.2 ± 0.3 | - |
| 3Br@$Pd_4$**L**$^{butyl}{}_8$ | 4.6 ± 0.1 | 13.9 ± 0.3 | 5 ± 2 % |
| 3Br@$Pd_4$**L**$^{hexyl}{}_8$ | 4.53 ± 0.03 | 14.0 ± 0.1 | 5 ± 1 % |
| 3Br@$Pd_4$**L**$^{octyl}{}_8$ | 4.3 ± 0.2 | 14.7 ± 0.5 | 11 ± 4 % |
| 3Br@$Pd_4$**L**$^{decyl}{}_8$ | 4.2 ± 0.2 | 14.9 ± 0.6 | 13 ± 4 % |
| 3Br@$Pd_4$**L**$^{dodecyl}{}_8$ | 4.2 ± 0.2 | 15.0 ± 0.7 | 13 ± 5 % |

## 1.3 Synthesis and characterization of [$Pd_2$L$^{methyl}{}_4$]$^{4+}$

### 1.3.1 Synthesis of L$^{methyl}$

**L**$^{methyl}$ was prepared following the procedure described in Section 1.2.1 but using iodomethane instead of bromoalkanes.

**$^1$H NMR (500 MHz, 298 K, $CD_3CN$) of L$^{methyl}$:** $\delta$ [ppm] = 8.77 (s, 2H, a), 8.55 (d, $J$ = 4.9 Hz, 2H, b), 8.39 (s, 2H, e), 7.91 (d, $J$ = 7.9 Hz, 2H, d), 7.73 (d, $J$ = 8.5, 2H, f), 7.59 (d, $J$ = 8.5, 2H, g), 7.39 (dd, $J_1$ = 7.9 Hz, $J_2$ = 4.8 Hz, 2H, c), 3.91 (s, 3H, h).

**$^{13}$C{$^1$H} NMR (126 MHz, 298 K, DMSO-d$_6$) of L$^{methyl}$:** $\delta$ [ppm] = 151.48, 148.68, 141.08, 138.33, 129.83, 124.46, 123.74, 121.78, 120.04, 118.14, 112.38, 110.25, 93.92, 84.69.

**ESI MS of L$^{methyl}$:** measured: 384.1477, calculated for $[C_{27}H_{17}N_3+H]^+$: 384.1495.

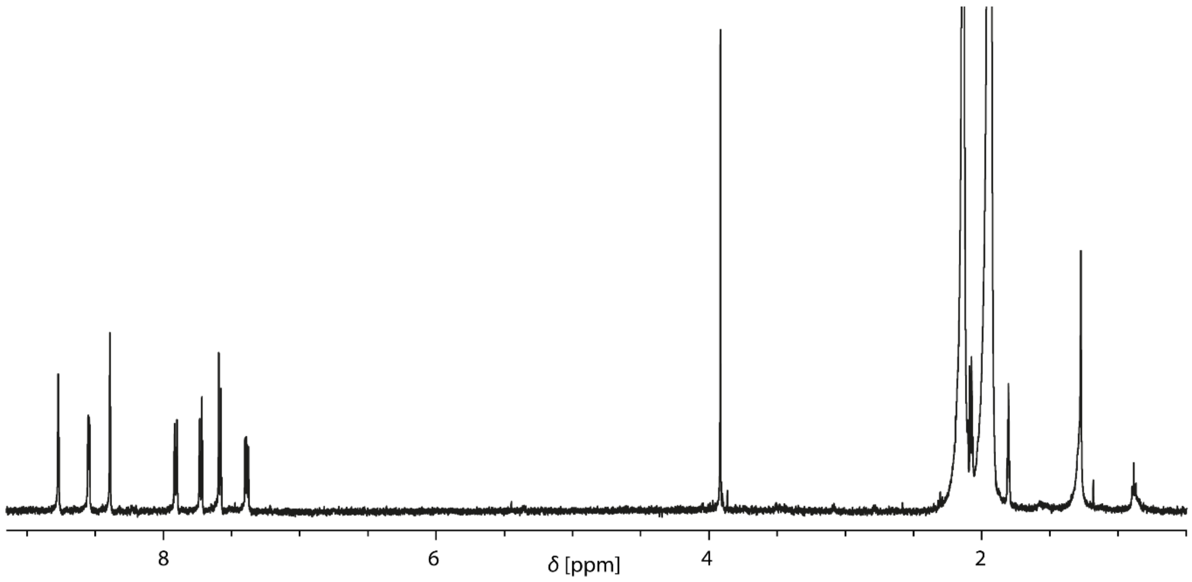


**Figure S15.** $^1$H NMR (500 MHz, 298 K, $CD_3CN$) of **L$^{methyl}$**.

## 1.3.2 Cage formation of $[Pd_2L^{methyl}_4]^{4+}$

To form $[Pd_2\mathbf{L}^{methyl}_4]^{4+}$, the procedure described in Section 1.2.2 was followed.

**$^1$H NMR (500 MHz, 298 K, $CD_3CN$) of** $[Pd_2\mathbf{L}^{methyl}_4]^{4+}$**:** $\delta$ [ppm] = 8.98 (s, 8H, a), 8.76 (d, $J$ = 5.9 Hz, 8H, b), 8.48 (s, 8H, e), 8.17 (d, $J$ = 8.2 Hz, 8H, d), 7.74 (d, $J$ = 8.5 Hz, 8H, f), 7.62 (dd, $J_1$ = 8.2 Hz, $J_2$ = 5.9 Hz, 8H, c), 7.56 (d, $J$ = 8.6 Hz, 8H, g), 3.85 (s, 12H, h).

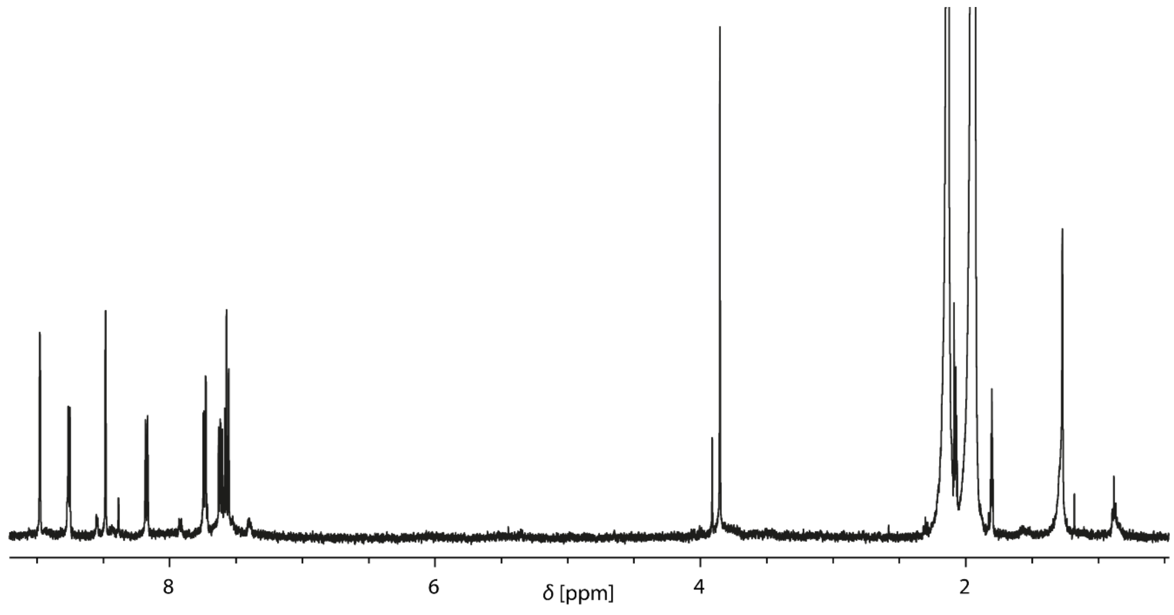


**Figure S16.** $^1$H NMR (500 MHz, 298 K, $CD_3CN$) of $[Pd_2\mathbf{L}^{methyl}_4]^{4+}$.

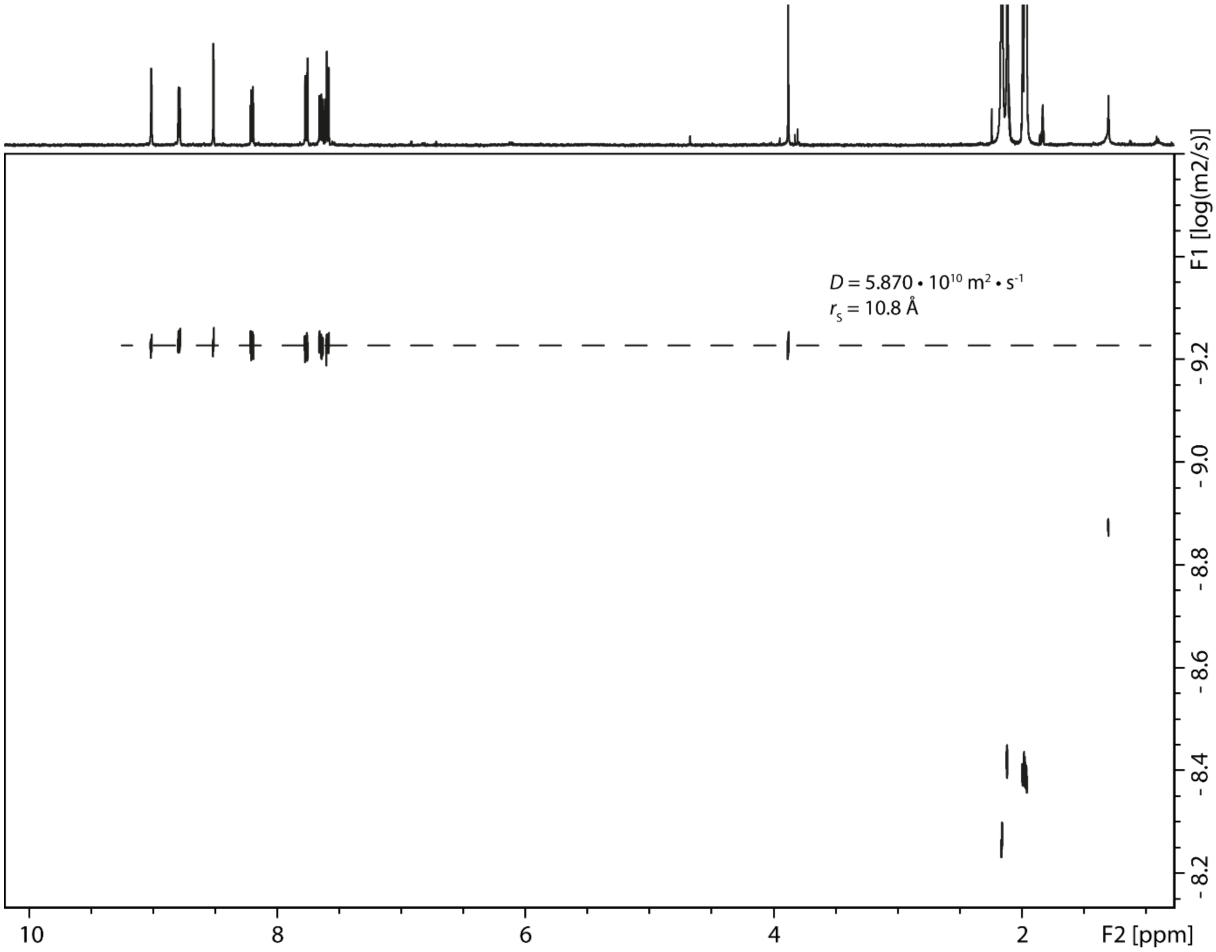


**Figure S17.** $^{1}$H DOSY NMR (500 MHz, 298 K, $CD_3CN$) of $Pd_2\mathbf{L}^{\mathbf{methyl}}{}_4$.

### 1.3.3 ESI MS and TIMS spectra of $[Pd_2\mathbf{L}^{\mathbf{methyl}}{}_4]^{4+}$

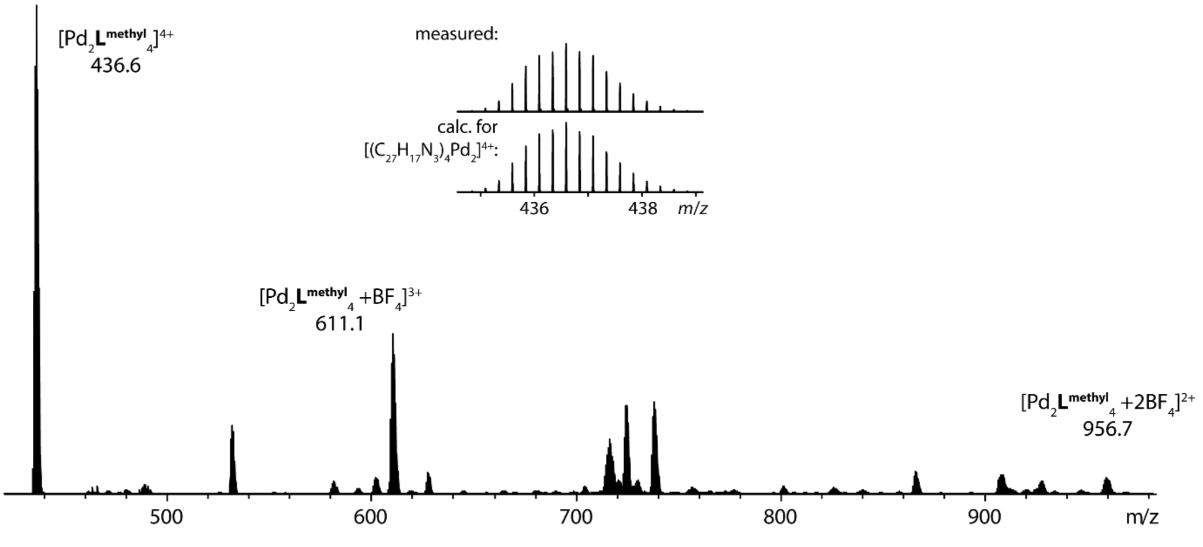


**Figure S18.** ESI MS spectrum of $[Pd_2\mathbf{L}^{\mathbf{methyl}}{}_4]^{4+}$.

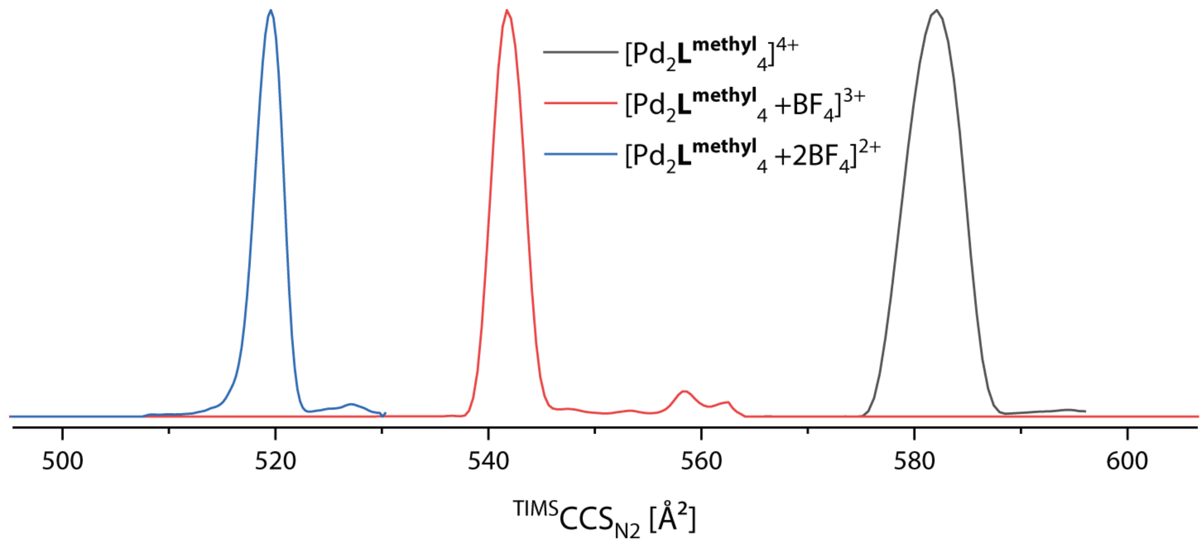


**Figure S19.** Overlaid TIMS mobilograms of $[Pd_2\mathbf{L}^{methyl}{}_4](BF_4)_4$. Inverse mobilities were normalized and converted into CCS values.

## 1.4 Synthesis and characterization of $[Pd_2L^{phenyl}{}_4]^{4+}$

### 1.4.1 Synthesis of $L^{phenyl}$

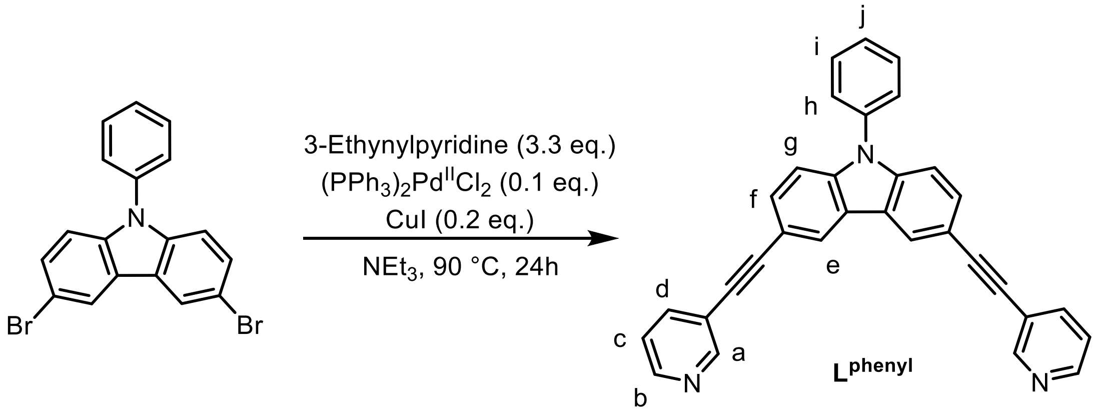


**Scheme S5.** Synthesis of $\mathbf{L}^{phenyl}$.

For the synthesis of $\mathbf{L}^{phenyl}$, 3,6-dibromo-9-phenylcarbazole (1.0 eq., 0.29 mmol, 100 mg) and 3-ethynylpyridine (3.3 eq., 0.82 mmol, 84.7 mg) were dissolved in a mixture of 5 mL THF and 2 mL $NEt_3$ and degassed with three *freeze-pump-thaw*-cycles. After the addition of $(PPh_3)_2Pd(II)Cl_2$ (0.1 eq., 0.025 mmol, 17.5 mg) and CuI (0.2 eq., 0.050 mmol, 9.48 mg) three more *freeze-pump-thaw*-cycles were done. The reaction mixture was then heated and stirred at 90 °C for 24 h. After the reaction the solvent was removed *in vacuo* and the solids were adsorbed to silica. The product could be obtained after purification with automated flash chromatography (100 % EtOAc) and gel permeation chromatography as a white-yellow solid (81 %).

**$^1$H NMR (500 MHz, 298 K, $CD_3CN$) of $\mathbf{L}^{phenyl}$:** $\delta$ [ppm] = 8.78 (s, 2H, a), 8.56 (d, $J$ = 4.9 Hz, 2H, b), 8.46 (s, 2H, e), 7.92 (d, $J$ = 7.9 Hz, 2H, d), 7.57-7.74 (m, 7H, f, h, i, j), 7.42 (d, $J$ = 8.5 Hz, 2H, g) 7.39 (dd, $J_1$ = 7.9 Hz, $J_2$ = 4.8 Hz, 2H, c).

**$^{13}$C{$^1$H} NMR (126 MHz, 298 K, DMSO-$d_6$) of $\mathbf{L}^{phenyl}$:** $\delta$ [ppm] = 151.5, 148.8, 140.7, 138.4, 135.8, 130.4, 128.5, 126.9, 124.7, 123.7, 122.5, 121.7, 119.9, 113.7, 110.5, 93.5, 85.0.

**ESI MS of $\mathbf{L}^{phenyl}$:** measured: 445.1566, calculated for $[C_{32}H_{19}N_3+H]^+$: 445.1573.

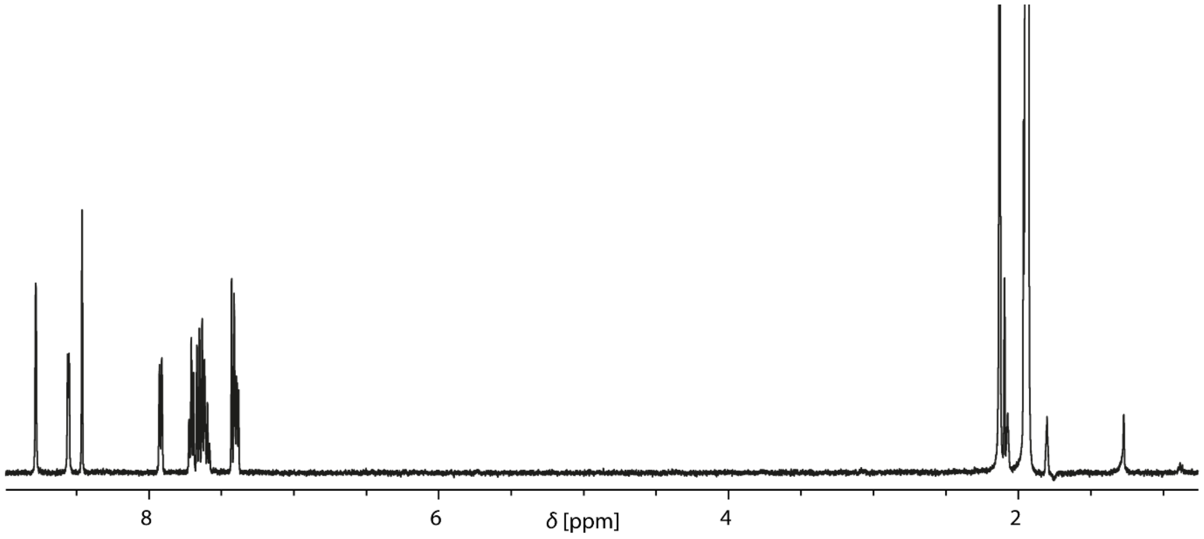


**Figure S20.** $^1$H NMR (500 MHz, 298 K, $CD_3CN$) of **L$^{phenyl}$**.

### 1.4.2 Cage formation of $[Pd_2L^{phenyl}{}_4]^{4+}$

To form $[Pd_2$**L**$^{phenyl}{}_4]^{4+}$, the procedure described in 1.2.2 was followed.

**$^1$H NMR (500 MHz, 298 K, $CD_3CN$) of** $[Pd_2$**L**$^{phenyl}{}_4]^{4+}$**:** $\delta$ [ppm] = 9.02 (s, 8H, a), 8.77 (d, *J* = 5.9 Hz, 8H, b), 8.59 (s, 8H, e), 8.19 (d, *J* = 8.2 Hz, 8H, d), 7.51-1.71 (m, 35H, c, f, h, i, j), 7.40 (d, *J* = 8.6 Hz, 8H, g), 3.85 (s, 12H, h).

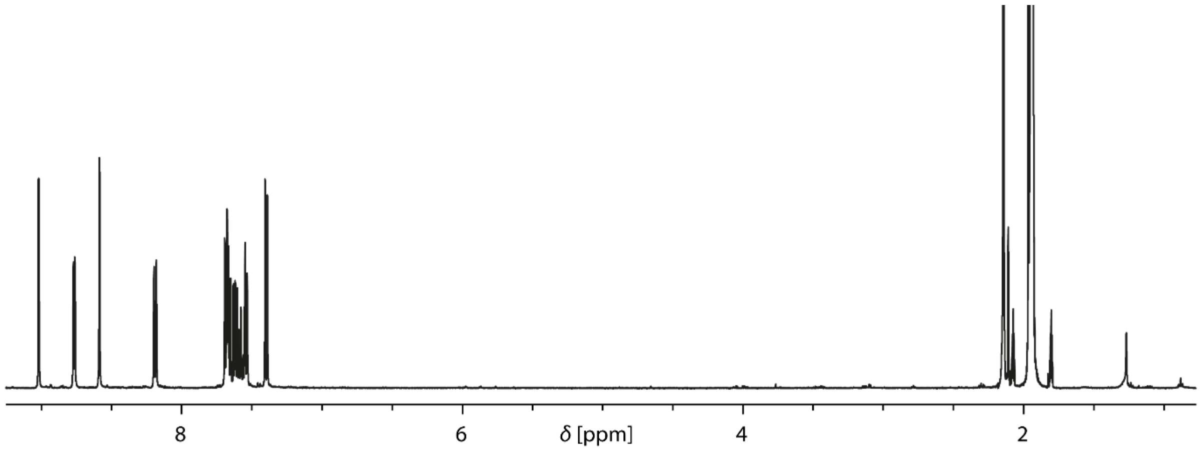


**Figure S21.** $^1$H NMR (500 MHz, 298 K, $CD_3CN$) of $[Pd_2$**L**$^{phenyl}{}_4]^{4+}$.

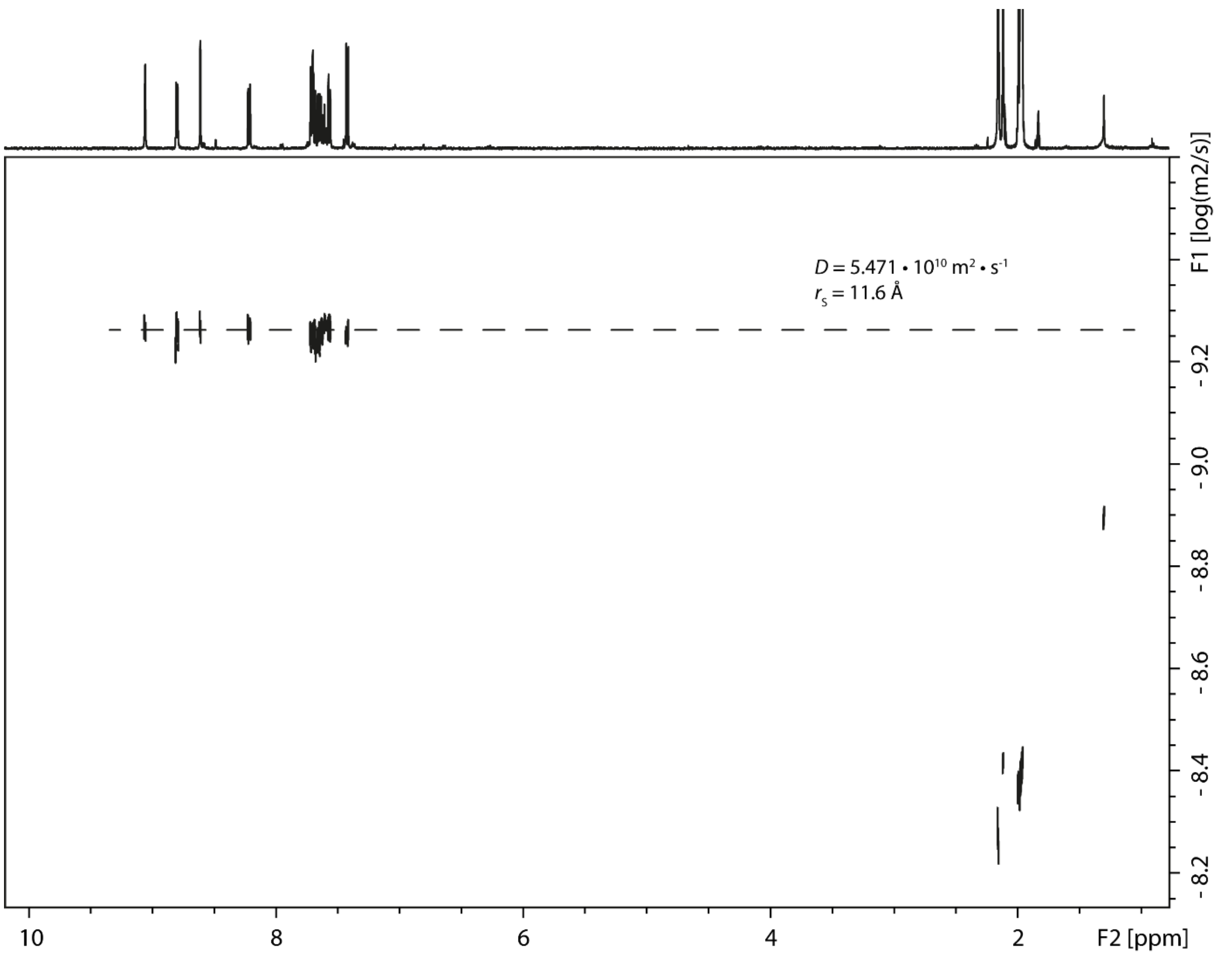


**Figure S22.** $^1$H DOSY NMR (500 MHz, 298 K, $CD_3CN$) of $[Pd_2\mathbf{L}^{phenyl}{}_4]^{4+}$.

## 1.4.3 ESI MS and TIMS spectra of $[Pd_2L^{phenyl}{}_4]^{4+}$

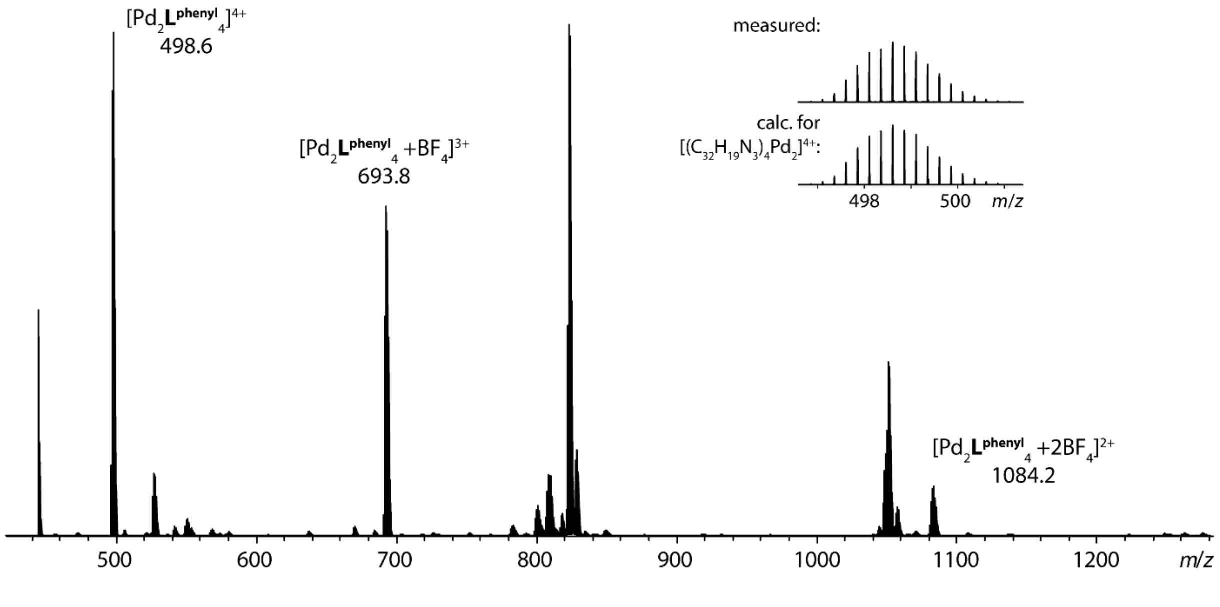


**Figure S23.** ESI MS spectrum of $[Pd_2\mathbf{L}^{phenyl}{}_4](BF_4)_4$.

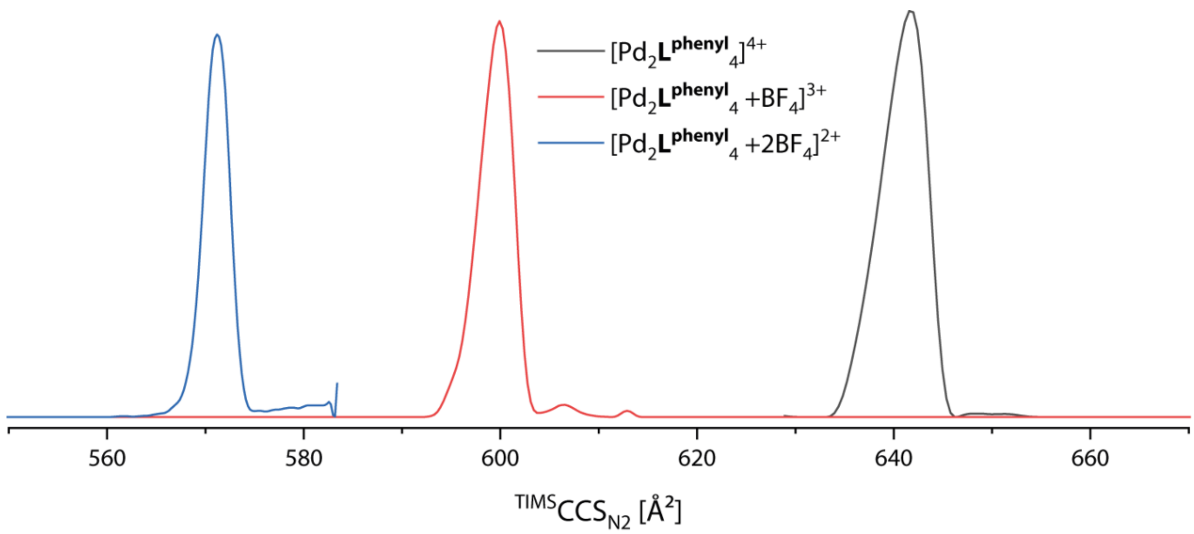


**Figure S24.** Overlaid TIMS mobilograms of $[Pd_2\mathbf{L}^{phenyl}_4](BF_4)_4$. Inverse mobilities were normalized and converted into CCS values.

## 1.5 Synthesis and characterization of $[Pd_2L^{pyrenyl}_4]^{4+}$

### 1.5.1 Synthesis of $L^{pyrenyl}$

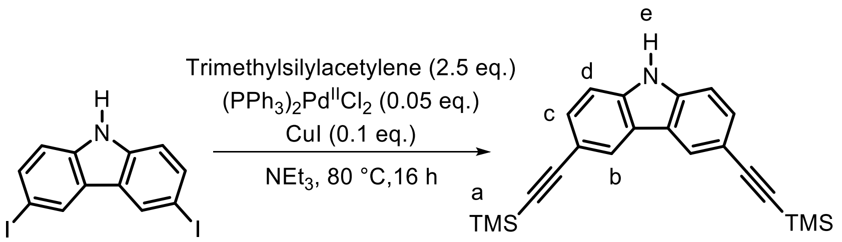


**1**

**Scheme S6.** Synthesis of **1**.

3,6-Diiodocarbazole (1.0 eq., 0.48 mmol, 200 mg) was dissolved in 10 mL $NEt_3$. To that solution trimethylsilylacetylene (2.5 eq., 1.19 mmol, 170 µL) was added. The solution was degassed with three *freeze-pump-thaw*-cycles. After the addition of $(PPh_3)_2Pd(II)Cl_2$ (0.05 eq., 0.024 mmol, 16.7 mg) and CuI (0.1 eq., 0.048 mmol, 9.08 mg) three more *freeze-pump-thaw*-cycles were done. The reaction mixture was then heated and stirred at 80 °C for 16 h. After the reaction the solvent was removed *in vacuo* and the solids were adsorbed on silica. Compound **1** could be obtained after purification with automated flash chromatography (*n*-pentane/EtOAc, 0 % → 25 %) as a white-brown solid (79 %). The product should be stored at –20 °C under an inert atmosphere.

**$^1$H NMR (600 MHz, 298 K, DMSO-d$_6$) of 1:** $\delta$ [ppm] = 11.7 (s, 1H, e), 8.36 (s, 2H, b), 7.47 (m, 4H, c, d), 0.25 (s, 18H, a).

**$^{13}$C{$^1$H} NMR (151 MHz, 298 K, DMSO-d$_6$) of 1:** $\delta$ [ppm] = 140.04, 129.64, 124.71, 122.04, 112.65, 111.58, 107.06, 91.62, 0.14.

**ESI MS of 1:** measured: 358.1435, calculated for $[C_{22}H_{24}NSi_2]^-$: 358.1453.

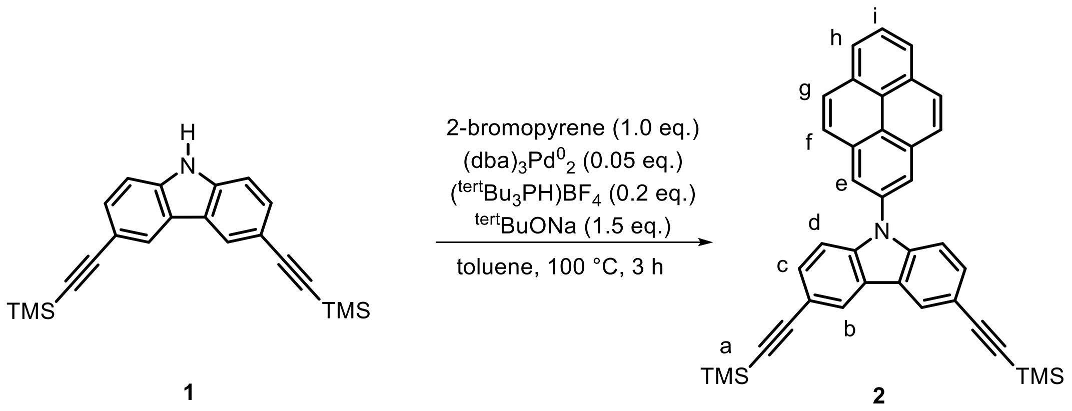


**Scheme S7.** Synthesis of **2**.

**1** (1.0 eq., 0.14 mmol, 50 mg), 2-bromopyrene (1.0 eq., 0.14 mmol, 39.1 mg) and $^{tert}$BuONa (1.5 eq., 0.21 mmol, 20.0 mg) were dissolved in 3 mL toluene and the solution was degassed with three *freeze-pump-thaw*-cycles. After the addition of $(dba)_3Pd(0)_2$ (0.05 eq., 7.0 µmol, 8.1 mg) and $(^{tert}Bu_3PH)BF_4$ (0.2 eq., 27.8 µmol, 8.1 mg) three more *freeze-pump-thaw*-cycles were done. The reaction mixture was heated at 100 °C for 3 h. The reaction mixture was allowed to cool down and then the solvent was removed *in vacuo* and the remaining solids were adsorbed on silica. Automated flash chromatography (n-pentane/$CHCl_3$, 0 % → 20 %) yielded compound **3** as a white-yellow solid (39 %).

**$^1$H NMR (600 MHz, 298 K, DMSO) of 3:** $\delta$ [ppm] = 8.60 (s, 2H, b), 8.58 (s, 2H, e), 8.42 (d, $J$ = 7.7 Hz, 2H, f), 8.31-8.37 (m, 4H, g, h), 8.18 (t, $J$ = 7.7 Hz, i), 7.56 (d, $J$ = 8.5 Hz, c), 7.48 (d, $J$ = 8.4 Hz, d), 0.28 (s, 18H, a).

**$^{13}$C{$^1$H} NMR (151 MHz, 298 K, DMSO-d$_6$) of 2:** $\delta$ [ppm] = 134.57, 132.85, 131.28, 130.58, 128.95, 127.09, 126.63, 126.04, 124.89, 124.50, 124.14, 123.07, 122.94, 115.25, 110.01, 106.31, 92.66, 55.96, 0.29.

No mass spectrum of compound **2** could be obtained.

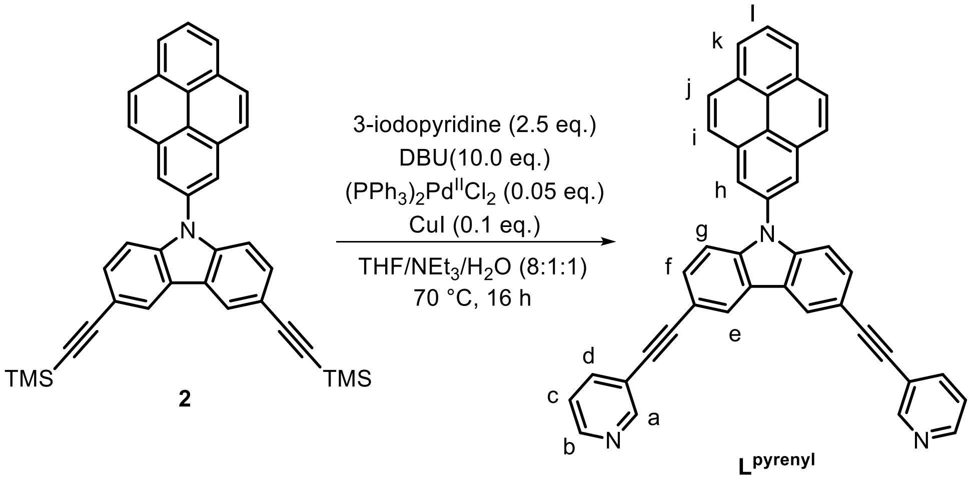


**Scheme S8.** Synthesis of **L$^{pyrenyl}$**.

Compound **2** (1.0 eq., 54.8 µmol, 30.7 mg), 3-iodopyridine (2.5 eq., 137 µmol, 28.1 mg) and 1,8-diazabicyclo[5.4.0]-7-undecene (10.0 eq., 548 µmol, 83.4 mg) were dissolved in 2 mL of a mixture of THF, $NEt_3$ and $H_2O$ (8:1:1) and it was degassed with three *freeze-pump-thaw*-cycles. After the addition of $(PPh_3)_2Pd(II)Cl_2$ (0.05 eq., 2.74 µmol, 1.9 mg) and CuI (0.1 eq., 5.5 µmol, 1.0 mg) three more

*freeze-pump-thaw*-cycles were done. The reaction mixture was then heated and stirred at 70 °C for 16 h. After extraction with DCM (three times) most of the solvent was removed and automated flash chromatography (liquid loading, first *n*-pentane/ $CHCl_3$ 0 % → 100 %, then chloroform/methanol 0 % → 10 %) and afterwards washing with $CH_3CN$ yielded $\mathbf{L^{pyrenyl}}$ as a yellow solid (95 %).

**$^1$H NMR (500 MHz, 298 K, DMSO) of $\mathbf{L^{pyrenyl}}$:** *δ* [ppm] = 8.81 (s, 2H, a), 8.73 (s, 2H, e), 8.64 (s, 2H, h), 8.60 (d, *J* = 5.0 Hz, 2H, b), 8.44 (d, *J* = 7.8 Hz, 2H, i), 8.36 (m, 4H, k, j, k), 8.19 (t, *J* = 7.6 Hz, 1H, l), 8.03 (d, *J* = 8.0 Hz, 2H, d), 7.73 (d, *J* = 8.5 Hz, 2H, f), 7.59 (d, *J* = 8.4 Hz, 2H, g), 7.50 (dd, $J_1$ = 7.8 Hz, $J_2$ = 4.8 Hz, 2H, c).

**$^{13}$C{$^1$H} NMR (151 MHz, 298 K, DMSO-d$_6$) of $\mathbf{L^{pyrenyl}}$:** *δ* [ppm] = 151.49, 148.74, 141.25, 138.43, 132.35, 130.67, 130.50, 128.69, 127.18, 126.84, 125.97, 124.81, 123.77, 123.48, 123.19, 122.85, 122.61, 119.90, 113.98, 110.66, 93.54, 85.14.

**ESI MS of $\mathbf{L^{pyrenyl}}$:** measured: 570.1980, calculated for $[C_{42}H_{23}N_3+H]^+$: 570.1980.

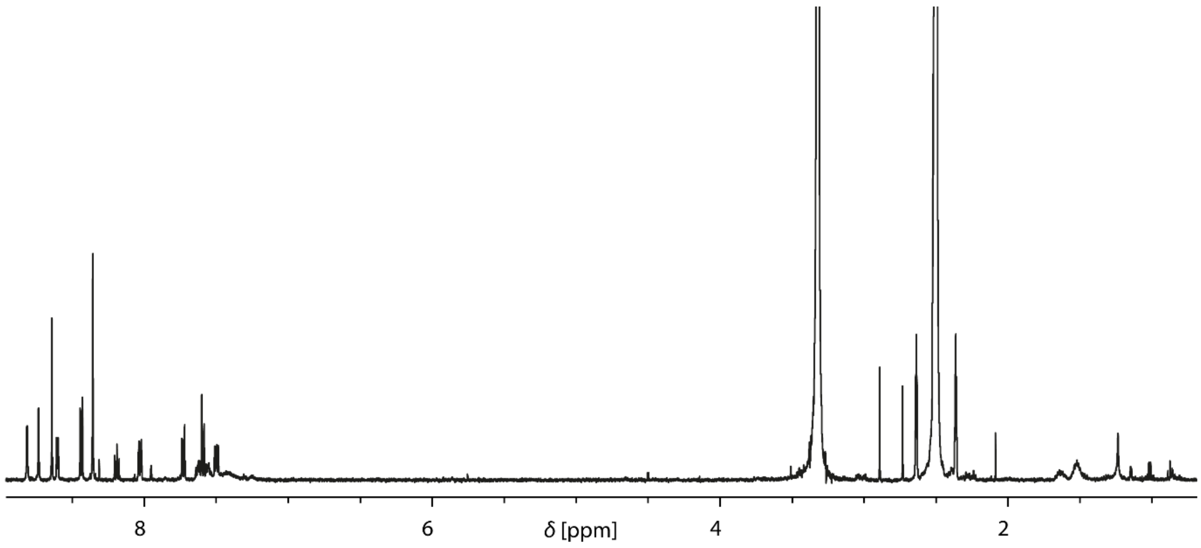


**Figure S25.** $^1$H NMR (500 MHz, 298 K, DMSO-d$_6$) of $\mathbf{L^{pyrenyl}}$.

## 1.5.2 Cage formation of $[Pd_2L^{pyrenyl}{}_4]^{4+}$

To form $[Pd_2\mathbf{L^{pyrenyl}}{}_4]^{4+}$, the procedure described in 1.2.2 was followed.

**$^1$H NMR (500 MHz, 298 K, $CD_3CN$) of** $[Pd_2\mathbf{L^{pyrenyl}}{}_4]^{4+}$**:** *δ* [ppm] = 9.06 (s, 8H, a), 8.79 (d, *J* = 5.5 Hz, 8H, b), 8.70 (s, 8H, e), 8.42 (s, 8H, h), 8.39 (d, *J* = 7.7 Hz, 8H, i), 8.30 (d, *J* = 9.0 Hz, 8H, j), 8.22 (m, 16H, d, k), 8.17 (t, *J* = 7.6 Hz, 4H, l), 7.74 (d, *J* = 8.7 Hz, 8H, f), 7.64 (dd, $J_1$ = 7.8 Hz, $J_2$ = 4.8 Hz, 2H, c), 7.58 (d, *J* = 8.6 Hz, 8H, g).

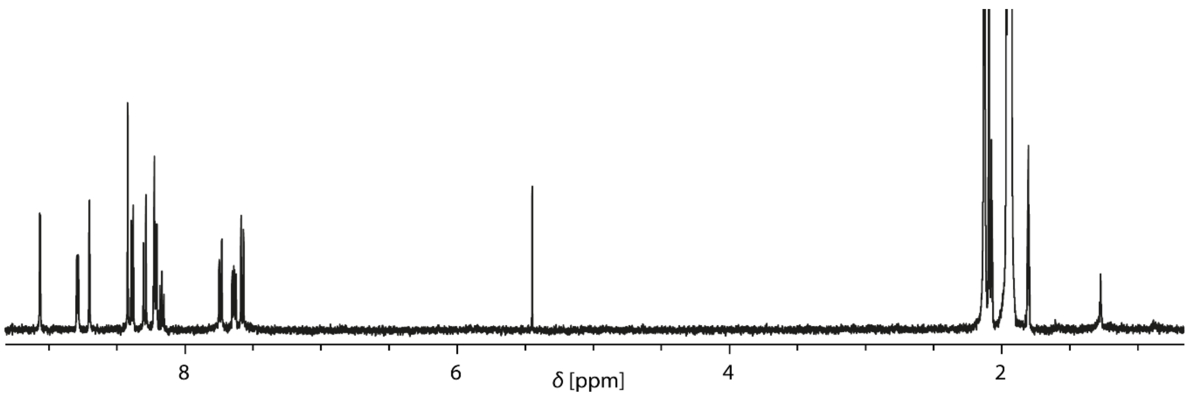


**Figure S26.** $^1$H NMR (500 MHz, 298 K, $CD_3CN$) of $[Pd_2\mathbf{L}^{pyrenyl}{}_4]^{4+}$.

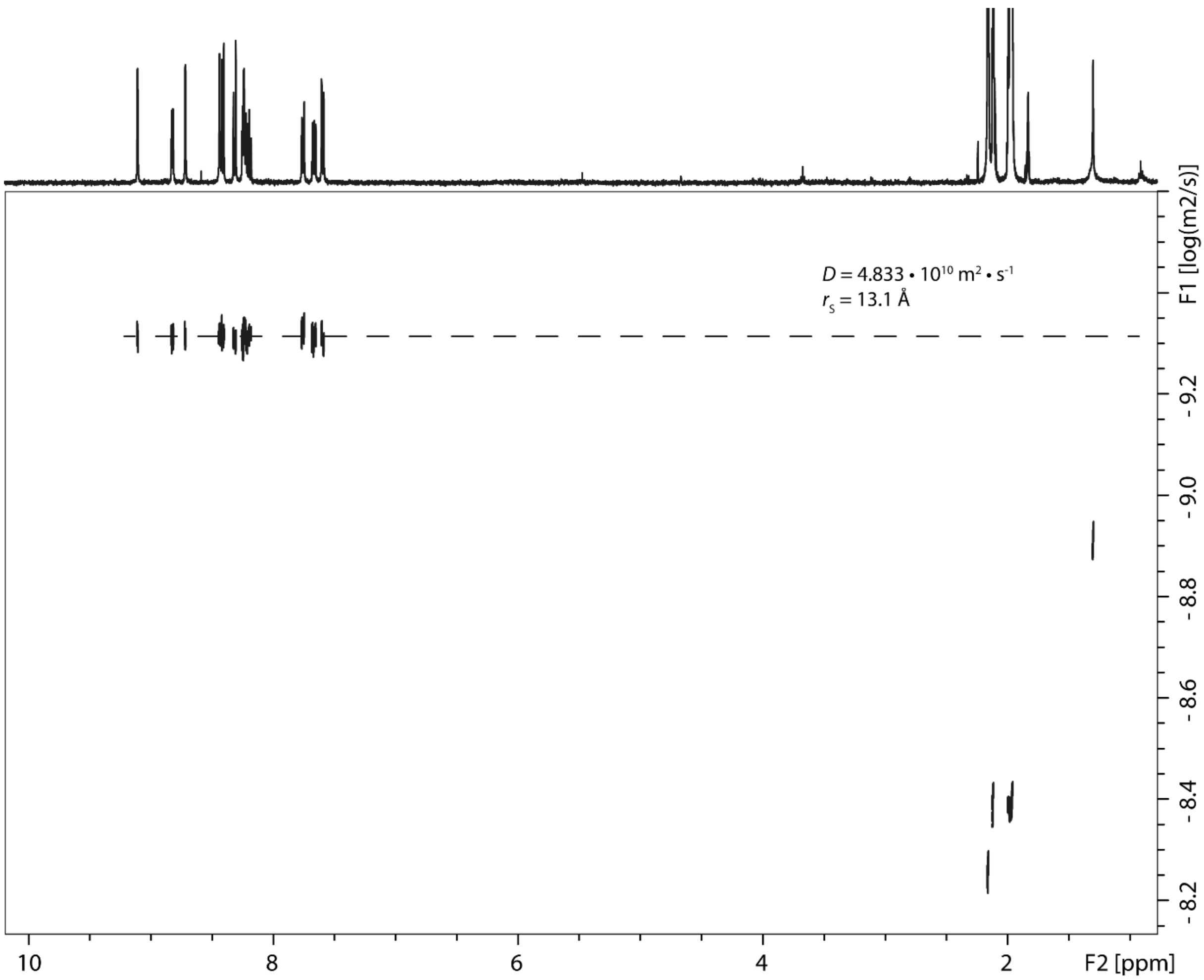


**Figure S27.** $^1$H DOSY NMR (500 MHz, 298 K, $CD_3CN$) of $[Pd_2\mathbf{L}^{pyrenyl}{}_4]^{4+}$.

### 1.5.3 ESI MS and TIMS spectra of $[Pd_2\mathbf{L}^{pyrenyl}_4]^{4+}$

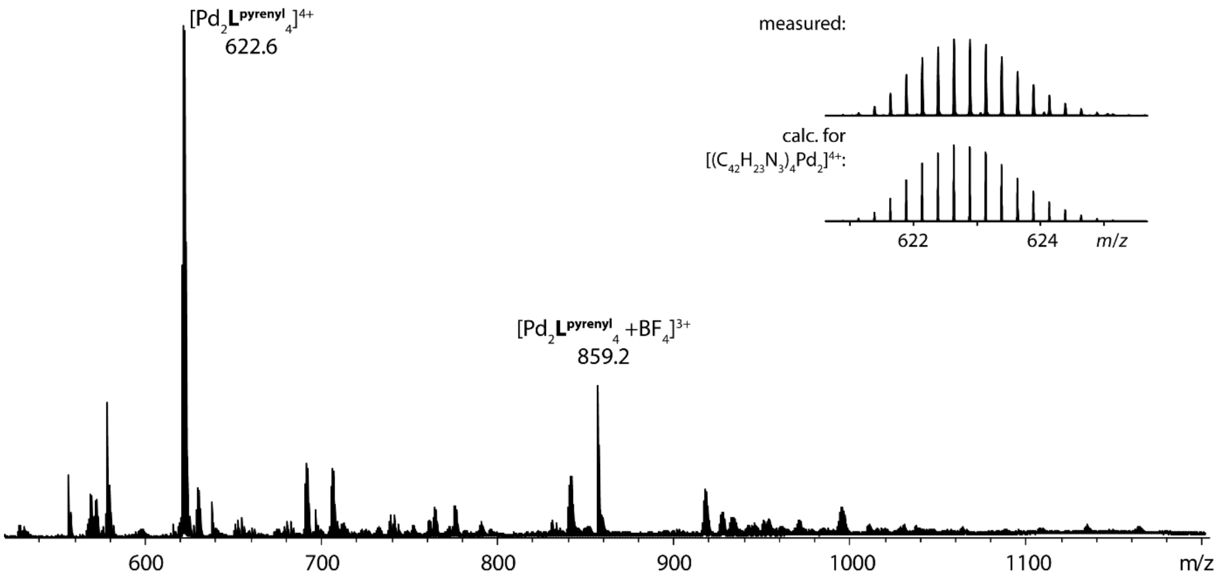


**Figure S28.** ESI MS spectrum of $[Pd_2\mathbf{L}^{pyrenyl}_4](BF_4)_4$.

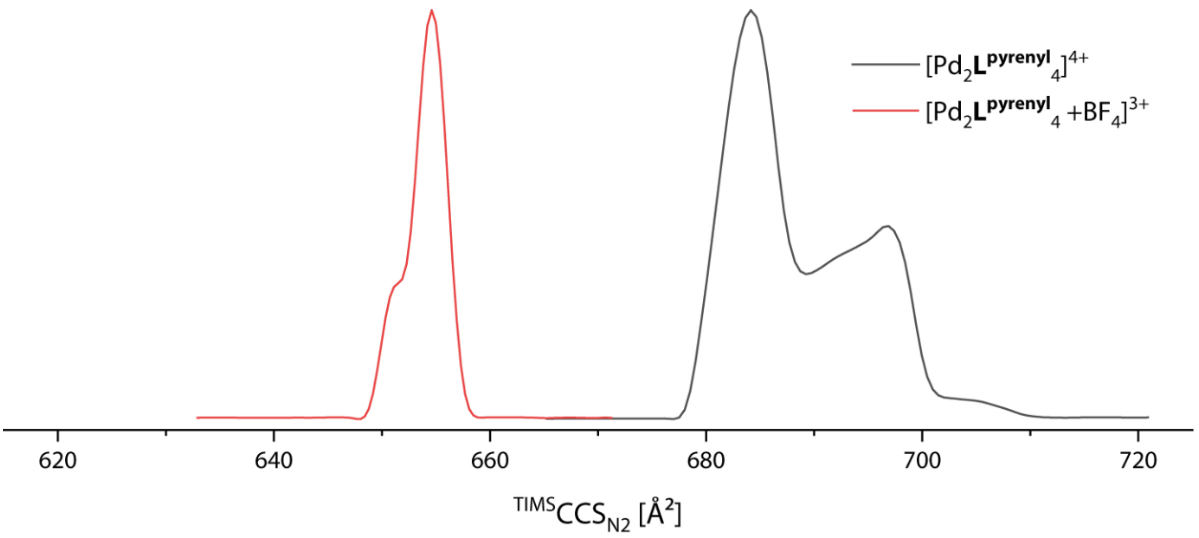


**Figure S29.** Overlaid TIMS mobilograms of $[Pd_2\mathbf{L}^{pyrenyl}_4](BF_4)_4$. Inverse mobilities were normalized and transformed into CCS values.

## 1.6 Single crystal X-ray diffraction analysis

For the crystallographic analysis of [$Pd_2$**L$^{pyrenyl}$**$_4$]($BF_4$)$_4$ single crystals were grown by slow diffusion of 3 ml diisopropyl ether into 150 µl of a 0.2 mM cage solution in nitromethane over two weeks. The small, thin plate shaped colorless crystals were pipetted from mother liquor onto a glass slide containing NVH oil. To avoid cracking of the crystal, it was quickly mounted onto a 0.06 mm nylon loop and immediately flash cooled in liquid nitrogen. Crystals were stored at cryogenic temperature in dry shippers, in which they were safely transported to macromolecular beamline P11 at Petra III,[10] DESY, Germany. The data was collected at 100(2) K on an Eiger 2X 16M detector using a high precision 1-axis goniostat equipped with a Stäubli sample changing robot. 3600 diffraction images were collected in a 360° ϕ sweep at a chosen wavelength of λ = 0.88561 Å (14KeV) using double crystal monochromator (Si-111 and Si-113 reflections) and a detector distance of 154 mm, 100 % filter transmission, 0.1° step width and 0.2 seconds exposure time per image. Data integration and reduction were undertaken using XDS[11]. The data was cut at 1.0 Å using a mean I/sig(I) > 1 as cutoff criterium. The structure was solved by direct methods using SHELXT 2018/2[12] and refined by full-matrix least-squares methods against $F^2$ by SHELXL-2014/7.[13] All non-hydrogen atoms were refined with anisotropic displacement parameters using 22 CPU cores for full-matrix least-squares routines on F2 and ShelXle[14] as a graphical user interface and the DSR program plugin was employed for modeling.[15,16]
Stereochemical restraints for the ligands **L$^{pyrenyl}$** (residue CPP) were generated by the GRADE program using the GRADE Web Server (http://grade.globalphasing.org) and applied in the refinement. A GRADE dictionary for SHELXL contains target values and standard deviations for 1,2-distances (DFIX) and 1,3-distances (DANG), as well as restraints for planar groups (FLAT). All displacements for non-hydrogen atoms were refined anisotropically. The refinement of ADP's for carbon, nitrogen and oxygen atoms was enabled by a combination of similarity restraints (SIMU) and rigid bond restraints (RIGU).14 The contribution of the electron density from disordered counterions and solvent molecules, which could not be modeled with discrete atomic positions were handled using the SQUEEZE[17] routine in PLATON.[18] The solvent mask file (.fab), computed by PLATON, was included in the SHELXL refinement via the ABIN instruction leaving the measured intensities untouched.
The diffractometer was equipped with a low temperature device and used synchrotron. The hydrogen atoms were refined isotropically on calculated positions using a riding model with their $U_{iso}$ values constrained to 1.5 times the $U_{eq}$ of their pivot atoms for terminal sp$^3$ carbon atoms and 1.2 times for all other carbon atoms. Crystallographic data (including structure factors) for the structures reported in this paper have been deposited with the Cambridge Crystallographic Data Centre. CCDC no. 2253145 contain the supplementary crystallographic data for this paper. Copies of the data can be obtained free of charge from The Cambridge Crystallographic Data Centre via www.ccdc.cam.ac.uk/structures.

**Table S2.** Crystal data and structure refinement for [$Pd_2$**L**$^{pyrenyl}$$_4$]($BF_4$)$_4$, cd248d.

| | |
|---|---|
| Compound | [$Pd_2$**L**$^{pyrenyl}$$_4$]($BF_4$)$_4$ |
| CIF ID | cd248d |
| CCDC number | 2253145 |
| Empirical formula | $C_{168}H_{92}B_{3.50}F_{14}N_{12}Pd_2$ |
| Formula weight | 2795.17 |
| Temperature [K] | 100(2) |
| Crystal system | monoclinic |
| Space group (number) | $P2_1/c$ (14) |
| *a* [Å] | 25.58(3) |
| *b* [Å] | 37.13(3) |
| *c* [Å] | 38.88(4) |
| α [Å] | 90 |
| β [Å] | 105.151(12) |
| γ [Å] | 90 |
| Volume [$Å^3$] | 35649(58) |
| *Z* | 8 |
| $\rho_{calc}$ [$g/cm^3$] | 1.042 |
| $\mu$ [$mm^{-1}$] | 0.461 |
| *F*(000) | 11356 |
| Crystal size [$mm^3$] | 0.040×0.040×0.005 |
| Crystal color | colorless |
| Crystal shape | plate |
| Radiation | synchrotron (λ=0.88561 Å) |
| 2Θ range [°] | 1.92 to 43.30 (1.20 Å) |
| Index ranges | $-21 \le h \le 21$<br>$-30 \le k \le 30$<br>$-32 \le l \le 32$ |
| Reflections collected | 144173 |
| Independent reflections | 21294<br>$R_{int}$ = 0.0739<br>$R_{sigma}$ = 0.0412 |
| Completeness to θ = 21.650° | 98.6 % |
| Data / Restraints / Parameters | 21294/8052/3592 |
| Goodness-of-fit on $F^2$ | 1.678 |
| Final *R* indexes<br>[$I \geq 2\sigma(I)$] | $R_1$ = 0.1520<br>$wR_2$ = 0.4177 |
| Final *R* indexes<br>[all data] | $R_1$ = 0.1964<br>$wR_2$ = 0.4491 |
| Largest peak/hole [$eÅ^3$] | 1.46/-0.68 |

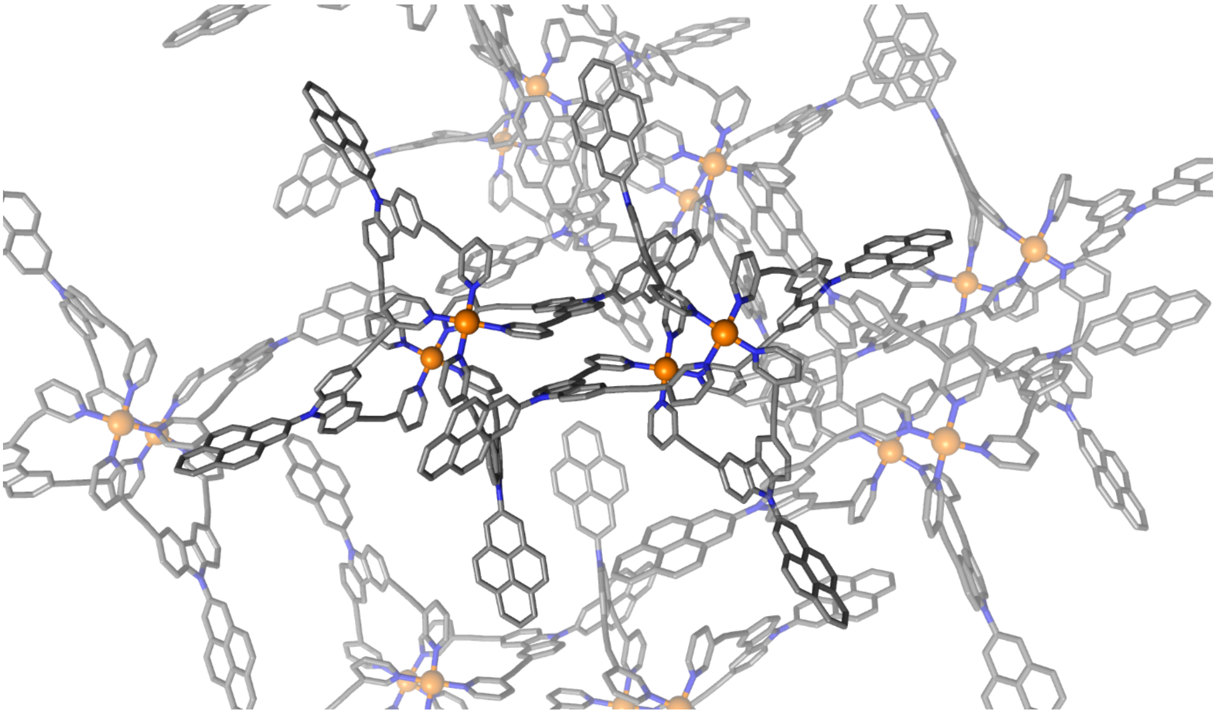

**Figure S30.** Crystal packing of the obtained X-ray structure of $[Pd_2\mathbf{L}^{pyrenyl}{}_4](BF_4)_4$. Counter anions, hydrogen atoms and solvent molecules omitted for clarity.

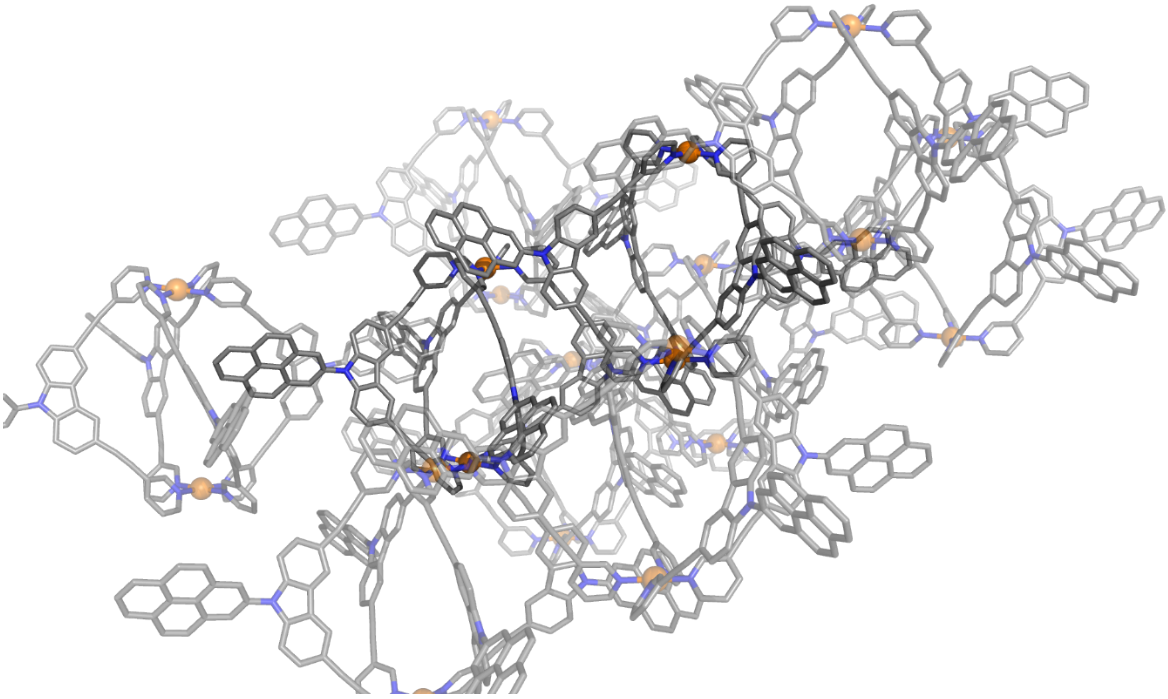

**Figure S31.** Crystal packing of the obtained X-ray structure of $[Pd_2\mathbf{L}^{pyrenyl}{}_4](BF_4)_4$. Counter anions, hydrogen atoms and solvent molecules omitted for clarity.

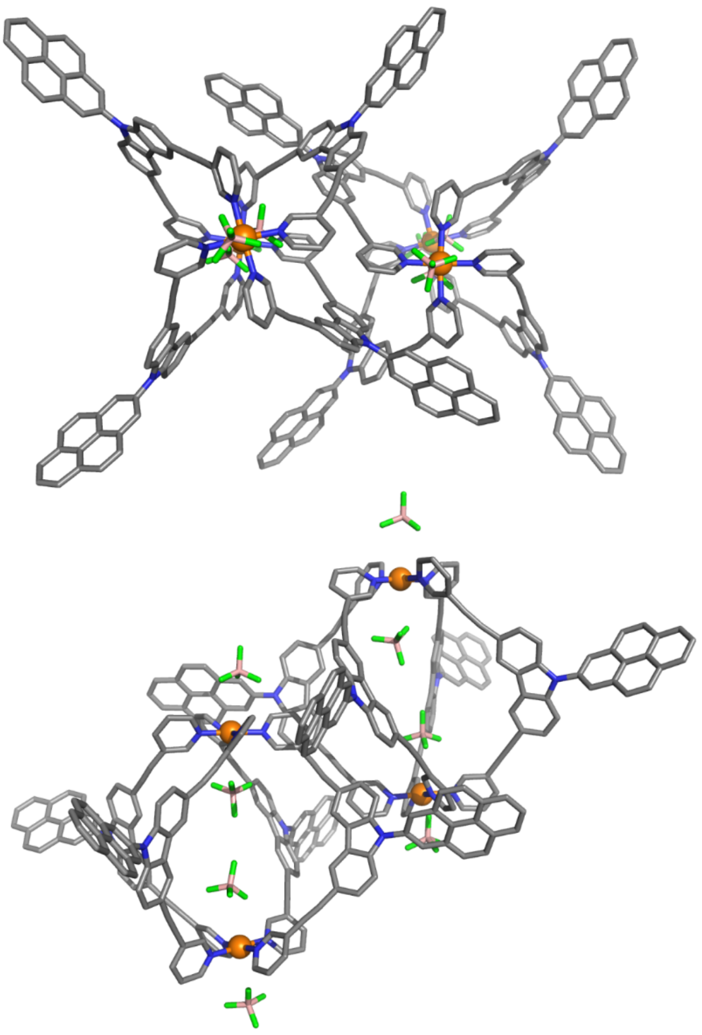

**Figure S32.** Top and side view of asymmetric unit cell of the obtained X-ray structure of $[Pd_2\mathbf{L}^{pyrenyl}{}_4](BF_4)_4$. Hydrogen atoms and solvent molecules omitted for clarity.

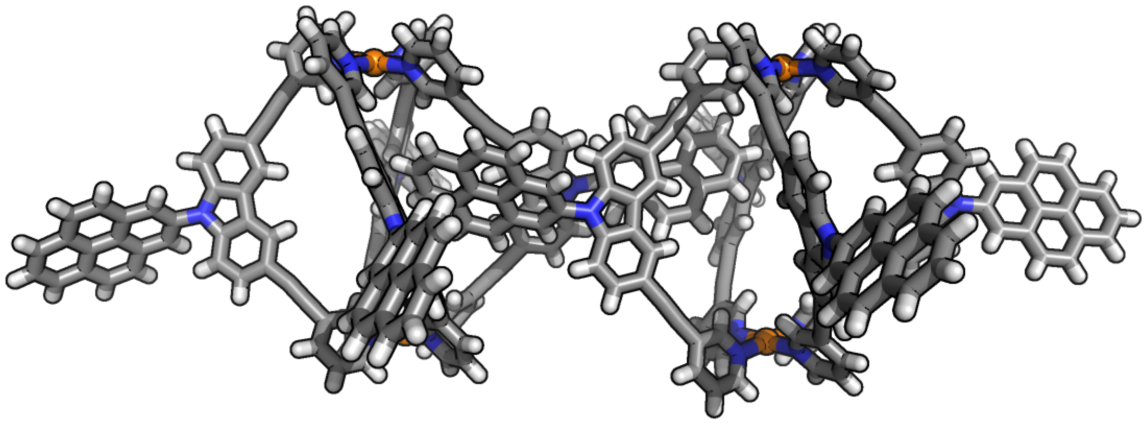

**Figure S33.** Two cages in the crystal packing of $[Pd_2\mathbf{L}^{pyrenyl}{}_4](BF_4)_4$ showing inter-cage π stacking. Solvent molecules and counter anions omitted for clarity.

# 2. Computational section

## 2.1 Molecular Dynamics Simulations with GFN2-xTB

The MD simulations were done with the xtb software, version 6.4.1,[19] using the default GFN2-xTB method.[20] We used the --omd flag, meaning that the run starts with a normal optimization. The temperature was set to 303 K for initial gas phase MDs. The Pd–N–$C_{para}$ angle was always constrained to 180° using the default force constant because it was observed that this angle is too flexible according to GFN2-xTB, see section 2.4.2. The simulation time was 1000 ps. Other than that, and if not stated otherwise, always default settings were used. To obtain starting structures in which the side chains are already partially backfolded, preliminary MDs for about 500 ps with the same settings were conducted starting from structures with perfectly stretched out side chains.

## 2.2 $^{theo}CCS_{N2}$ calculation

As mentioned in the main text, we focus on the ESI MS detected species without $BF_4^-$ counter anions in this study because of the increasing positive deviation in the $^{theo}CCS_{N2}$ calculation with increasing amount of $BF_4^-$. For a source of this error any anisotropic alignment of the system in the TIMS tunnel can be ruled out because of the relatively weak electric field applied. Collidoscope does not have LJ parameters for fluorine, so we obtained them ourselves (see Section 2.2.4 Optimization of Fluorine Lennard-Jones parameters for Collidoscope, $\varepsilon_F$ = 500 Å, $\sigma_F$ = 2.67 J/mol), which slightly improve but do not dissolve the error. Different Trajectory Method programs with different parameters show the similar error even with different point charge schemes, see below. We therefore believe this to be a scattering issue as the $BF_4^-$ is non-covalently bound and could bounce back upon collision with $N_2$, leading to a larger scattering angle, which is not possible to capture in the Trajectory Method because the cage and the anions are considered fixed. Details of the parametrization and experimental and theoretical (also from different programs) values of species with one or two $BF_4^-$ counter anions are found in the next few sections.

### 2.2.1 Collidoscope, general settings

The theoretical collisional cross sections ($^{theo}CCS_{N2}$) were calculated using the Collidoscope software, version 1.4, from Prell et al.[21] The temperature was set to 303 K, however, the exact temperature during the measurement cannot be determined precisely. Ion-quadrupole interactions were kept disabled. The number of energy states was increased from 4 to 16, resulting in about 500,000 trajectories. The CM5 point charges were calculated with GFN1-xTB, using the xtb software, version 6.4.1, from Grimme et al.[22,23] Alternatively, Mulliken charges were used. These were also calculated with GFN1-xTB. As Collidoscope does not have Lennard-Jones parameters for B, F and Pd we used the parameters from C for the B and Pd atoms (both rather buried in the structures of the cages and counter anions) and parameterized F (more exposed) ourselves.

### 2.2.2 HPCCS, general settings

For the calculation of $^{theo}CCS_{N2}$ values with the HPCCS software,[24] version 1.0 was used. The same CM5 point charges used for calculations with Collidoscope were also used here. The content of the config.in file was

```
1    500    20    50    1000    303    2
```

Meaning 500,000 trajectories, 303 K and $N_2$ as inert gas. For Pd and B the LJ-parameters from C were used. Other than that, default settings were used.

### 2.2.3 IMoS, general settings

For the calculation of $^{theo}CCS_{N2}$ values with the IMoS software,[25] version 10c ("IMoS110cL64LJ") was used. The same CM5 point charges used for calculations with Collidoscope were also used here. The Trajectory Method with the normal 12-6-4 potential, 500,000 trajectories, 303 K and $N_2$ as inert gas was chosen. For Pd and B the LJ-parameters from C were used. Other than that, default settings were used.

### 2.2.4 Optimization of Fluorine Lennard-Jones parameters for Collidoscope

We found in the literature several measurements of drug-sized molecules containing at least one fluorine atom: Dexamethasone,[26] Leflunomide,[27] Flufenamic Acid,[27] Celecoxib,[28] and Sulfentrazone.[28] All species were single protonated and would have been uncharged otherwise. The ions were modeled and optimized with r$^2$SCAN-3c.[29] Following the procedure described by Larriba et al., we varied the parameters $\varepsilon_F$ (from 0 J/mol to 1500 J/mol in 50 J/mol steps) and $\sigma_F$ (from 1 to 4 Å in 0.125 Å steps) and calculated $F_{opt}$ each as defined by Larriba et al.[30] The according 3D plot is shown in Figure S33. As can be seen a valley of local minima is obtained. We chose $\varepsilon_F$ = 500 J/mol randomly and minimized $\sigma_F$ with a 0.01 Å step size, resulting in 2.67 Å.

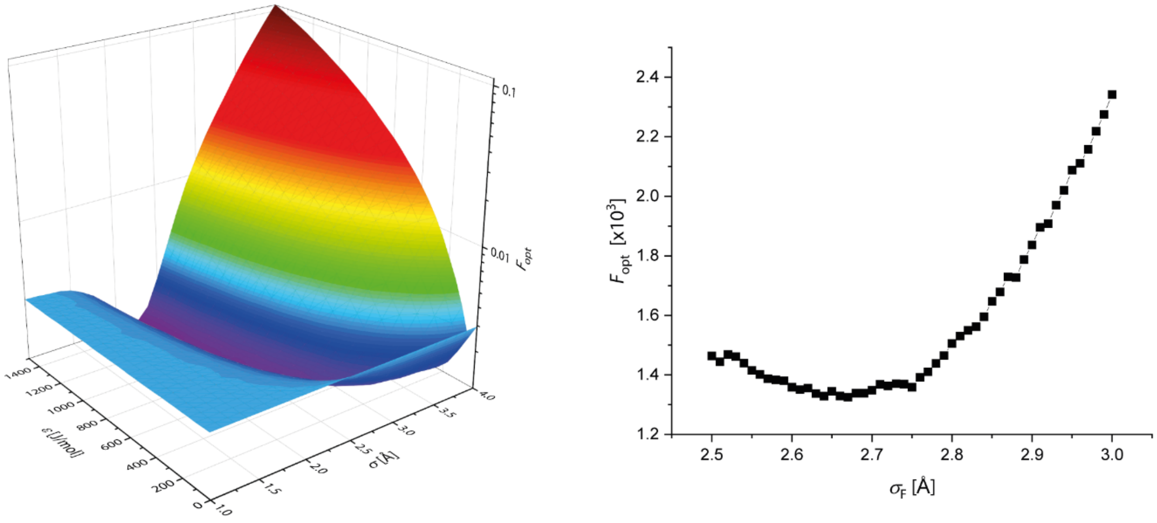


**Figure S34.** Left: Plot of $F_{opt}$ against $\varepsilon_F$ and $\sigma_F$. Right: Plot of $F_{opt}$ against and $\sigma_F$, keeping $\varepsilon_F$ constant at 500 J/mol.

We validated the results with the training set and with a test set consisting of $[Pd_2\mathbf{L}^{\mathbf{small}}_4\text{+}BF_4]^{3+}$ and $[Pd_2\mathbf{L}^{\mathbf{small}}_4\text{+}2BF_4]^{2+}$ were $\mathbf{L}^{\mathbf{small}}$ is a short bis-monodentate ligand shown in Scheme S9, as well as the free, single-protonated ligand **o-L**$^{\mathbf{DTE}}$ and the 4+, 3+ and 2+ species of the resulting $Pd_2$**o-L**$^{\mathbf{DTE}}_4$ cage. **o-L**$^{\mathbf{DTE}}$ is another banana-shaped bis-monodentate ligand with pyridine donor groups and alkyne spacers.[31] The backbone of **o-L**$^{\mathbf{DTE}}$ is a dithienyl-hexafluoropentenyl group and we are here only considering the open-ring form. As can be seen in Table S2, not only the theoretical CCS values for the training set but also for the test set greatly improved showing that the new parameters are acceptable.

**Table S3.** Experimental and theoretical CCS values and relative deviations for various species containing fluorine. Values using the LJ parameters for fluorine from oxygen are compared to values with optimized LJ parameters.

| Species | $^{TIMS}CCS_{N2}$ | $^{theo}CCS_{N2}(\varepsilon_F=\varepsilon_O,\ \sigma_F=\sigma_O)$ | | $^{theo}CCS_{N2}(\varepsilon_F=500$ kJ/mol, $\sigma_F=2.67$ Å) | |
|---|---|---|---|---|---|
| | [Å$^2$] | $^{theo}CCS_{N2}$ [Å$^2$] | $\Delta_\%$ | $^{theo}CCS_{N2}$ [Å$^2$] | $\Delta_\%$ |
| [Celecoxib +H]$^+$ | 186.12 | 198.0 | +6.4 % | 185.1 | –0.6 % |
| [Dexamethasone +H]$^+$ | 190.7 | 188.8 | –1.0 % | 188.4 | –1.2 % |
| [Flufenamic acid +H]$^+$ | 156.1 | 170.1 | +9.0 % | 158.6 | +1.6 % |
| [Leflunomide +H]$^+$ | 157.3 | 161.7 | +2.8 % | 151.9 | –3.4 % |
| [Sulfentrazone +H]$^+$ | 173.63 | 182.4 | +5.1 % | 173.6 | +0.0 % |
| $[Pd_2\mathbf{L}^{\mathbf{small}}{}_4]^{4+}$ | 503 ± 3 | 498.6 | –0.9 % | 498.6 | –0.9 % |
| $[Pd_2\mathbf{L}^{\mathbf{small}}{}_4+BF_4]^{3+}$ | 452 ± 2 | 463.0 | +2.4 % | 459.2 | +1.6 % |
| $[Pd_2\mathbf{L}^{\mathbf{small}}{}_4+2BF_4]^{2+}$ | 430 ± 1 | 456.8 | +6.1 % | 448.8 | +4.3 % |
| [**o-L**$^{\mathbf{DTE}}$ +H]$^+$ | 227 ± 2 | 256.0 | +13.0 % | 241.1 | +6.4 % |
| $[Pd_2\mathbf{o\text{-}L}^{\mathbf{DTE}}{}_4]^{4+}$ | 600 ± 6 | 638.7 | +6.4 % | 608.0 | +1.3 % |
| $[Pd_2\mathbf{o\text{-}L}^{\mathbf{DTE}}{}_4+BF_4]^{3+}$ | 562 ± 5 | 610.4 | +8.7 % | 577.8 | +2.9 % |
| $[Pd_2\mathbf{o\text{-}L}^{\mathbf{DTE}}{}_4+2BF_4]^{2+}$ | 531 ± 3 | 591.6 | +11.4 % | 554.7 | +4.5 % |

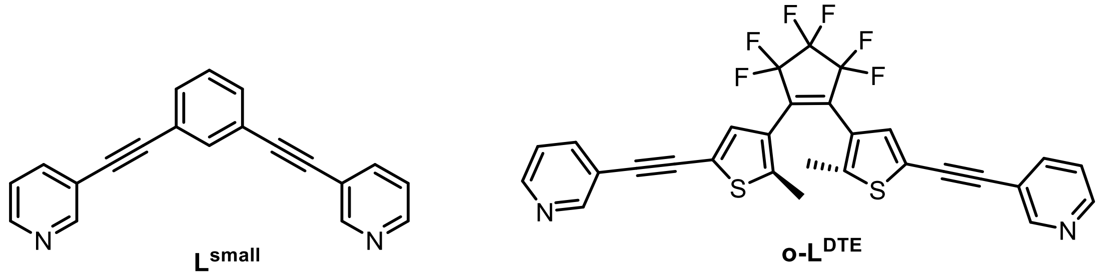


**Scheme S9.** Ligands **L**$^{\mathbf{small}}$ and **o-L**$^{\mathbf{DTE}}$ used to form $Pd_2\mathbf{L}_4$ assemblies for verification of new LJ parameters for fluorine.

To investigate whether the two counter anions are most likely inside or one inside and one outside for the species $[Pd_2\mathbf{L}^{\mathbf{small}}{}_4 +2BF_4]^{2+}$, $[Pd_2\mathbf{L}^{\mathbf{methyl}}{}_4 +2BF_4]^{2+}$ and $[Pd_2\mathbf{o\text{-}L}^{\mathbf{DTE}}{}_4 +2BF_4]^{2+}$, single point energy differences between inside–inside and inside–outside bound configurations on ωB97M-D4/def2-TZVP level were calculated. Additionally, the Pd–Pd distances from the optimized inside–inside model were measured. Apparently, for the small cage the inside–outside version is favored by 10.5 kJ/mol, simply because the Pd-Pd distance is only 11.6 Å, causing a very small distance between the two $BF_4^-$. For the methyl carbazole cage, representative for all carbazole mono cages, the inside-outside version is still favored, even though not as much (6.1 kJ/mol difference, Pd–Pd distance: 13.3 Å). For the large open DTE cage, the inside–inside version is clearly favored by 14.3 kJ/mol due to the high Pd–Pd distance of 17.0 Å.

## 2.2.5 $^{theo}CCS_{N2}$ calculation using different Trajectory Method software

**Table S4.** $^{TIMS}CCS_{N2}$ values of $[Pd_2\mathbf{L}^{methyl}_4]^{4+}$, $[Pd_2\mathbf{L}^{methyl}_4{+}BF_4]^{3+}$ and $[Pd_2\mathbf{L}^{methyl}_4{+}2BF_4]^{2+}$ and the according $^{theo}CCS_{N2}$ values using either Collidoscope, Collidoscope with Mulliken instead of CM5 point charges, HPCCS or IMoS and the relative deviations to the $^{TIMS}CCS_{N2}$ values. Unit is Å².

| Species | $^{TIMS}CCS_{N2}$ | Collidoscope | | HPCCS | | IMoS | | Collidoscope, Mulliken | |
|---|---|---|---|---|---|---|---|---|---|
| $[Pd_2\mathbf{L}^{methyl}_4]^{4+}$ | 582 ± 3 | 596.8 | +2.5 % | 623.8 | +7.2 % | 615.8 | +5.8 % | 600.3 | +3.1 % |
| $[Pd_2\mathbf{L}^{methyl}_4 +BF_4]^{3+}$ | 542 ± 2 | 563.8 | +4.1 % | 592.6 | +9.4 % | 588.9 | +8.7 % | 565.9 | +4.5 % |
| $[Pd_2\mathbf{L}^{methyl}_4 +2BF_4]^{2+}$ | 520 ± 2 | 554.4 | +6.7 % | 586.0 | +12.8 % | 581.6 | +11.9 % | 555.8 | +7.0 % |

## 2.2.6 $^{theo}CCS_{N2}$ calculation of snapshots from the trajectories

To obtain $^{theo}CCS_{N2}$ values averaged over time, snapshots of the trajectory file for every picosecond (actually only every 24/25th of a picosecond, because the time step used was 4 fs) were extracted and used for CCS calculation without further geometry optimization. The plots of the resulting $^{theo}CCS_{N2}$ and relative deviations to $^{TIMS}CCS_{N2}$ over time are shown in Figures S34 to S37 and the averaged values are listed in Table S4.

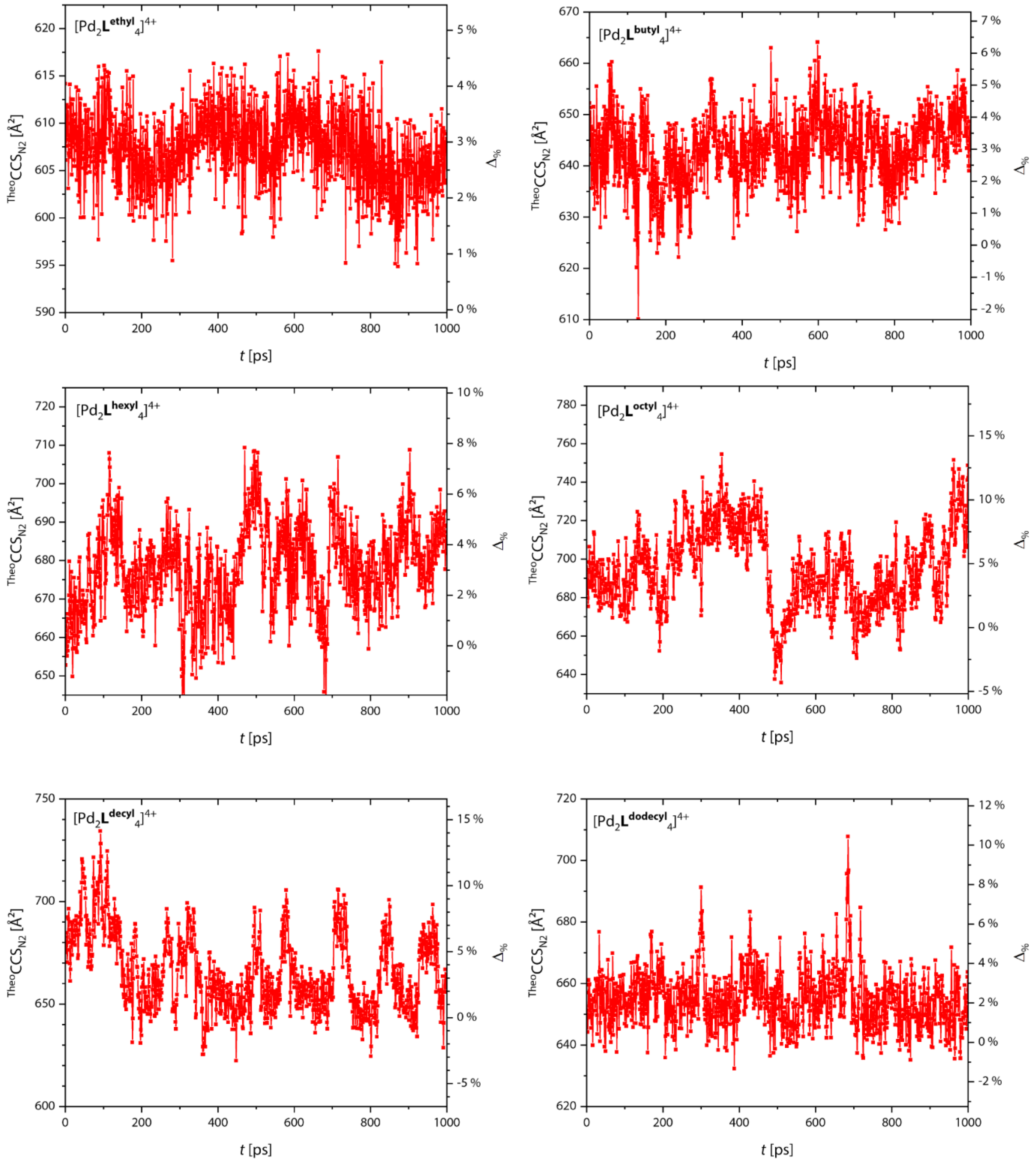


**Figure S35.** $^{theo}CCS_{N2}$ values for $[Pd_2\mathbf{L}^{R}{}_4]^{4+}$ calculated from the snapshots taken every picosecond from the MD simulations plotted against time. Right y-axis shows the relative deviation to the experimental CCS ($^{TIMS}CCS_{N2}$).

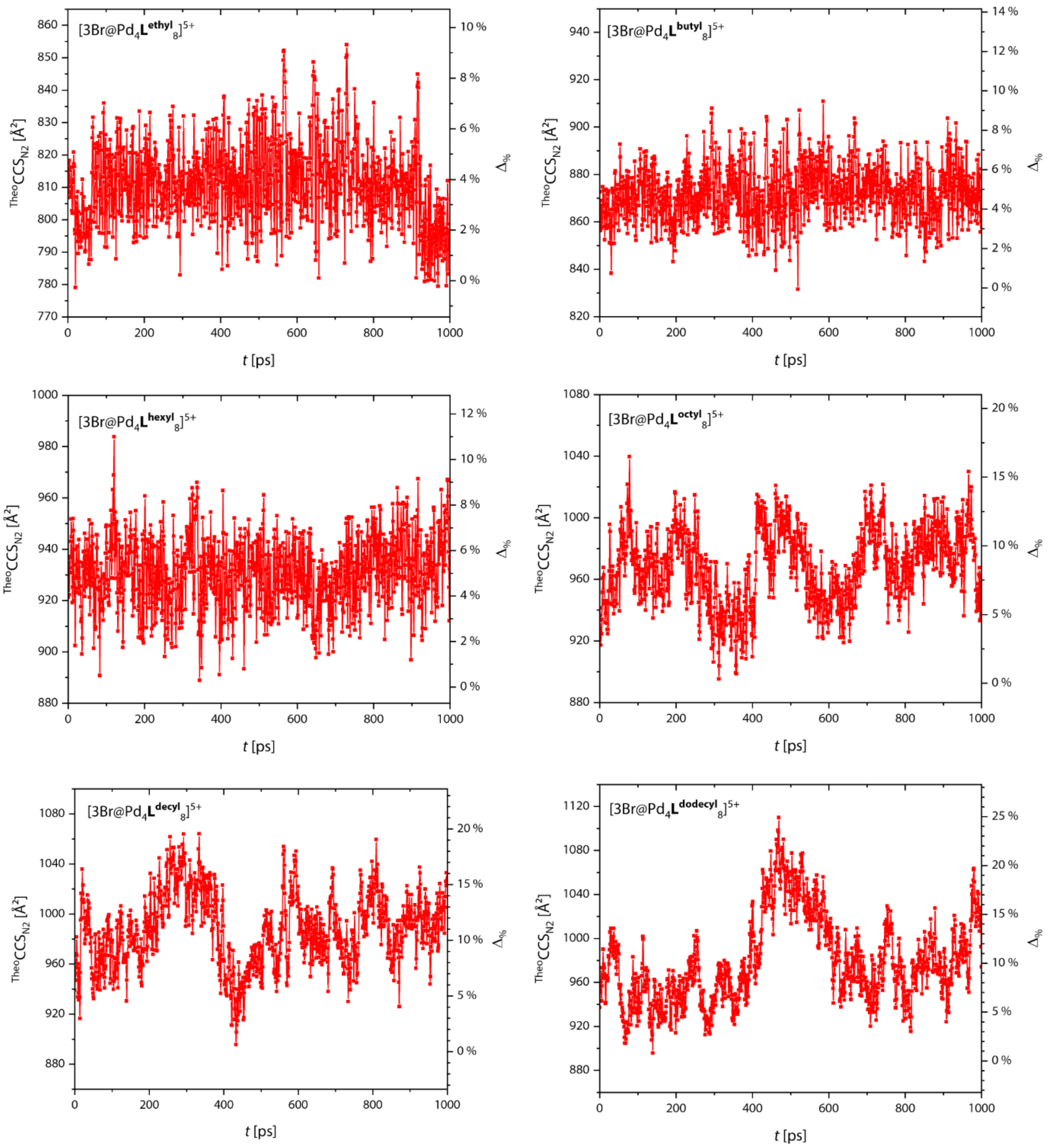


**Figure S36.** ${}^{theo}CCS_{N2}$ values for $[3Br@Pd_4\mathbf{L}^{R}{}_8]^{5+}$ calculated from the snapshots taken every picosecond from the MD simulations plotted against time. Right y-axis shows the relative deviation to the experimental CCS (${}^{TIMS}CCS_{N2}$).

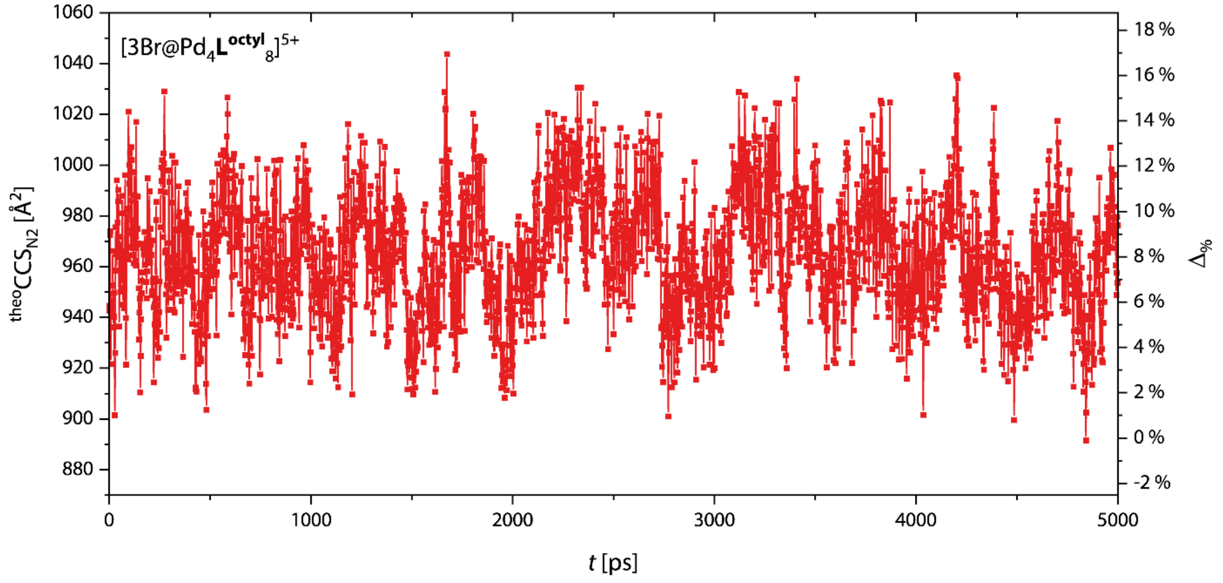


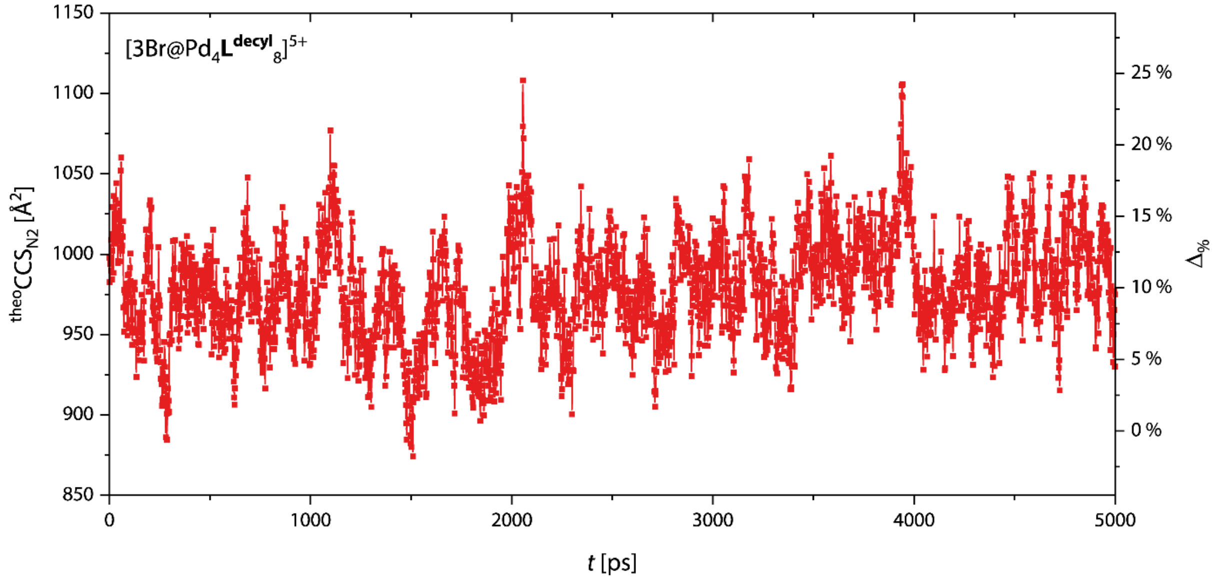


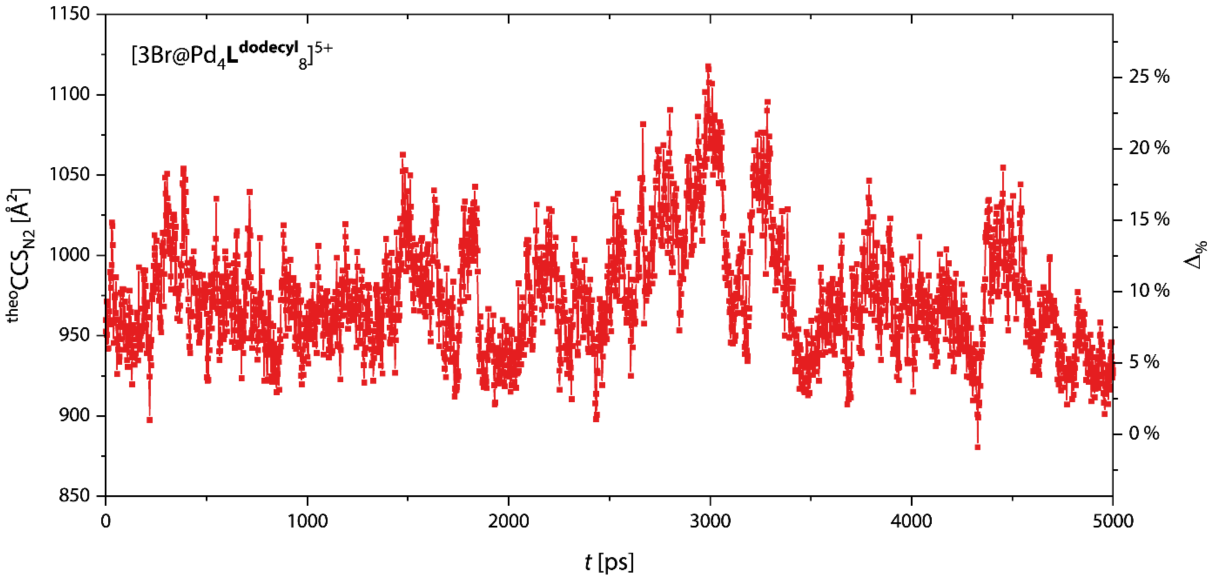


**Figure S37.** ${}^{theo}CCS_{N2}$ values for $[3Br@Pd_4\mathbf{L}^R{}_8]^{5+}$ with **R** being either octyl, decyl or dodecyl calculated from the snapshots taken every picosecond from the longer MD simulations plotted against time. Right y-axis shows the relative deviation to the experimental CCS (${}^{TIMS}CCS_{N2}$).

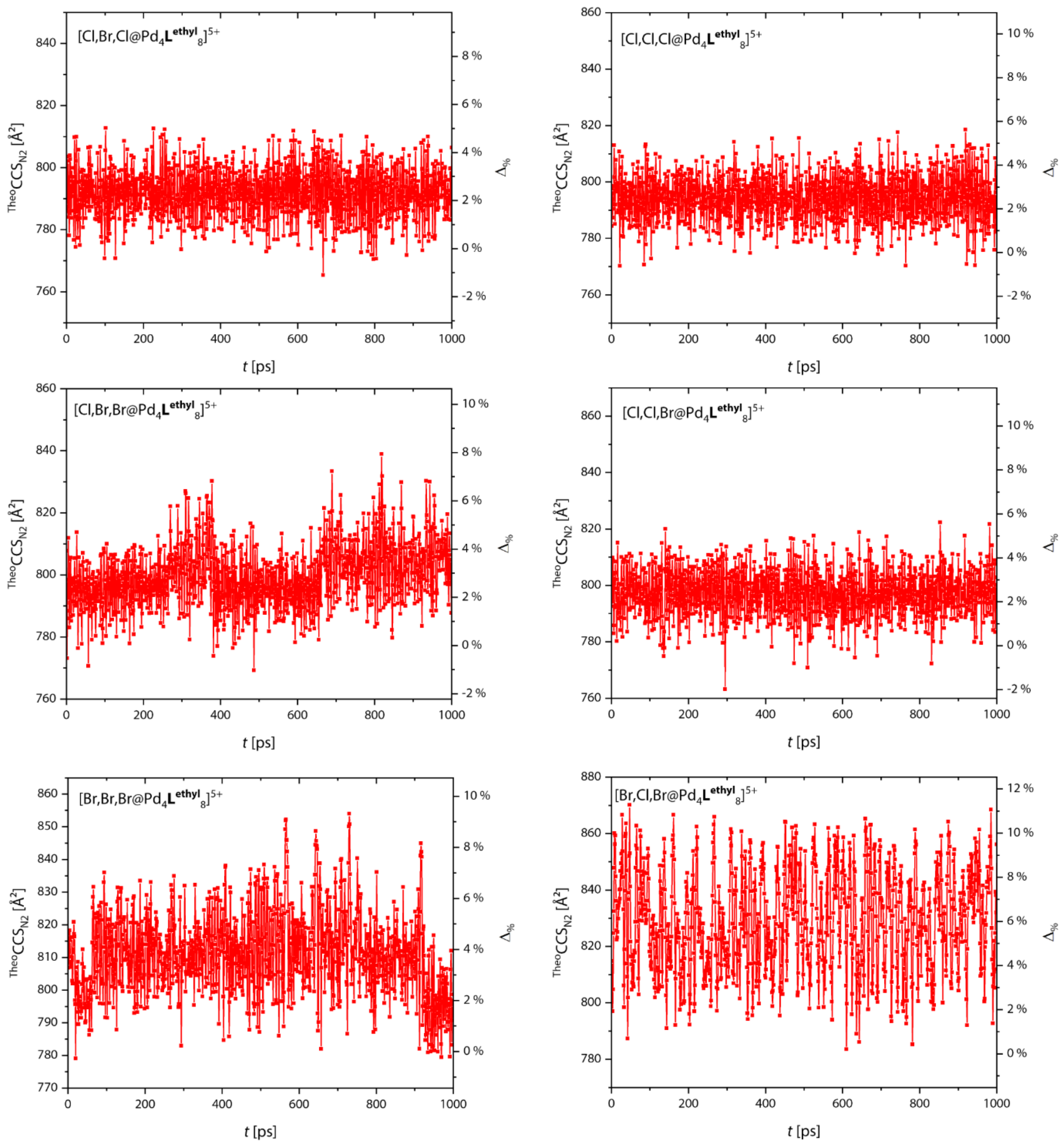


**Figure S38.** $^{theo}CCS_{N2}$ values for $[3X@Pd_4\mathbf{L}^{ethyl}{}_8]^{5+}$ with X being either Cl or Br and calculated from the snapshots taken every picosecond from the MD simulations plotted against time. Right y-axis shows the relative deviation to the experimental CCS ($^{TIMS}CCS_{N2}$).

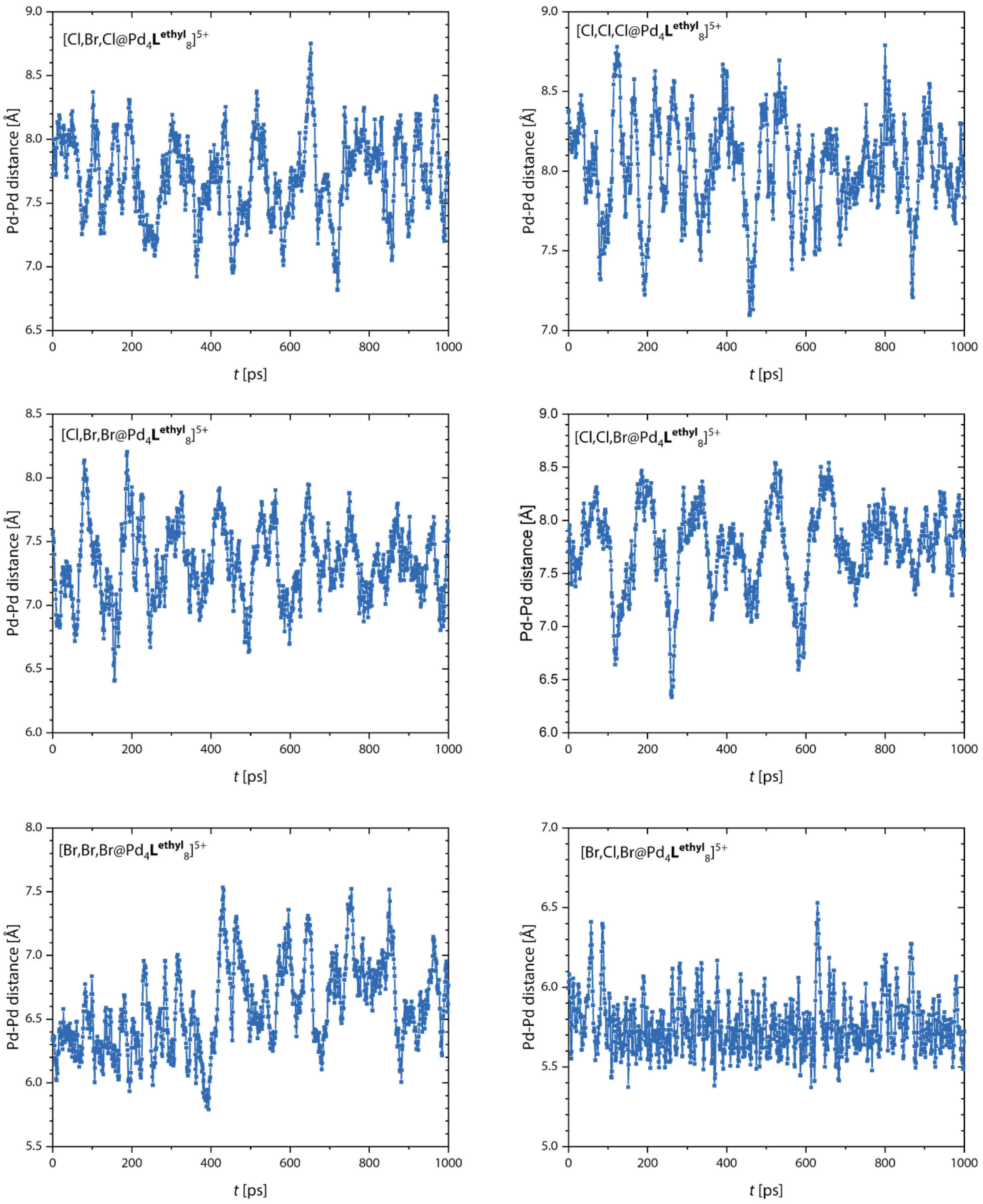


**Figure S39.** Pd-Pd distance of the central cavity in $[3X@Pd_4\mathbf{L}^{ethyl}{}_8]^{5+}$ with X being either Cl or Br plotted against time.

**Table S5.** Experimental and theoretical $CCS_{N2}$ values for $[Pd_2\mathbf{L}^{\mathbf{R}}_4]^{4+}$ and $[3X@Pd_4\mathbf{L}^{\mathbf{R}}_8]^{5+}$ with R = ethyl, butyl, hexyl, octyl, decyl, dodecyl and X = Cl, Br. Experimental errors are the HWHM (half width of half maxima) of the measured signals. For the error determination of the $^{theo}CCS_{N2}$ values, a blocking analysis according to Grossfield and Zuckerman was done.[32]

| System | $^{TIMS}CCS_{N2}$ [Å²] | $^{theo}CCS_{N2}$ [Å²] | $\Delta_{\%}$ |
|---|---|---|---|
| $[Pd_2\mathbf{L}^{\mathbf{ethyl}}_4]^{4+}$ | 590 ± 1 | 606.6 ± 0.6 | +2.9 % |
| $[Pd_2\mathbf{L}^{\mathbf{butyl}}_4]^{4+}$ | 625 ± 2 | 643.3 ± 0.9 | +3.0 % |
| $[Pd_2\mathbf{L}^{\mathbf{hexyl}}_4]^{4+}$ | 658 ± 3 | 678 ± 2 | +3.1 % |
| $[Pd_2\mathbf{L}^{\mathbf{octyl}}_4]^{4+}$ | 664 ± 2 | 695 ± 4 | +4.7 % |
| $[Pd_2\mathbf{L}^{\mathbf{decyl}}_4]^{4+}$ | 643 ± 3 | 666 ± 3 | +3.5 % |
| $[Pd_2\mathbf{L}^{\mathbf{dodecyl}}_4]^{4+}$ | 641 ± 2 | 655 ± 1 | +2.2 % |
| $[3Cl@Pd_4\mathbf{L}^{\mathbf{ethyl}}_8]^{5+}$ | 775 ± 2 | 794.1 ± 0.1 | +2.5 % |
| $[Cl,Br,Cl@Pd_4\mathbf{L}^{\mathbf{ethyl}}_8]^{5+}$ | 774 ± 1 | 792.0 ± 0.2 | +2.3 % |
| $[Cl,Cl,Br@Pd_4\mathbf{L}^{\mathbf{ethyl}}_8]^{5+}$ | 777 ± 2 | 797.1 ± 0.2 | +2.4 % |
| $[Br,Cl,Br@Pd_4\mathbf{L}^{\mathbf{ethyl}}_8]^{5+}$ | 782 ± 3 | 833 ± 1 | +6.6 % |
| $[Cl,Br,Br@Pd_4\mathbf{L}^{\mathbf{ethyl}}_8]^{5+}$ | 779 ± 1 | 803 ± 1 | +3.3 % |
| $[3Br@Pd_4\mathbf{L}^{\mathbf{ethyl}}_8]^{5+}$ | 781 ± 1 | 813 ± 1 | +3.8 % |
| $[3Br@Pd_4\mathbf{L}^{\mathbf{butyl}}_8]^{5+}$ | 832 ± 3 | 871.9 ± 0.9 | +4.8 % |
| $[3Br@Pd_4\mathbf{L}^{\mathbf{hexyl}}_8]^{5+}$ | 886 ± 3 | 930 ± 1 | +5.0 % |
| $[3Br@Pd_4\mathbf{L}^{\mathbf{octyl}}_8]^{5+}$ | 893 ± 3 | 968 ± 5 | +8.5 % |
| $[3Br@Pd_4\mathbf{L}^{\mathbf{decyl}}_8]^{5+}$ | 890 ± 3 | 989 ± 7 | +11.1 % |
| $[3Br@Pd_4\mathbf{L}^{\mathbf{dodecyl}}_8]^{5+}$ | 889 ± 3 | 982 ± 8 | +10.5 % |
| $[3Br@Pd_4\mathbf{L}^{\mathbf{octyl}}_8]^{5+}$ (5 ns) | 893 ± 3 | 965 ± 2 | +8.2 % |
| $[3Br@Pd_4\mathbf{L}^{\mathbf{decyl}}_8]^{5+}$ (5 ns) | 890 ± 3 | 978 ± 5 | +9.9 % |
| $[3Br@Pd_4\mathbf{L}^{\mathbf{dodecyl}}_8]^{5+}$ (5 ns) | 889 ± 3 | 974 ± 6 | +9.6 % |

### 2.2.7 $^{theo}CCS_{N2}$ of geometry optimized cage systems and according experimental values

**Table S6.** Experimental and theoretical CCS values as well as relative deviations for the cages that were treated with geometry optimizations.

| System | $^{TIMS}CCS_{N2}$ [Å²] | Conformation | $^{theo}CCS_{N2}$ [Å²] | $\Delta_{\%}$ |
|---|---|---|---|---|
| $[Pd_2\mathbf{L}^{methyl}{}_4]^{4+}$ | 582 ± 3 | open | 596.8 | +2.5 % |
| | | single-folded | 570.9 | –1.9 % |
| | | (double-)folded | 532.6 | –8.5 % |
| $[Pd_2\mathbf{L}^{methyl}{}_4 + BF_4]^{3+}$ | 542 ± 2 | open | 563.8 | +4.1 % |
| | | single-folded | 539.5 | –0.4 % |
| | | (double-)folded | 502.3 | –7.3 % |
| $[Pd_2\mathbf{L}^{methyl}{}_4 + 2BF_4]^{2+}$ | 520 ± 2 | open | 549.4 | +5.7 % |
| | | single-folded | 527.5 | +1.5 % |
| | | (double-)folded | 489.3 | –5.8 % |
| $[Pd_2\mathbf{L}^{phenyl}{}_4]^{4+}$ | 642 ± 3 | open | 667.0 | +4.0 % |
| | | single-folded | 637.8 | –0.6 % |
| | | (double-)folded, $D_2$ | 592.7 | –7.6 % |
| | | (double-)folded, $C_S$ | 592.4 | –7.7 % |
| $[Pd_2\mathbf{L}^{phenyl}{}_4 + BF_4]^{3+}$ | 600 ± 2 | open | 638.9 | +6.5 % |
| | | single-folded | 604.5 | +0.8 % |
| | | (double-)folded, $D_2$ | 564.2 | –5.9 % |
| | | (double-)folded, $C_S$ | 565.5 | –5.7 % |
| $[Pd_2\mathbf{L}^{phenyl}{}_4 + 2BF_4]^{2+}$ | 571 ± 1 | open | 625.8 | +9.6 % |
| | | single-folded | 598.5 | +4.8 % |
| | | (double-)folded, $D_2$ | 550.7 | –3.6 % |
| | | (double-)folded, $C_S$ | 550.0 | –3.7 % |
| $[Pd_2\mathbf{L}^{pyrenyl}{}_4]^{4+}$ | 691* | open | 807.3 | +16.9 % |
| | 691* | single-folded | 753.9 | +9.2 % |
| | 684 ± 3 | (double-)folded, $D_2$ | 679.6 | –0.7 % |
| | 697 ± 3 | (double-)folded, $C_S$ | 686.0 | –1.6 % |
| $[Pd_2\mathbf{L}^{pyrenyl}{}_4 + BF_4]^{3+}$ | 653* | open | 784.7 | +20.2 % |
| | 653* | single-folded | 735.9 | +12.7 % |
| | 651 ± 1 | (double-)folded, $D_2$ | 657.5 | +1.0 % |
| | 655 ± 2 | (double-)folded, $C_S$ | 662.7 | +1.2 % |

*These values are averages of the two overlapping signals, because that would make more sense for open or single-folded.

## 2.3 Geometry optimizations, numerical frequencies and single point energies

### 2.3.1 Headers

All calculations of this kind were performed with ORCA 5.0.2.[33] All geometry optimizations in this work were done with r$^2$SCAN-3c, if not noted otherwise.[29] The chemically relevant settings in the header of the input files are:

```
! r2SCAN-3c Opt
%geom trust -0.1 end
```

The numerical frequency calculations were also done with r²SCAN-3c. No inharmonic frequencies were found. The default temperature of 298 K was used as we believe this to be the temperature in the ESI source. The chemically relevant settings in the header of the input files are:

```
! r2SCAN-3c NumFreq
```

The chemically relevant settings in the header of the input files for energy calculations on ωB97M-D4/def2-TZVP or ωB97M-V/def2-TZVP level are:

```
! wB97M-D4 def2-TZVP TightSCF
```

or respectively

```
! wB97M-V def2-TZVP TightSCF
```

The chemically relevant settings in the header of the input files for calculations with HFLD are:

```
! HFLD cc-pVTZ cc-pVTZ/C RIJCOSX TightPNO VeryTightSCF
```

For the calculation of inter-fragment dispersion only cutouts of the optimized structures containing only one stacked pair of ligands and not palladium was used.

The chemically relevant settings in the header of the input files for calculations with DLPNO-CCSD(T)/TightPNO/def2-TZVP are:

```
! DLPNO-CCSD(T) def2-TZVP def2-TZVP/C RIJCOSX TightPNO VeryTightSCF
```

## 2.3.2 Optimized models

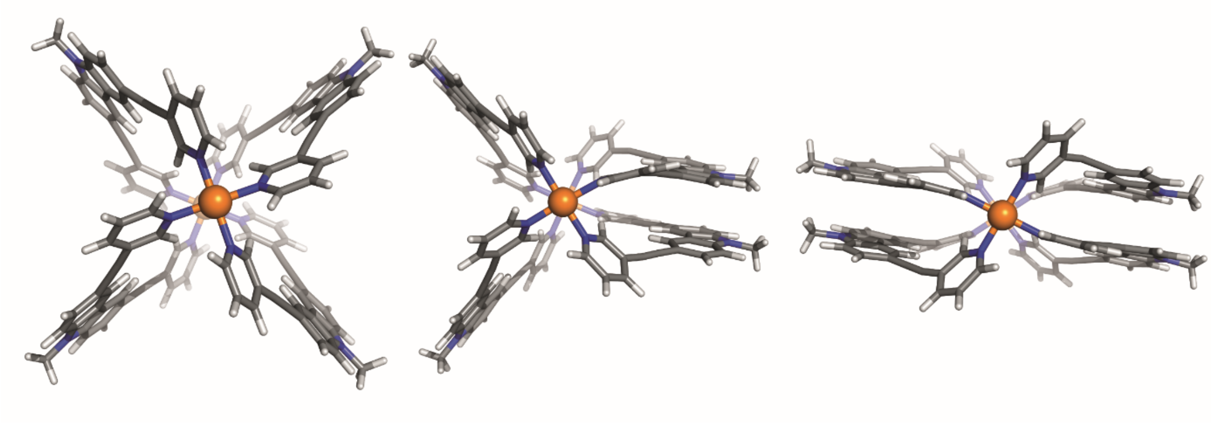

**Figure S40.** Geometry optimized models of $[Pd_2\mathbf{L}^{methyl}{}_4]^{4+}$ in different conformations. From left to right: open, single-folded, (double-)folded.

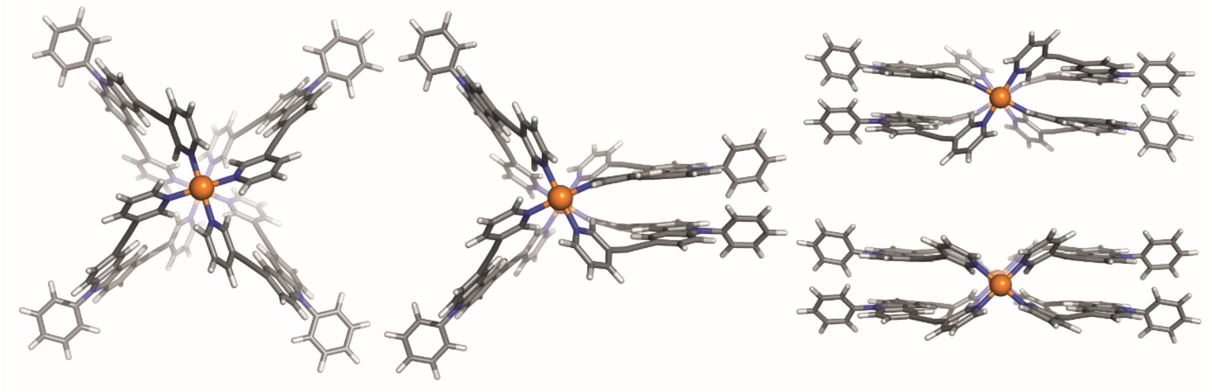

**Figure S41.** Geometry optimized models of $[Pd_2\mathbf{L}^{phenyl}{}_4]^{4+}$ in different conformations. From left to right: open, single-folded, (double-)folded. Upper left: (double-)folded, $C_S$-symmetric. Lower left: (double-)folded, $\underline{D_2}$-symmetric.

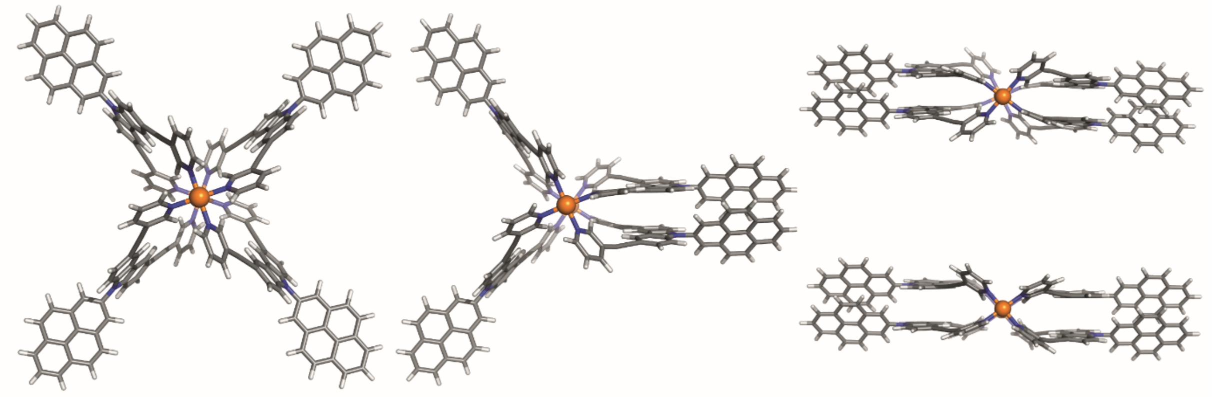

**Figure S42.** Geometry optimized models of $[Pd_2\mathbf{L}^{pyrenyl}{}_4]^{4+}$ in different conformations. From left to right: open, single-folded, (double-)folded. Upper left: (double-)folded, $C_S$-symmetric. Lower left: (double-)folded, $D_2$-symmetric.

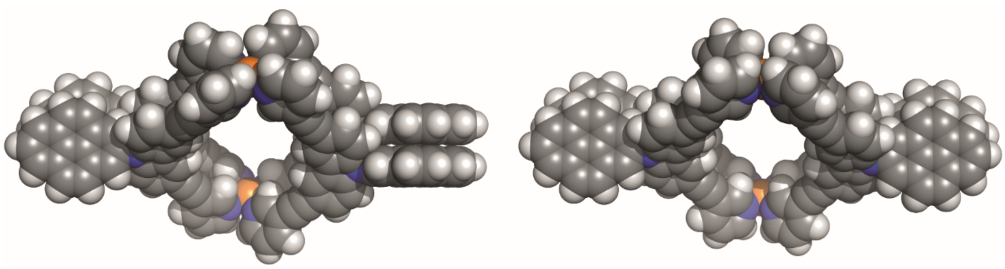

**Figure S43.** Space-filling models of optimized $[Pd_2\mathbf{L}^{pyrenyl}{}_4]^{4+}$ in (double-)folded, $C_S$-symmetric (left) and (double-)folded, $D_2$-symmetric (right).

## 2.4 Additional QM calculations

### 2.4.1 Energy calculations comparing D4 and VV10 and considering the single-folded conformer

Table S7 contains the electronic energy on ωB97M level (without dispersion correction) $E_{\omega B97M}$, the energy contribution of each dispersion correction $E_{D4}$ and $E_{VV10}$, the $G_{RRHO}$ term and the resulting free energies $G_{D4}$ and $G_{VV10}$, depending on the used dispersion correction. Given are the energy differences of the single-folded and double-folded conformer to the open conformer for $[Pd_2\mathbf{L}^{methyl}{}_4]^{4+}$, $[Pd_2\mathbf{L}^{phenyl}{}_4]^{4+}$ and $[Pd_2\mathbf{L}^{pyrenyl}{}_4]^{4+}$. In general, the relative differences between $\Delta E_{D4}$ and $\Delta E_{VV10}$ are low but the resulting differences in the free energies are more significant because in the case for $[Pd_2\mathbf{L}^{methyl}{}_4]^{4+}$ and $[Pd_2\mathbf{L}^{phenyl}{}_4]^{4+}$ $\Delta E_{D4}$ or $\Delta E_{VV10}$ gets mostly cancelled out by $\Delta E_{\omega B97M}$ and $\Delta G_{RRHO}$. As mentioned in the main text, according to $\Delta G_{D4}$ $[Pd_2\mathbf{L}^{phenyl}{}_4]^{4+}$ stays open but according to $\Delta G_{VV10}$ rather a balance between an open and a folded conformation may be possible. This would already statistically make a single-folded conformer possible. As can be seen, the considered energy terms for single-folded are always between those for open and double-folded and the energy increase or decrease from open to single-folded to double-folded is always nearly linear showing no sign of significant positive or negative cooperativity indicating, that, if the single-folded conformation for any of the three cages is present, it is only due to statistical reasons.

**Table S7.** Different energy terms calculated for the open, single-folded and double-folded (both variants) conformers of $[Pd_2\mathbf{L}^{methyl}{}_4]^{4+}$, $[Pd_2\mathbf{L}^{phenyl}{}_4]^{4+}$ and $[Pd_2\mathbf{L}^{pyrenyl}{}_4]^{4+}$. Unit is kJ/mol.

| System | Energy term | $E^{single\text{-}folded}$-$E^{open}$ | $E^{double\text{-}folded,D2}$-$E^{open}$ | $E^{double\text{-}folded,CS}$-$E^{open}$ |
|---|---|---|---|---|
| $[Pd_2\mathbf{L}^{methyl}{}_4]^{4+}$ | $\Delta E_{\omega B97M}$ | +66.7 | +135.1 | - |
| | $\Delta E_{D4}$ | –62.1 | –127.0 | - |
| | $\Delta E_{VV10}$ | –66.0 | –135.8 | - |
| | $\Delta G_{RRHO}$ | +6.5 | +13.3 | - |
| | $\Delta G_{D4}$ | +11.1 | +21.4 | - |
| | $\Delta G_{VV10}$ | +7.2 | +12.6 | - |
| $[Pd_2\mathbf{L}^{phenyl}{}_4]^{4+}$ | $\Delta E_{\omega B97M}$ | +71.2 | +144.1 | +147.9 |
| | $\Delta E_{D4}$ | –73.1 | –151.3 | –151.7 |
| | $\Delta E_{VV10}$ | –78.4 | –163.8 | –163.4 |
| | $\Delta G_{RRHO}$ | +6.6 | +16.1 | +17.5 |
| | $\Delta G_{D4}$ | +4.7 | +8.9 | +13.7 |
| | $\Delta G_{VV10}$ | –0.6 | –3.5 | +2.0 |
| $[Pd_2\mathbf{L}^{pyrenyl}{}_4]^{4+}$ | $\Delta E_{\omega B97M}$ | +81.1 | +167.1 | +172.8 |
| | $\Delta E_{D4}$ | –122.8 | –255.5 | –257.7 |
| | $\Delta E_{VV10}$ | –131.9 | –276.7 | –277.8 |
| | $\Delta G_{RRHO}$ | +10.2 | +26.7 | +28.5 |
| | $\Delta G_{D4}$ | –31.5 | –61.7 | –56.3 |
| | $\Delta G_{VV10}$ | –40.6 | –82.9 | –76.4 |

### 2.4.2 Geometry optimizations using different DFT methods, angle scans and CSD-searches

As mentioned in the main text, MD simulations with GFN2-xTB without constraints led to heavy distortions at the Pd centers. Especially problematic is the angle between the palladium atom, the donating nitrogen atom and the carbon atom in para position to the nitrogen atom (Pd–N–$C_{para}$). To check whether these distortions and therefore the folding of $[Pd_2\mathbf{L}^{pyrenyl}{}_4]^{4+}$ in the gas phase are realistic we performed motif searches for this angle in the Cambridge Structural Database (CSD 5.41) using a single Pd-pyridine group as motif. The results are shown in a form of a histogram in Figure S43. As can be seen, angles up to around 157° are rare but realistic. When specifying that "the Pd atom and the N atom must not be part of a circle", to avoid chelate complexes, far less results were found. Here, only examples up to around 168° are found.

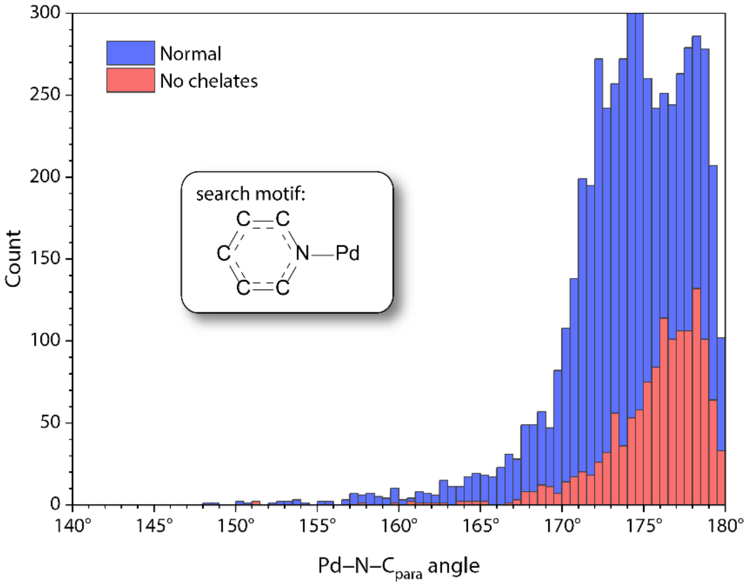


**Figure S44.** Result of a motif search in the CSD 5.41 database with and without specifying that "the Pd atom and the N atom must not be part of a circle".

We optimized the model of $[Pd_2\mathbf{L}^{pyrenyl}{}_4]^{4+}$ in double-folded conformation with GFN2-xTB, r$^2$SCAN-3c, ωB97M-D4/def2-TZVP and ωB97M-V/def2-TZVP. The average and smallest angles from the optimized models are listed in. As can be seen, the situation greatly improves with increasing level of theory with an average angle of 169.3° when optimized with ωB97M-V/def2-TZVP. The semiempirical method r$^2$SCAN-3c shows acceptable angles as well.

**Table S8.** Average and smallest Pd–N–$C_{para}$ angles of the double-folded model of $[Pd_2\mathbf{L}^{pyrenyl}{}_4]^{4+}$ optimized with different methods.

| | **GFN2-xTB** | **r$^2$SCAN-3c** | **ωB97M-D4/def2-TZVP** | **ωB97M-V/def2-TZVP** |
|---|---|---|---|---|
| Average | 156° ± 2° | 165.0° ± 0.9° | 168° ± 1° | 167° ± 1° |
| Minimum | 152.6° | 163.0° | 165.8 | 165.6° |

Additionally, we performed relaxed angle scans of that angle in a smaller $[Pd(pyridine)_4]^{2+}$ complex using either GFN2-xTB or r$^2$SCAN-3c. For higher level ωB97M-D4/def2-TZVP and DLPNO-CCSD(T)[34]/TightPNO/def2-TZVP energies the geometries from the scan with r$^2$SCAN-3c were taken.

The energy increase with increasing distortion of the Pd–N–$C_{para}$ angle is plotted in Figure S44. Apparently $r^2$SCAN-3c and ωB97M-D4 already perform very close to the reference, while GFN2-xTB performs poorly in this regard.

In GFN2-xTB MD simulations with $BF_4^-$ counter anions we noticed that the Pd complexes are even more flexible due to a charge transfer. We repeated the angle scans, this time with a $BF_4^-$ in close proximity to the complex and noticed an increased flexibility even for the higher methods. However, the flexibility rise decreases with increasing level of theory. In addition, we note that here geometry optimized structures are considered while in the experiment the $BF_4^-$ is constantly moving and not perfectly close to the Pd complex, which would further decrease any charge transfer.

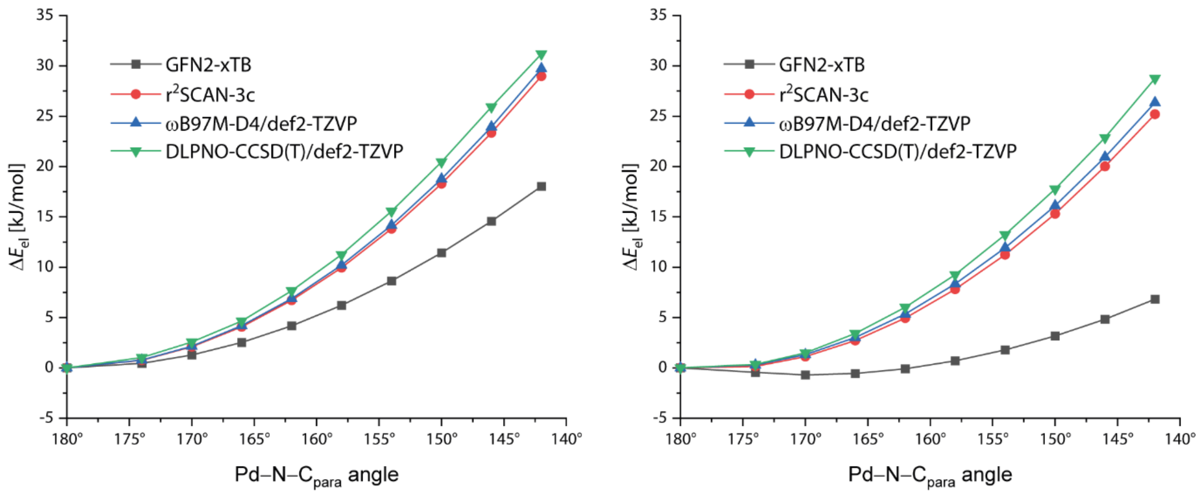


**Figure S45.** Relaxed angle scans for one of the Pd–N–$C_{para}$ angles in a) a $[Pd_2(pyridine)_4]^{2+}$ complex and b) a $[Pd_2(pyridine)_4]^{2+}$ complex with a $BF_4^-$ in proximity. For ωB97M-D4/def2-TZVP and DLPNO-CCSD(T)/TightPNO/def2-TZVP energies the geometries from the relaxed scan with $r^2$SCAN-3c were taken.

## References:

[1] A. Jerschow, N. Müller, *J. Magn. Reson.* **1997**, *125*, 372–375.

[2] A. Jerschow, N. Müller, *J. Magn. Reson.* **1996**, *123*, 222–225.

[3] E. O. Stejskal, J. E. Tanner, *J. Chem. Phys.* **1965**, *42*, 288–292.

[4] J. E. Tanner, E. O. Stejskal, *J. Chem. Phys.* **1968**, *49*, 1768–1777.

[5] A. Einstein, *Ann. Phys.* **1905**, *322*, 549–560.

[6] E. A. Mason, H. W. Schamp, *Ann. Phys.* **1958**, *4*, 233–270.

[7] E. A. Mason, E. W. McDaniel, Transport Properties of Ions in Gases, **2019**, John Wiley & Sons, Inc., DOI 10.1002/3527602852.

[8] S. M. Stow, T. J. Causon, X. Zheng, R. T. Kurulugama, T. Mairinger, J. C. May, E. E. Rennie, E. S. Baker, R. D. Smith, J. A. McLean, S. Hann, J. C. Fjeldsted, *Anal. Chem.* **2017**, *89*, 9048–9055.

[9] R. Zhu, J. Lübben, B. Dittrich, G. H. Clever, *Angew. Chem. Int. Ed.* **2015**, *54*, 2796–2800.

[10] A. Burkhardt, T. Pakendorf, B. Reime, J. Meyer, P. Fischer, N. Stübe, S. Panneerselvam, O. Lorbeer, K. Stachnik, M. Warmer, P. Rödig, D. Göries, A. Meents, *Eur. Phys. J. Plus* **2016**, *131*, 56.

[11] W. Kabsch, *Acta Cryst. D: Biol. Cryst.***2010**, *66*, 125–132.

[12] L. Potterton, J. Agirre, C. Ballard, K. Cowtan, E. Dodson, P. R. Evans, H. T. Jenkins, R. Keegan, E. Krissinel, K. Stevenson, A. Lebedev, S. J. McNicholas, R. A. Nicholls, M. Noble, N. S. Pannu, C. Roth, G. Sheldrick, P. Skubak, J. Turkenburg, V. Uski, F. von Delft, D. Waterman, K. Wilson, M. Winn, M. Wojdyr, *Acta Cryst. D: Struct. Biol.* **2018**, *74*, 68–84.

[13] G. M. Sheldrick, *Acta Cryst. C: Struct. Chem.* **2015**, *71*, 3–8.

[14] C. B. Hübschle, G. M. Sheldrick, B. Dittrich, *J. Appl. Cryst.* **2011**, *44*, 1281–1284.

[15] D. Kratzert, I. Krossing, *J. Appl. Cryst.* **2018**, *51*, 928–934.

[16] D. Kratzert, J. J. Holstein, I. Krossing, *J. Appl. Cryst.* **2015**, *48*, 933–938.

[17] A. L. Spek, *Acta Cryst. C: Struct. Chem.* **2015**, *71*, 9–18.

[18] A. L. Spek, *Acta Cryst. D: Biol. Cryst.* **2009**, *65*, 148–155.

[19] C. Bannwarth, E. Caldeweyher, S. Ehlert, A. Hansen, P. Pracht, J. Seibert, S. Spicher, S. Grimme, *Wiley Interdiscip. Rev.: Comput. Mol. Sci.* **2021**, *11*, DOI 10.1002/wcms.1493.

[20] C. Bannwarth, S. Ehlert, S. Grimme, *J. Chem. Theory Comput.* **2019**, *15*, 1652–1671.

[21] S. A. Ewing, M. T. Donor, J. W. Wilson, J. S. Prell, *J. Am. Soc. Mass Spectr.* **2017**, *28*, 587–596.

[22] A. V. Marenich, S. V. Jerome, C. J. Cramer, D. G. Truhlar, *J. Chem. Theory Comput.* **2012**, *8*, 527–541.

[23] S. Grimme, C. Bannwarth, P. Shushkov, *J. Chem. Theory Comput.* **2017**, *13*, 1989–2009.

[24] L. Zanotto, G. Heerdt, P. C. T. Souza, G. Araujo, M. S. Skaf, *J. Comput. Chem.* **2018**, *39*, 1675–1681.

[25] C. Larriba, C. J. Hogan, *J. Phys. Chem.* **2013**, *117*, 3887–3901.

[26] I. Campuzano, M. F. Bush, C. V. Robinson, C. Beaumont, K. Richardson, H. Kim, H. I. Kim, *Anal. Chem.* **2012**, *84*, 1026–1033.

[27] J. W. Lee, H. H. L. Lee, K. L. Davidson, M. F. Bush, H. I. Kim, *Analyst* **2018**, *143*, 1786–1796.

[28] C. Ieritano, J. Crouse, J. L. Campbell, W. S. Hopkins, *Analyst* **2019**, *144*, 1660–1670.

[29] S. Grimme, A. Hansen, S. Ehlert, J.-M. Mewes, *J. Chem. Phys.* **2021**, *154*, 064103

[30] T. Wu, J. Derrick, M. Nahin, X. Chen, C. Larriba-Andaluz, *J. Chem. Phys.* **2018**, *148*, 074102.

[31] M. Han, R. Michel, B. He, Y. Chen, D. Stalke, M. John, G. H. Clever, *Angew. Chem. Int. Ed.* **2013**, *52*, 1319–1323.

[32] A. Grossfield, D. M. Zuckerman, *Annu. Rep. Comput. Chem.* **2009**, *5*, 23–48.

[33] F. Neese, *Wiley Interdiscip. Rev.: Comput. Mol. Sci.* **2022**, *12*, DOI 10.1002/wcms.1606.

[34] Y. Guo, C. Riplinger, U. Becker, D. G. Liakos, Y. Minenkov, L. Cavallo, F. Neese, *J. Chem. Phys.* **2018**, *148*, 011101.
